# Classifying grass-dominated habitats from remotely sensed data: The influence of spectral resolution, acquisition time and the vegetation classification system on accuracy and thematic resolution

**DOI:** 10.1016/j.scitotenv.2019.134584

**Published:** 2020-04-01

**Authors:** Ute Bradter, Jerome O'Connell, William E. Kunin, Caroline W.H. Boffey, Richard J. Ellis, Tim G. Benton

**Affiliations:** University of Leeds, School of Biology, Leeds LS2 9JT, UK

**Keywords:** Hyperspectral, Mapping, Multispectral, National Vegetation Classification (NVC), Random forest, Vegetation community

## Abstract

Detailed maps of vegetation facilitate spatial conservation planning. Such information can be difficult to map from remotely sensed data with the detail (thematic resolution) required for ecological applications.

For grass-dominated habitats in the South-East of the UK, it was evaluated which of the following choices improved classification accuracies at various thematic resolutions: 1) Hyperspectral data versus data with a reduced spectral resolution of eight and 13 bands, which were simulated from the hyperspectral data. 2) A vegetation classification system using a detailed description of vegetation (sub)-communities (the British National Vegetation Classification, NVC) versus clustering based on the dominant plant species (Dom-Species). 3) The month of imagery acquisition.

Hyperspectral data produced the highest accuracies for vegetation away from edges using the NVC (84–87%). Simulated 13-band data performed also well (83–86% accuracy). Simulated 8-band data performed poorer at finer thematic resolutions (77–78% accuracy), but produced accuracies similar to those from simulated 13-band or hyperspectral data for coarser thematic resolutions (82–86%). Grouping vegetation by NVC (84–87% accuracy for hyperspectral data) usually achieved higher accuracies compared to Dom-Species (81–84% for hyperspectral data). Highest discrimination rates were achieved around the time vegetation was fully developed.

The results suggest that using a detailed description of vegetation (sub)-communities instead of one based on the dominating species can result in more accurate mapping. The NVC may reflect differences in site conditions in addition to differences in the composition of dominant species, which may benefit vegetation classification. The results also suggest that using hyperspectral data or the 13-band multispectral data can help to achieve the fine thematic resolutions that are often required in ecological applications. Accurate vegetation maps with a high thematic resolution can benefit a range of applications, such as species and habitat conservation.

## Highlights

• Many ecological applications require vegetation maps at high thematic resolution.
• Categorizing by vegetation (sub)-communities was better than by dominating species.
• Higher thematic resolution from hyperspectral or 13-band than 8-band data.
• Highest differentiation between categories when vegetation was fully developed.

## Graphical abstract

**Figure:**
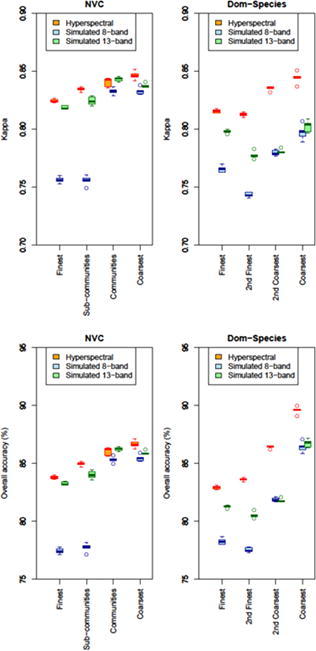

## 1 Introduction

Biodiversity continues to decline through a variety of anthropogenic drivers (Butchart et al., 2010, Tittensor et al., 2014) and land is under substantial pressure through an increasing human population and rising demand for goods and services (Foresight, 2011, Smith et al., 2010). Managing land better to maintain a high level of biodiversity will be most efficient when planning takes into account the effect of complex landscapes on ecological systems and on species (Benton, 2012). Detailed maps of vegetation can facilitate such planning.

Remote sensing has become an important tool to map and monitor biodiversity, see e.g. Kuenzer et al. (2014). Optical remote sensing makes use of the differences in reflectance caused by the variation in chemical composition of plants, the structure of plant tissue and the plant canopy to assess characteristics of plants such as plant stress or to map vegetation (Lillesand et al., 2008, Thenkabail et al., 2012). However, remotely sensed maps often depict broad habitat categories and do not always provide the level of detail (thematic resolution henceforth) needed in ecological applications (Bradley and Fleishman, 2008). A fine thematic resolution can be difficult to achieve, especially in particular habitats. Grasslands, for example, can contain a diverse range of species and individual plant species are relatively small compared to the spatial resolution of imagery sensors. Indeed, a horizon scan in 2014 pointed out the need for better differentiation and monitoring of ‘more difficult’ habitats, such as native grasslands (Sutherland et al., 2014).

**Fig. 1:**
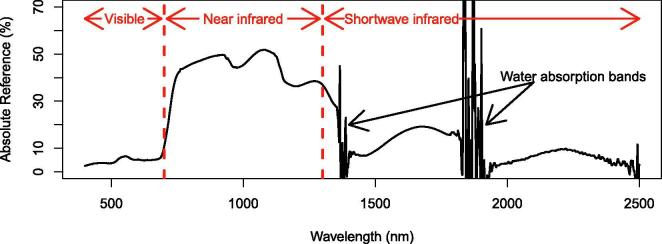
An example of reflectance of a grassland measured with a hyperspectral sensor.

Hyperspectral data has great potential for the differentiation of habitats as it allows the detection of more subtle differences in canopy reflectance compared to multispectral data (Thenkabail et al., 2012). Hyperspectral sensors measure radiance in many narrow bands of the electromagnetic spectrum (Fig. 1) in contrast to widely used multispectral platforms, such as the satellite platforms Landsat or Sentinel-2, which return reflectance from fewer and broader bands (Lillesand et al., 2008, Thenkabail et al., 2012). The disadvantages of hyperspectral data are its limited availability particularly at very high spatial resolutions and the lower signal-to-noise ratio that may occur with narrow bandwidths (Thenkabail et al., 2012). Current sources of hyperspectral data are the airborne AVIRIS (https://aviris.jpl.nasa.gov) and the EO1-Hyperion satellite sensor (https://archive.usgs.gov/archive/sites/eo1.usgs.gov/hyperion.html). Further satellite missions are in preparation, such as EnMap (www.enmap.org) and HyspIRI (https://hyspiri.jpl.nasa.gov). These satellite platforms have spatial resolutions between 30 m (EO1-Hyperion, EnMap) and 60 m (HyspIRI). Very high spatial resolutions are provided on demand by airborne or hand-held sensors.

Hyperspectral data have been used to map vegetation in case studies at a fine thematic resolution, even in grasslands. Floristic gradients (i.e. species composition) within broader habitat types have been mapped (Feilhauer and Schmidtlein, 2011, Harris et al., 2015). Successional stages in dry grassland were grouped into age-classes (Möckel et al., 2014). Plant functional types (Ellenberg indicator values/Grime’s CSR strategy types) were predicted for mountainous grassland (Schweiger et al., 2017) and wetlands (Schmidtlein et al., 2012). Vegetation plots dominated by a single species were differentiated for four species in South African rangeland (Mansour et al., 2012).

While the many narrow and adjacent bands of hyperspectral data contain a lot of information, they also contain redundant information (Thenkabail et al., 2012). Analysing large hyperspectral data sets can be time-consuming and this cost could be reduced if redundant bands were known and could be removed before data collection or before analysis. However, the knowledge of how vegetation reflectance varies with the vegetation under consideration is incomplete (Kattenborn et al., 2018). Certain bands are well known to contribute to the differentiation of vegetation, but additional bands may further improve the vegetation differentiation. Moreover, the structure and chemical composition of plants can vary during the vegetation cycle as may the species composition of communities (Feilhauer and Schmidtlein, 2011). This can lead to changes in the spectral characteristics of vegetation during the year (Lillesand et al., 2008). Therefore, the optimal band combination leading to the highest vegetation differentiation may vary with the vegetation considered and with time of year. The ability with which vegetation categories can be discriminated may also be higher at certain time periods of the year compared to others.

Another fundamental consideration in vegetation mapping is how to describe vegetation. Plant community composition shifts in a continuous manner (Austin and Smith, 1989). Nonetheless, discrete vegetation categories are often preferred by conservation practitioners and land managers due to their comparable ease of use. An example of such a categorization is the National Vegetation Classification (NVC) (Rodwell, 1991–2000), which is widely used in Great Britain for habitat mapping and ecological surveying (Hearn et al., 2011). National vegetation classification systems exist in several countries, for example the United States National Vegetation Classification (USNVC; http://usnvc.org) or the Irish Vegetation Classification (IVC; http://www.biodiversityireland.ie/projects/national-vegetation-database/irish-vegetation-classification/). The NVC corresponds to a phytosociological classification: certain combinations of plant species and their abundances are indicative of certain conditions (soil, land use, etc.) (Rodwell, 2006). NVC surveys on the ground can be time-consuming as survey instructions suggest the recording of all plant species and their abundances in several sample plots (Rodwell, 2006). In practice surveyors may use different, faster techniques and maps recorded by different surveyors can show substantial differences (Hearn et al., 2011). Therefore, it may be attractive to use an alternative vegetation grouping, potentially requiring less field survey effort and producing less variation between surveyors, for example by considering only the most abundant plant species.

Further considerations in vegetation mapping are the size of the objects to be mapped. In intensively used agricultural areas, many of the non-arable objects are comparatively narrow, for example ditches or grass strips along fields. Despite their small size they may be important as they often contain much of the local biodiversity (Gabriel et al., 2010) and can enhance populations of species that provide ecosystem services such as pest control (Rouabah et al., 2015) or pollination (Rands and Whitney, 2011). Some narrow objects contain vegetation categories which, at least in intensively used agricultural landscapes, rarely cover a large extent, such as wild-flower rich roadside vegetation or nettle (*Urtica dioica*) patches. Very high spatial resolution imagery is often used to map vegetation in narrow objects, which requires airborne rather than satellite sensors (Lillesand et al., 2008).

The aim of this study was to evaluate how using hyperspectral data in comparison to multispectral data and how the vegetation classification system influences thematic resolution and accuracy of vegetation differentiation and hence to provide information that facilitates the creation of vegetation maps. An agricultural landscape was chosen as study area, because agriculture is a major driver of biodiversity loss and affects a large proportion of the global land area (Kehoe et al., 2015), creating a need for habitat mapping and appropriate land management to mitigate these influences. Specifically:

1) The influence of thematic resolution on the accuracy with which vegetation categories were differentiated was evaluated using hyperspectral imagery.
2) It was evaluated if using a lower spectral resolution produced similar results as the hyperspectral data.
3) Vegetation mapping using the NVC was evaluated versus a grouping based on the dominating plant species.
4) The sensitivity of the results to (a) narrow objects and (b) the acquisition month was assessed.

## 2 Methods

### 2.1 Overview

The study was carried out in an intensively used agricultural area in East Anglia, UK, focusing on grass-dominated habitats, including in narrow objects such as margins and ditches. Three types of remotely-sensed data were used: 1) aerial hyperspectral data, 2) the aerial hyperspectral data resampled to two coarser spectral resolutions and 3) hyperspectral data collected with hand-held spectroradiometers.

To evaluate how the thematic resolution influenced the accuracy with which vegetation categories were differentiated, aerial hyperspectral data were used. Due to the presence of narrow objects (ditches, margins, etc.) in the study area 1 m × 1 m spatial resolution imagery was used.

To evaluate if a lower spectral resolution produced similar results as the hyperspectral data, the aerial hyperspectral data were resampled to reduced spectral resolutions: (a) the 13 bands of the Sentinel-2 satellite sensor (‘simulated 13-bands’ henceforth) and (b) the eight bands (excluding the pan-chromatic band) of the Landsat satellite 8 OLI sensor (‘simulated 8-bands’ henceforth). Both Sentinel-2 data and Landsat data are widely used (Lewis, 1998, Pal, 2005, Pettorelli et al., 2014, Reid and Quarmby, 2000, Sesnie et al., 2008, Sluiter and Pebesma, 2010) and the spectral resolution of the Sentinel-2 sensor is particularly suitable for vegetation mapping (see e.g. Feilhauer et al., 2013, Rapinel et al., 2019). The reduced spectral resolution data were simulated from the hyperspectral data in order to attain the same spatial resolution as in the hyperspectral data (1 m × 1 m). Using satellite instead of simulated data would have confounded the spectral resolution comparison with spatial resolution effects because the spatial resolution of the satellite data (10–60 m for Sentinel-2 and 30 m for Landsat) is large relative to some of the objects in our study area.

Vegetation mapping using the NVC versus a grouping based on the dominating plant species was evaluated using both the hyperspectral data and the simulated lower spectral resolution data.

The sensitivity of the results to narrow objects was evaluated by comparing classifications of vegetation in narrow objects versus vegetation in broader objects using the airborne hyperspectral and simulated lower spectral resolution data. Additionally, hyperspectral data from narrow objects were collected with hand-held spectroradiometers carefully positioned to avoid the object edges.

The sensitivity of the results to the acquisition month was assessed using hyperspectral data acquired with hand-held sensors at approximately monthly intervals. Acquiring aerial hyperspectral data on a monthly basis would have been too costly and was not feasible.

Vegetation was ground-truthed via field visits in which all vascular plant species and their abundances were recorded in several sample plots per vegetation category. All classifications were carried out with the machine learning algorithm random forest (Breiman, 2001, Liaw and Wiener, 2002).

### 2.2 Remotely-sensed data

#### 2.2.1 Aerial hyperspectral data

**Fig. 2:**
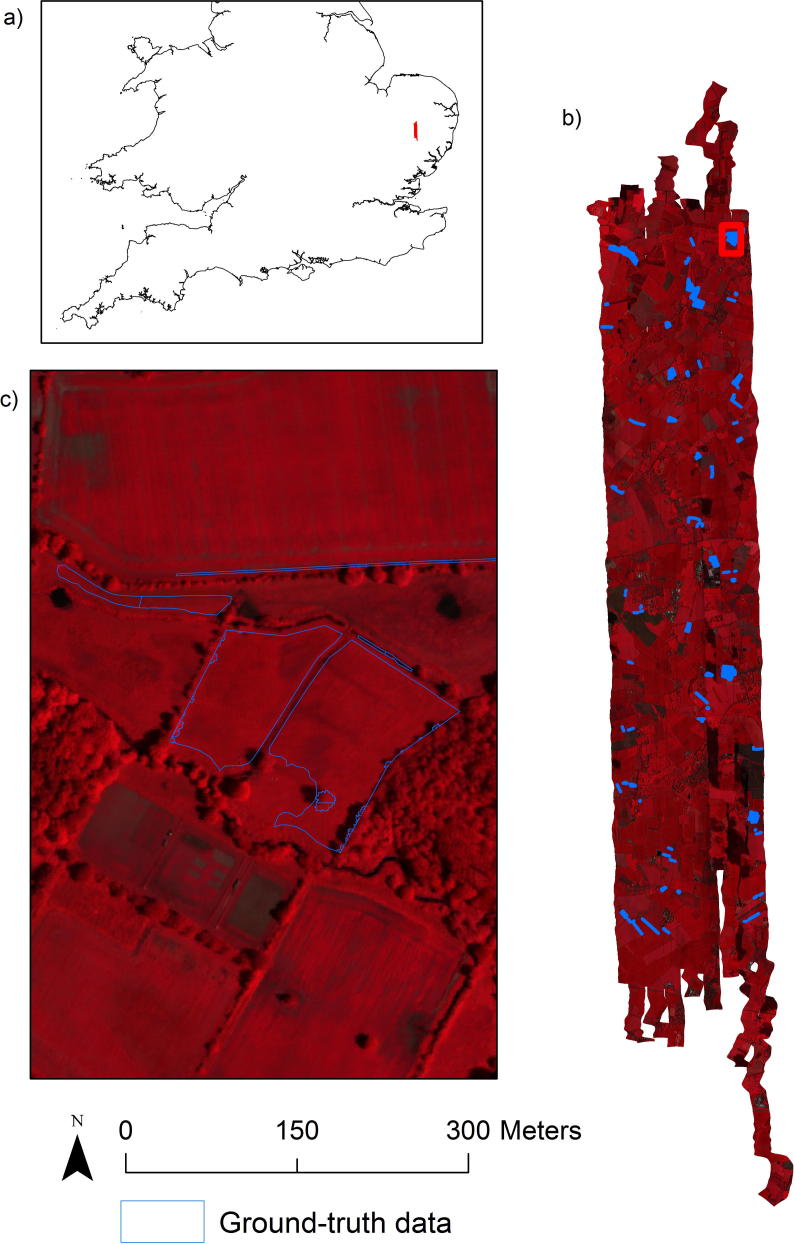
a) The location of the study area (red) in the south of Great Britain; b) A false colour RGB image of the hyperspectral airborne data for the study area together with the location of ground-truth data (blue polygons). The false colour image is an RGB composite of three bands from the near-infrared (865 nm), red (655 nm) and green (562 nm); c) An enlargement of the area in the red rectangle in b). Copyright 2016 NERC. (For interpretation of the references to colour in this figure legend, the reader is referred to the web version of this article.)

Hyperspectral data at a spatial resolution of 1 × 1 m and with a georeferencing accuracy of ca. 2 m (Fig. 2, see Table 1 for data characteristic and details on the date and time of acquisition) were collected on the 12th and 13th of June 2014 over 63.3 km^2^ of predominately agricultural land in East Anglia, UK. The data was acquired by the NERC Airborne Research and Survey Facility (ARSF), UK using the AISA Fenix hyperspectral sensor (Specim, 2016). Post processing from radiance to reflectance by the ARSF Data Analysis Node at the Plymouth Marine Laboratory, UK included radiometric, atmospheric and cloud shadow correction with ATCOR-4 (Richter and Schläpfer, 2016) using in-built parameters of ATCOR-4, mosaicking of flightlines and mapping to British National Grid. ATCOR-4 is based on calculations of the MODTRAN 5 radiative transfer model (Berk et al., 1998). The correction results were visually validated against spectra from three large homogenous tarpaulins (black, grey and white) and from homogeneous grass vegetation, which were recorded on the ground with hand-held spectroradiometers during the duration of the airborne data capture.

**Table 1:** Characteristics of the hyperspectral data.

Data characterisitcs	Airborne data	Field spectroscopy data^a^
Number of spectral bands	622	1024 (SVC HR-1024i) − 2100 (resampled; ASD Field Spec Pro)
Spectral range	400–2500 nm	400–2500 nm
Spectral resolution	3.5–12 nm	3–12 nm (ASD Field Spec Pro)
		3.5–6.5 nm (SVC HR-1024i)
Spatial resolution	1 m × 1 m	NA
Field of view (diameter)	NA	31.5 cm (ASD Field Spec Pro with 18° fore-optic)
		13.9 cm (SVC HR-1024i with 8° fore-optic)
Date of acquisition	12th & 13th June 2014	May 2013 (1st–3rd)
		June 2013 (27th May–7th June)
		July 2013 (6th–19th)
		August 2013 (31st)
		September 2012 (7th–15th)
Time of acquisition	8:10–9:45 UTC + 1	2–3 h before and after solar noon
	(due to cloud cover at more optimal acquisition times around solar noon)	

a A single spectrum was recorded as an average of repeat scans, which reduces random noise.

#### 2.2.2 Simulated multispectral data

The spectral resolution of the hyperspectral airborne data was resampled to the lower multispectral resolutions of (1) the simulated 13-bands and (2) the simulated 8-bands. See Appendix A for band centres and bandwidths. Resampling based on the spectral response functions for the Sentinel-2 and Landsat 8 OLI sensors was carried out with the spectral resampling tool of ENVI 5.3 (http://www.harrisgeospatial.com/SoftwareTechnology/ENVI.aspx). This tool uses a Gaussian weighted curve based on the band spacing, wavelengths and spectral response of the satellite sensor in question to ensure that the resampled data was as close as possible to the spectral profile of the sensors. The spatial resolution of the hyperspectral data was retained in the final resampled image. The spectral response specifications are provided in Appendix B (simulated 13-bands) and C (simulated 8-bands).

#### 2.2.3 Hand-held spectroradiometer data

In order to investigate the influence of the acquisition month on accuracy of vegetation classification, hyperspectral data were collected with hand-held spectroradiometers from vegetation on four farms in East Anglia (see Table 1 for sensor characteristics and details on the dates and times of data acquisition). Spectra (Bradter et al.) were collected in 2012 with the ASD Field Spec Pro (Analytical Spectral Devices, Inc, Boulder, USA) and in 2013 with the SVC HR-1024i (Spectra Vista Corporation, New York, USA).

Spectroradiometers were supplied by the Natural Environment Research Council (NERC) Field Spectroscopy Facility (FSF), UK. Immediately before or after each vegetation spectrum, a reflectance spectrum from a Spectralon reference panel (Labsphere, North Sutton, USA) was collected, and absolute reflectance calculated. Spectroradiometers and reference panels were maintained and calibrated by FSF. Spectroradiometer calibration consisted of radiance and irradiance calibration, using standards calibrated by the National Physical Laboratories, and of wavelength verification (for further details see: https://fsf.nerc.ac.uk/lab/). For principles of field spectroscopy and guidelines on recording measurements see e.g. Mac Arthur et al., 2012, Mac Arthur et al., 2007, Milton, 1987.

Per vegetation object one spectrum was collected every ca. 9 m, unless the category occurred in small patches when distances between samples were reduced accordingly (see Table 2 for the number of sample points per month and Bradter et al.). The field of view of the spectroradiometers (0.02–0.08 m^2^; Table 1) was smaller than the pixel size of the airborne hyperspectral data (1 m^2^). However it was still large compared to individual plants within the field of view and always covered groups of several plants. Data collection was repeated at approximately monthly intervals during the growing season during sunny conditions (Table 1; Bradter et al.). If some clouds were present, care was taken to record the spectra when no clouds were near the sun. The number of categories (2–10 per month, median: 8) for which spectra were recorded, and the sample size per category depended on weather conditions (Table 2; Bradter et al.).

**Table 2:** Field spectroscopy data recording and classification: the overall number of spectra and categories recorded per month, the number of categories in the vegetation classification with the minimum, medium and maximum number of spectra per classification categories, and the classification accuracy (OOB accuracy) obtained with random forest.

Month	Number of spectra	Recorded categories	Categories in classification	Min/median/max number of spectra per classification category	OOB accuracy (%)
May	172	8	7	11/21/55	75
June	271	10	9	20/30/50	85
July	244	10	10	14/20/60	84
August	30	2	2	10/15/20	94
September	132	2	2	54/66/78	97

### 2.3 Spectral covariates

For the hyperspectral data the following covariates were calculated:

1) The first derivative, which is more independent of the background reflectance (e.g. soil) than reflectance (Demetriades-Shah et al., 1990). To counteract the amplification of noise that occurs with the derivation, smoothing with Savitzky-Golay filtering (Ruffin et al., 2008) was applied. Several smoothing levels were tested and a level resulting in the highest vegetation classification accuracy was selected, see Appendix D. Higher-order derivatives were not used as they are even more sensitive to noise (Demetriades-Shah et al., 1990).
2) The position and value of the minimum reflectance in the region 660–750 nm, which are related to foliage chlorophyll levels (Miller et al., 1991).
3) The positions and values of maximum or minimum first derivatives in the following wavelength regions as an association with plant characteristics was found, which may help to differentiate between vegetation categories (Pu et al., 2004): 495–550 nm, 550–650 nm, 970–1090 nm, 1110–1205 nm, 1205–1285 nm.
4) The vegetation indices summarized by Roberts et al. (2012), see Appendix E.
5) The local maxima in the first derivative between 690 and 750 nm (red-edge peaks) (Smith et al., 2004) as they have contributed to distinguishing between vegetation types (e.g. Boochs et al., 1990). For hand-held spectroscopy data only (due to their greater sensitivity), the wavelengths of the red-edge peaks, the local maximum reflectance and their ratios were calculated.

This resulted in 568 covariates for the airborne data, 1887 covariates for the 2012 and 919 covariates for the 2013 field spectroscopy data.

For the simulated data, the following covariates were used:

1) The reflectance of all bands.
2) The ratios between the reflectance of all bands as they are more robust to differences in illumination (e.g. shading) compared to reflectance (Lillesand et al., 2008).
3) The vegetation indices typically used with such bands and described in the product guides for Sentinel-2 and Landsat data (Appendix F).

To avoid wrongly discarding covariates for which an association with our vegetation categories is unknown or that have weaker predictive ability, no pre-selection among the covariates was carried out as the accuracy of predictions with random forest does not necessarily improve with variable selection (Hapfelmeier and Ulm, 2013).

### 2.4 Vegetation data

#### 2.4.1 Airborne study area

The study area consists of an intensively managed agricultural landscape dominated by arable crops and interspersed by grasslands (mainly pastures and meadows) and abundant narrow objects (field margins, road margins, ditches, etc.). Ground-truth vegetation data in the airborne study area were collected by two surveyors skilled in plant identification in June 2015, a year after the imagery was recorded (Table 1). Due to this time lag, surveyors took care to record only areas with vegetation communities, which persist for several years, and which only change slowly into other vegetation communities, for example as a result of vegetation succession or changes in hydrology (Appendix G). Rainfall varies considerably from year to year in the study area. It was average in the month of imagery acquisition and below average, but not extreme, in the month of vegetation recording (Appendix H). The temperature variation is low in the study area (Appendix H).

At each farm, surveyors mapped an area (“patch” henceforth) with relatively uniform species composition and structure (“type” henceforth). Then they mapped a type that was different from the first and so on. If feasible, more than one patch per type and farm was mapped. Ground-truth information at the edges of flightlines where the imagery showed artefacts was removed. In total, 34.3 ha in 116 patches of 83 types on 24 farms were used as ground-truth vegetation data. For each type and farm, all vascular plant species and their percentage cover were recorded in two sample areas of 4 m^2^ (exceptionally three, if vegetation was highly variable, or one, if there was only one small patch). For details, see Appendix I.

##### 2.4.1.1 Vegetation edges

Remotely-sensed mapping of vegetation can be difficult at edges due to mapping inaccuracies, mixed vegetation types in one pixel (Cracknell, 1998), continuous vegetation changes that are discretized for practical purposes (Rocchini et al., 2013) or scattering of reflectance from nearby objects (Otterman and Fraser, 1979). To assess the influence of vegetation edges on classification accuracy and thematic resolution, the ground-truth dataset was divided into one dataset (“Wide” henceforth) without vegetation edges and one dataset (“Narrow” henceforth) in which vegetation edges were frequent. Wide consisted of patches wider than 5 m from which the outer 2 m were removed (56 patches of 45 types on 22 farms). Narrow consisted of all types that were not in Wide (58 patches of 38 types on 18 farms).

##### 2.4.1.2 Thematic resolutions

Vegetation was assigned into categories at four thematic resolutions, based either on the NVC or on the three dominant species (Dom-Species henceforth).

Assignment to NVC communities was carried out using Tablefit (Hill, 1996) and identification keys and community descriptions in 'British Plant Communities' (Rodwell, 1991–2000). The software program Tablefit matches vegetation data to NVC communities, providing the five best fitting NVC (sub)-communities and goodness-of-fit scores. Goodness-of-fit scores can be quite similar between the five suggestions and the final choice was made using the NVC identification keys and plant community descriptions, if possible to sub-community level. Exceptionally, two NVC sub-communities were assigned if the vegetation had characteristics of either category. Some types could not be assigned a NVC community as some artificial habitats are not included in the NVC (Rodwell, 2006). NVC was assigned to 93 patches of 66 types on 23 farms.

The following four thematic resolutions were produced:

(i) Coarsest: NVC communities (Appendix G), with all S communities (swamps and tall-herb fens) grouped as one category as there were few (ten categories in Wide, nine in Narrow).
(ii) Communities: NVC communities (12 categories in Wide, 11 in Narrow).
(iii) Sub-communities: NVC sub-communities if possible, otherwise NVC communities (18 categories in Wide, 19 in Narrow).
(iv) Finest: as in (iii). In MG1, the largest sub-community MG1a was additionally subdivided by the dominating grass species if possible (20 categories in Wide, 23 in Narrow).

Dom-Species was categorised using clustering. For each 4 m^2^ sample area, the percentage cover of the three most abundant plant species was used in an unsupervised classification with the algorithm random forest (Breiman, 2001, Liaw and Wiener, 2002) following Shi and Horvarth (2006), see Appendix J. Four thematic resolutions were created: finest: 17 categories in Wide, 18 in Narrow; 2nd finest: nine categories in Wide, ten in Narrow; 2nd coarsest: seven categories; coarsest: four categories.

#### 2.4.2 Field spectroscopy study area

Vegetation ground-truth data (Bradter et al.) were collected from all categories in which field spectroscopy data were recorded using the same sampling scheme as for the airborne study area (see 2.4.1 Airborne study area). Vascular plant species were recorded in up to four sample areas of 4 m^2^ per category per year. The vegetation ground-truth data were collected in the same year the spectra were recorded (Table 1). There were no precipitation extremes in the months the spectra were collected (Appendix K). One thematic resolution was investigated (categories as recorded in field mapping). For commoner categories, up to three spatially distinct patches (e.g. three margins) were selected and for rarer categories, one patch (average: 1.9 patches).

### 2.5 Classification and validation

#### 2.5.1 Classification with random forest

Random forest was used for classification as hyperspectral data have a large number of covariates with many correlations. Random forest is robust to such data (Grömping, 2009, Strobl et al., 2008). It is a machine learning algorithm constructing an ensemble of regression or classification trees and aggregating the results (Breiman, 2001, Liaw and Wiener, 2002). It has resulted in good accuracies in vegetation classifications (Bradter et al., 2011, Chapman et al., 2010, O'Connell et al., 2015, Pal, 2005) and in classifications with a large number of covariates (Bradter et al., 2013). For a description of random forest, see e.g. Breiman, 2001, Grömping, 2009, Liaw and Wiener, 2002, Strobl et al., 2009. The analysis was carried out in R 3.4.1 (R Core Team, 2016) with package randomForest (Liaw and Wiener, 2002).

Random forest with an ensemble of classification trees was used. Random forest requires two tuning parameters, the number of trees in an ensemble (ntree) and the number of covariates to be tried at each node split (mtry). Higher ntree settings result in less variability and more stable accuracies (Genuer et al., 2010, Liaw and Wiener, 2002). The default value in the R package random forest is ntree = 500 (Liaw and Wiener, 2002). For the airborne data, ntree = 2000 were used to increase stability of the accuracy values. For mtry, the default value (√p where p is the number of covariates, Liaw and Wiener, 2002) was used (see Section 2.5.1.2 for the effect on random forest results of varying mtry). For ntree and mtry values in the variable selection for field spectroscopy data, see Section 2.5.1.2.

##### 2.5.1.1 Data imbalance

Categories with more data tend to get classified with a higher accuracy at the expense of categories with less data in random forest (Chen et al., 2004, Lin and Chen, 2012). Categories with large sample sizes in the airborne data were consequently downsampled, which better balances the errors (Chen et al., 2004, Lin and Chen, 2012), as sample size for common categories was larger than for rarer categories. In downsampling, for each classification tree in the ensemble, larger categories were randomly reduced. Downsampling was to 1000 (NVC with Wide), 1500 (Dom-Species with Wide) and 300 pixels (Narrow) per category. The total number of pixels, depending on the thematic resolution, was 6815 – 11,577 for NVC with Wide, 4544 – 18,628 for Dom-Species with Wide, 1930 – 3660 for NVC with Narrow and 1002 – 3289 for Dom-Species with Narrow (see Appendix L).

##### 2.5.1.2 Bands important for vegetation differentiation

To gain further understanding of the spectral covariates that were important in the differentiation of the vegetation categories, covariate selection was carried out. Random forest provides variable importance measures (Strobl et al., 2008, Strobl et al., 2007), thus identifying the covariates, which are important in a classification. The random forest variable selection of Genuer et al. (2010) was used to identify the bands which were important to differentiate our vegetation categories. This variable selection ranks covariates according to their permutation importance and, adding covariates one by one in a forward selection, retains those that improve classification accuracies (for full details see Appendix M). The permutation importance is calculated by randomly permuting each covariate in turn to destroy a potential association with the response variable. It is calculated as the difference in ‘out-of-bag’ (OOB) error from the model with the permuted covariate compared to the OOB error from the model without permutations. The OOB error is calculated on the approximately one-third of data that are randomly withheld in the construction of each tree in the ensemble (Breiman, 2001, Liaw and Wiener, 2002).

Downsampling led to high variation in the selected covariates between repeat runs. Therefore, covariates important for vegetation differentiation were identified in the classifications of field spectroscopy data, as these required no downsampling, but not for the classifications with aerial data, which required downsampling. For the tuning parameters ntree and mtry, the values proposed by Genuer et al. (2010) were used: for the initial ranking, ntree = 2000 and mtry = p/2 (p: number of covariates); for the forward selection, default ntree and mtry. The value of mtry effects the permutation importance, which becomes more conditional with higher mtry values and more marginal with lower mtry values (Grömping, 2009, Strobl et al., 2008).

#### 2.5.2 Validation

**Fig. 3:**
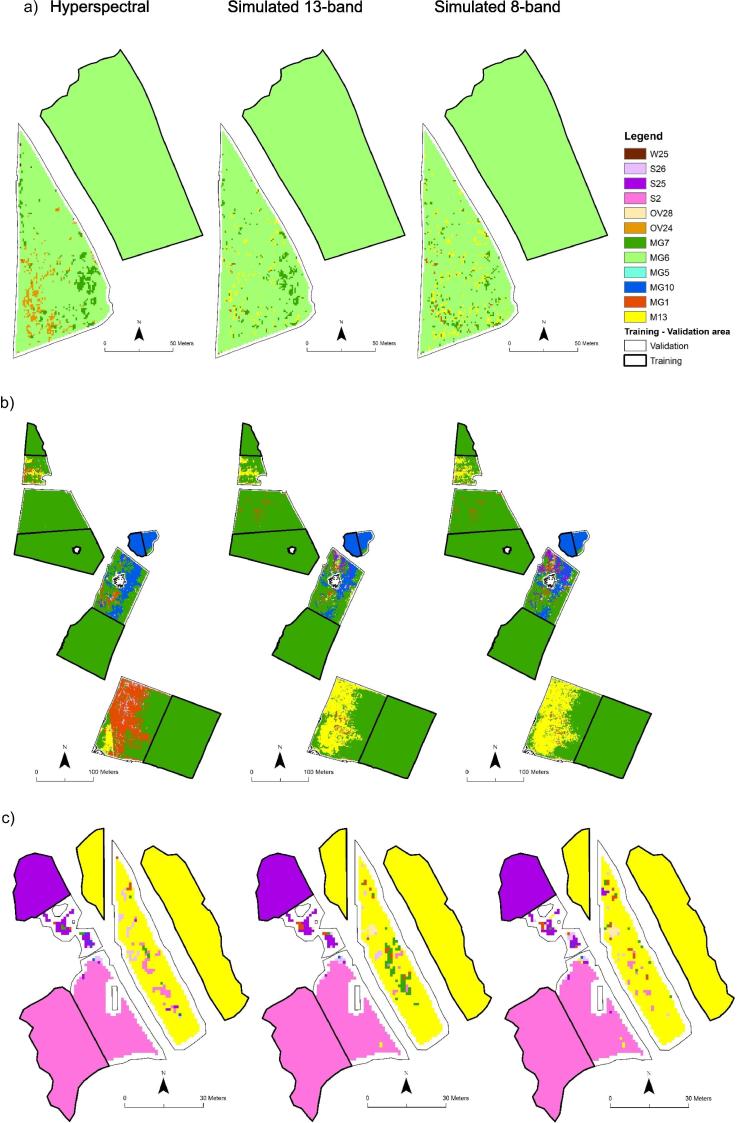
Some example patches showing NVC communities used to train the random forest classifier (thick black line) and predicted NVC communities (thin black line) using hyperspectral (left), simulated 13-band (middle) and simulated 8-band data (right) for patches of NVC communities MG6 (a), MG7 and MG10 (b) and S2, S25 and M13 (c).

The ground-truth data per type was split geographically into two halves for the airborne data. One half was used to train the classifier and the other for validation (Fig. 3). Geographically separated validation data can produce lower accuracies than randomly sampled validation data which are spatially closer to training data, therefore presenting a tougher test (Bahn and McGill, 2013). In the validation data, pixels per category were randomly sampled up to the downsampling value, repeated five times.

The field spectroscopy data, which had a median number of spectra per category of 15 – 66 (Table 2), were deemed of insufficient size for splitting into a training and validation set. Instead, accuracies were calculated using the OOB accuracy of random forest.

##### 2.5.2.1 Accuracy measures

Overall accuracy (accuracy henceforth) was calculated because it is directly relevant for users and easy to interpret (Foody, 2002). Accuracy was calculated as:

$$\mathrm{Accuracy}=\frac{\mathrm{number}\;of\;correctly\;classified\;pixels}{\mathrm{total}\;number\;of\;pixels}$$

Kappa was also calculated as it is a widely used accuracy measure in remote sensing (Foody, 2002, Lillesand et al., 2008, Pontius and Millones, 2011). Kappa can take values between 0 (classification is no better than random) and 1 (perfect agreement with ground-truth data) and, in contrast to the accuracy measure, assesses the improvement over chance agreement (Foody, 2002, Lillesand et al., 2008, Pontius and Millones, 2011). The use of randomness as a baseline has been criticized as not useful for map production (Pontius and Millones, 2011). However, in this study we believe the kappa value provides useful information, as several thematic map resolutions were compared and the amount of chance agreement varies with the number of categories. Kappa is sensitive to sample size and large differences in category sizes (Fielding and Bell, 1997). However, differences in category sizes had already been reduced in the data and sample sizes were constant for thematic resolutions. Kappa was calculated with R package asbio (Aho, 2016) as:

$$Kappa=\left(N\sum_{i=1}^{r}x_{\mathrm{ii}}-\sum_{i=1}^{r}{(x}_{i+}*x_{+i}\right))/\left(N^{2}-\sum_{i=1}^{r}\left(x_{i+}*x_{+i}\right)\right)$$

where r = number of rows in the error matrix, x*_ii_* = number of observations on the error matrix diagonal, x*_i+_* = number of observations in row *i*, x_+1_ = number of observations in column *i*, N = total number of observations (Aho, 2016, Lillesand et al., 2008).

## 3 Results

### 3.1 Hyperspectral imagery and thematic resolution

**Fig. 4:**
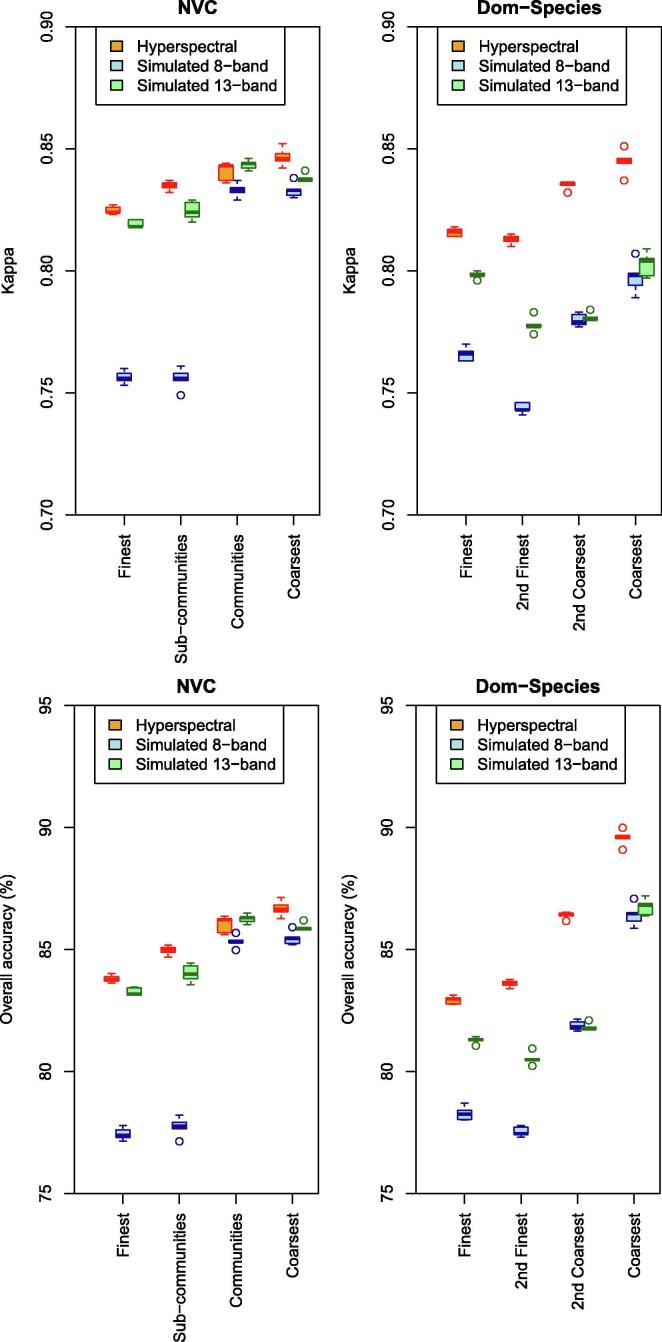
Prediction accuracies (top: Kappa, bottom: accuracy) per thematic resolution for vegetation grouped by the NVC (left) or by the dominating species (right) using hyperspectral data (orange), simulated 8-band (blue) and simulated 13-band data (green) for vegetation away from vegetation edges (category Wide). Boxplots are from five random selections of validation pixels. Boxes showing interquartile range and median (grey line); whiskers show the maximum of 1.5 * interquartile range. (For interpretation of the references to colour in this figure legend, the reader is referred to the web version of this article.)

Overall, kappa and accuracies increased with decreasing thematic resolution (Fig. 4, for confusion matrices, see Appendix N) when using hyperspectral imagery and when vegetation was away from edges. Even for the finest thematic resolution, kappa (Mean: 0.83 for NVC, 0.82 for Dom-Species) and accuracies (Mean: 84% for NVC, 83% for Dom-Species) were high. For the coarsest thematic resolution kappa (Mean: 0.85 for NVC, 0.84 for Dom-Species) and accuracies (Mean: 87% for NVC, 90% for Dom-Species) were higher, but the difference between thematic resolutions was moderate for kappa (Fig. 4).

### 3.2 Spectral resolution

Using hyperspectral airborne data usually achieved higher accuracies and kappa than using simulated data with lower spectral resolution when vegetation was away from edges (category Wide, Fig. 4, for confusion matrices see Appendix N). This was the case when vegetation was categorized by NVC (accuracy: 84–87%; kappa: 0.83–0.85; means per thematic resolution) or by the dominant plant species (Dom-Species; accuracy: 83–90%; kappa: 0.81–0.84). Using simulated 13-band data with the NVC produced usually only slightly lower, and sometimes even similar accuracies (83–86%) and kappa (0.82–0.84), while with Dom-Species accuracies (81–87%) and kappa (0.78–0.80) were clearly lower compared to hyperspectral data. The poorest results were produced by simulated 8-band data for the two finer thematic resolutions (NVC: accuracy: 77–78%, kappa: 0.76: Dom-Species: accuracy: 78%, kappa: 0.74–0.77). However, at the coarser thematic resolutions, simulated 8-band data produced results close to those produces by simulated 13-band and hyperspectral data for the NVC (accuracy: 85%, kappa: 0.83) (Fig. 4).

An example for NVC communities showed that the majority of predicted patches were clearly dominated by the correct vegetation community for hyperspectral, simulated 13-band and 8-band data (17, 17 and 16, respectively out of 29) or had a substantial proportion of the area covered by the correct predicted community (an additional eight for the hyperspectral and simulated 13-band data and nine for the simulated 8-band data, Fig. 3, Appendix O).

### 3.3 Vegetation classification system

Grouping vegetation by NVC usually produced higher kappa compared to Dom-Species when vegetation was away from edges with few exceptions (Fig. 4, for confusion matrices and plant species composition of Dom-Species categories, see Appendix N). Accuracies showed a similar trend, but the small number (four) of vegetation categories at the coarsest Dom-Species resolution led to very high accuracies (86–90%, Fig. 4).

### 3.4 Narrow objects

**Fig. 5:**
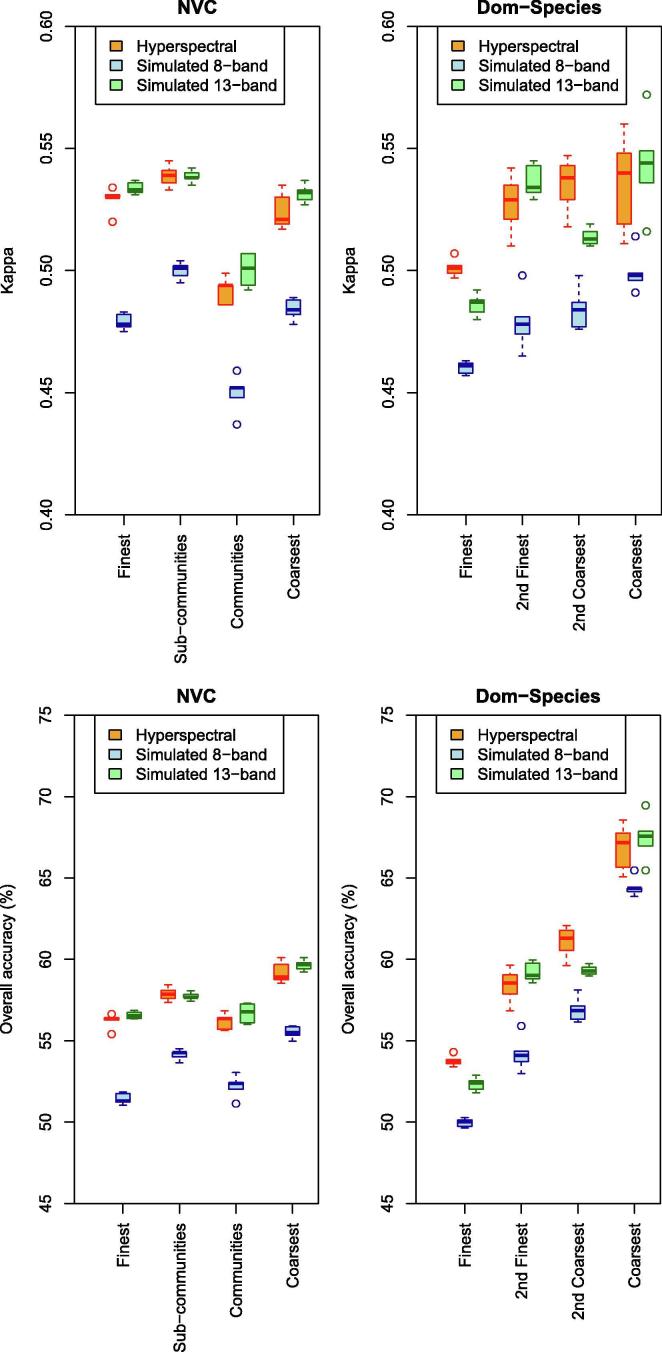
Prediction accuracies (top: Kappa, bottom: accuracy) per thematic resolution for vegetation grouped by the NVC (left) or by the dominating species (right) using hyperspectral data (orange), simulated 8-band (blue) and simulated 13-band data (green) for vegetation near vegetation edges (category Narrow). Boxplots are from five random selections of validation pixels. Boxes showing interquartile range and median (grey line); whiskers show the maximum of 1.5 * interquartile range. (For interpretation of the references to colour in this figure legend, the reader is referred to the web version of this article.)

When vegetation was located near edges, accuracies and kappa were overall poor (Fig. 5). Hyperspectral and simulated 13-band data produced higher accuracies and kappa compared to the simulated 8-band data. Even so, Kappa remained poor (NVC: 0.45–0.54; Dom-Species: 0.46–0.54; means of five repetitions per thematic resolution).

### 3.5 Acquisition month

In the between-month comparisons with spectroscopy data, higher accuracies were achieved in June and July (84–85%) than in May (75%) (Table 2, for confusion matrices and a characterisation of the vegetation categories, see Appendix P). However, two categories showed high confusion and were grouped in May and June, but not in July. In both August and September, due to weather conditions, data from only two categories could be recorded achieving accuracies of 94% in August and 97% in September (Table 2).

### 3.6 Important spectral covariates

**Fig. 6:**
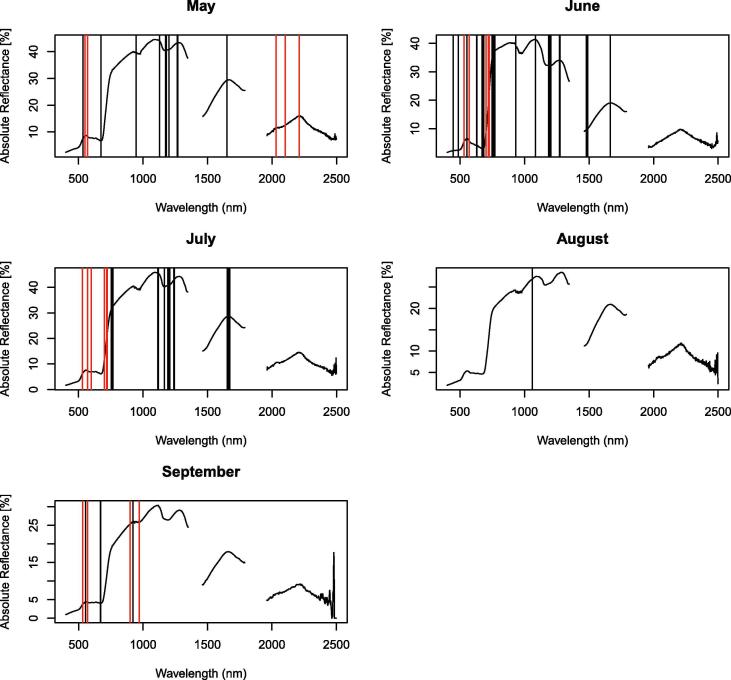
Selected bands in the classifications with field spectroscopy data. 1st derivative in black; bands that were part of other spectral covariates (see Table 3) in red. (For interpretation of the references to colour in this figure legend, the reader is referred to the web version of this article.)

Selected spectral covariates in the classifications with field spectroscopy data were from the visible, near-infrared and short-wave infrared part of the electromagnetic spectrum (Fig. 6, Table 3). Some spectral covariates were selected in several months (e.g. first derivative at 554 nm, 670 nm, 675 nm, 754 nm, 766 nm, 1193 nm, 1205 nm, 1270 nm, 1665 nm, PRI), however, others were selected only once or twice.

**Table 3:** Selected spectral covariates, other than first derivatives, in the classifications with field spectroscopy data. For selected first derivatives, see Fig. 6. For a description of the vegetation indices, see Appendix E.

Spectral covariate	May	June	June	July	September
Vegetation indices	ARI	PRI	PRI	PRI	PRI
	CAI		mARI		WBI
			NDVI		
			RGRI		
			NDLI		
			RVSI		
Minimum/maximum first derivative			1205–1285 nm	550–650 nm	
wavelength position in range					
Ratio of red-edge peaks		Peak 1 & 2	Peak 1 & 2	Peak 1 & 2	
		Peak 2 & 3	Peak 2 & 3	Peak 2 & 3	
			Peak 1 & 3		

## 4 Discussion

Spatial conservation planning to slow the rate of biodiversity loss benefits from detailed maps of where organisms occur (Elith and Leathwick, 2009). Remote sensing can enable such mapping over large areas (Kuenzer et al., 2014). However, better differentiation of vegetation is needed to improve the ability to use remote-sensing data for conservation (Sutherland et al., 2014). In this study, the influence on the thematic resolution and accuracy of vegetation classification of the spectral resolution of remotely sensed data, the month of data acquisition, the system used to categorize vegetation, and the narrowness of vegetation objects was evaluated. Hence, this study provides guidance for increasing the thematic resolution of remotely sensed vegetation maps. Specifically, hyperspectral data and two reduced spectral resolutions were evaluated for a ‘difficult to map’ habitat: grass-dominated farmland vegetation. Further, mapping using the popular NVC vegetation categories and categories based on the dominating plant species was evaluated.

### 4.1 Classification accuracies

The accuracies for classifying NVC at the community and sub-community level in this study, when vegetation was away from edges, were high compared to other studies. At the sub-community level, 85% of pixels were classified correctly (using hyperspectral data) versus 67% using 15-band Compact Airborne Spectrographic Imager (CASI) in a dune habitat (Shanmugam et al., 2003). At the community level, a classification accuracy of 86% in this study compares to 76% using CASI data in a dune habitat (Shanmugam et al., 2003) and 77% using Landsat TM and ancillary data in a peatland habitat (Reid and Quarmby, 2000). Very high accuracies (87–92%) were found using aerial multispectral imagery to classify NVC communities in the UK uplands, but only when ancillary data were included (Bradter et al., 2011). Imagery data alone produced low accuracies of 22–52% (Bradter et al., 2011). The classification accuracies in this study are expected to reflect some realism as the ground-truth data were widely distributed across a large area (minimum convex polygon around the ground-truth data: 48 km^2^), and therefore cover a variety of environmental conditions. However, as in many other studies it was not possible to more widely separate training and validation data spatially as data for rarer categories was sparse.

Very high thematic resolutions as in this study are rarely considered in remotely sensed vegetation classification, but are important for ecological applications. Despite working in a ‘difficult to map’ habitat (grass-dominated farmland vegetation), high accuracies were achieved even at the very high thematic resolutions of NVC sub-communities and finer. Critically, high accuracies were achieved without adding ancillary data (such as topography, soil type). Such ancillary helped to improve classification accuracies in several studies due to the associations between the distribution of vegetation types and environmental variables (Bradter et al., 2011, Dirnböck et al., 2003, Dobrowski et al., 2008, Sesnie et al., 2008). Anthropogenic activities can weaken or remove the associations between vegetation types and environmental variables (Dirnböck et al., 2003, Lees and Ritman, 1991). Such activities can be widespread and intense in arable landscapes, hence it is important that high classification accuracies can be achieved from spectral data alone.

### 4.2 Spectral resolution

Other studies were able to resolve vegetation with a high level of detail using hyperspectral data (Feilhauer and Schmidtlein, 2011, Harris et al., 2015, Mansour et al., 2012, Möckel et al., 2014, Schmidtlein et al., 2012, Schweiger et al., 2017). This is consistent with results in this study where for vegetation away from edges, the hyperspectral data produced high accuracies that were usually higher compared to the results from the simulated data with reduced spectral resolution. However, in this study the benefit from using hyperspectral data was small compared to the simulated 13-band data, for which bands are particularly suitable for vegetation mapping. The Sentinel-2 satellite mission, which was the basis for the simulated 13-band data in this study, has been launched comparatively recently (in 2015 and 2017) and the applicability of Sentinel-2 data for the mapping of grassland habitats has so far rarely been studied (Rapinel et al., 2019). Importantly therefore, this study demonstrates that the spatial resolution of hyperspectral imagery did not produce a considerable gain in either accuracy or thematic resolution compared to the spectral resolution of the Sentinel-2 mission for high thematic resolution mapping of grass-dominated farmland vegetation.

For the two coarsest NVC resolutions, there was little benefit from using hyperspectral data even compared to the simulated 8-band data. This is in contrast to a study where simulated multispectral data of several sensors, including Landsat 5 TM and Landsat 7 ETM+, resulted in poor discrimination of three coarser thematic resolution vegetation categories (Feilhauer et al., 2013). It is not surprising that studies using different vegetation lead to different results as some vegetation categories are more spectrally distinct than others. In this study a somewhat larger number of coarse vegetation categories (12 NVC communities in Wide) was used suggesting that the results in this study are not strongly influenced by large effects of a few highly spectrally similar or dissimilar categories.

This study was concerned with differences in vegetation classification accuracies caused by the spectral resolution of the hyperspectral, simulated 13-band and 8-band data. Consequently, the spatial resolution of the hyperspectral data had been retained in the simulated data. A caveat of this study is that due to the high spectral resolution of the hyperspectral data they originate from (Thenkabail et al., 2012), the simulated data may have a lower signal-to-noise ratio compared to recording the same 13 and eight bands at the same spatial resolution (1 m × 1 m) directly with an airborne platform. This may have influenced the accuracies and kappa produced. However, many of our comparisons are relative to each other and based on the same imagery. Therefore, this limitation may affect less the conclusions of our comparisons between NVC and Dom-Species and between thematic resolutions.

### 4.3 NVC versus grouping by dominating species

Classifications with the NVC usually resulted in higher accuracies compared to classifications based on the dominant plant species (Dom-Species). Intuitively, the Dom-Species approach may seem well suited to the nature of remote sensing data as the spectral reflectance of an image pixel tends to represent the dominant vegetation (Ustin and Gamon, 2010). However, Pottier et al. (2014) found that the abundance of plant species did not explain variation in spectral separability and suggested this was because remote sensing data not only reflect the plant species present, but also site conditions. Similarly, Schmidtlein et al. (2012) found that the inclusion of non-dominant species produced better vegetation models. NVC (sub)- communities are indicative of certain conditions (soil conditions, disturbance, etc.; Rodwell, 0000, Rodwell, 2006). Different habitat conditions can lead to differences in the chemical composition of plants or the structure of the plant canopy, even within the same species (Roberts et al., 2012). The good performance of using NVC in this study may therefore be because in addition to reflecting characteristics of dominant plant species, the NVC reflects differences in site conditions. However, a disadvantage of the NVC is that different surveyors may produce inconsistent results (Hearn et al., 2011), which can negatively affect remotely sensed vegetation mapping. It is therefore important to improve NVC survey methods (see Hearn et al., 2011 for suggestions) in order to provide consistent results. This study was not affected by such inconsistencies as NVC categories were assigned by a single person.

### 4.4 Vegetation in narrow objects

As expected, accuracies from airborne data in narrow objects were poor throughout. Image pixels from such areas frequently contain vegetation edges and can also be influenced by the scattering of reflectance from nearby objects.

In contrast, OOB accuracies from field spectroscopy data, which were mostly collected in narrow objects, were high. Spectroscopy data did not contain vegetation edges as these can be avoided during data collection. Spatially separate validation data were not available for the field spectroscopy data and accuracies therefore can likely not be achieved when classifying more distant samples (Bahn and McGill, 2013, Beale et al., 2008, Gavish et al., 2018). Nonetheless, the high separability of categories is promising.

Grass-dominated vegetation in narrow objects in farmland landscapes can be important for biodiversity (Gabriel et al., 2010). Due to the difficulties of remotely sensing vegetation in such objects, vegetation classification in narrow objects is frequently not attempted, thus risking that ecologically important habitats will be ignored in applications of remotely-sensed products (see O'Connell et al., 2015, Tansey et al., 2009 for methods to map narrow features in agricultural landscapes in broader habitat classes). Hand-held data collection as in this study is not feasible for larger mapping projects, but recording data with drones may be an alternative for very narrow objects. However, although this study suggests that small pixel sizes could help to map vegetation in narrow objects, noise can increase with decreasing pixel size, which is an important consideration (Lillesand et al., 2008, Rocchini et al., 2013). Imagery with a pixel size that is much smaller than the objects of interest may result in lower accuracies than for imagery with larger pixel sizes. For example, resampling to a larger pixel size (5 m) improved mapping accuracies for NVC categories in larger objects compared to the 0.25 m pixel size (Bradter et al., 2011). Alternatively, for mixed pixels spectral unmixing (e.g. Landmann et al., 2015) can be used or fuzzy-set theory, which produces a soft classification, in which several categories can be associated with a pixel (Lu and Weng, 2007, Rocchini et al., 2013). Such techniques are alternatives to the hard classification used in this study and promising for mapping vegetation in narrow objects.

### 4.5 Time of imagery acquisition

With spectroscopy data, the highest discrimination was achieved in July, the period when vegetation was fully developed. This is consistent with other studies that also found that categories could best be distinguished when vegetation was fully developed (Belluco et al., 2006, Feilhauer and Schmidtlein, 2011, Rapinel et al., 2019). However, some studies found that other dates in the vegetation periods were also good (Cole et al., 2014) suggesting that it depends on the vegetation under consideration. Good results were also achieved in June, and to a lesser extent in May in this study. The period after full vegetation development could not be as fully assessed due to a lack of data.

### 4.6 Important spectral predictors for vegetation discrimination

The selected vegetation indices, red-edge peak ratios and minimum/maximum first derivative wavelength positions are known to be associated with chlorophyll absorption (red-edge peak ratios and minimum first derivative wavelength position in the range 550 – 560 nm, Boochs et al., 1990, Pu et al., 2004), light use efficiency (PRI, Roberts et al., 2012), anthocyanins (ARI, mARI, RGRI, Roberts et al., 2012), cellulose and lignin absorption (minimum first derivative wavelength position in the range 1205–1285 nm, CAI and NDLI, Pu et al., 2004, Roberts et al., 2012), vegetation structure (NDVI, Roberts et al., 2012), vegetation structure and water content (WBI, Roberts et al., 2012) and vegetation stress (RVSI, Roberts et al., 2012). The first derivative in the visible and red-edge part of the electromagnetic spectrum, which were among our selected spectral covariates, have been linked to leaf pigment content, but also to canopy structural properties, such as leaf area index and variation in leaf angles (Kattenborn et al., 2018). The models also selected first derivatives in the near-infrared and short-wave infrared regions (see Fig. 1 for wavelength ranges), which have been linked to canopy structure and water content/dry matter content (Kattenborn et al., 2018). The selected spectral covariates therefore represented differences in the chemical composition of plant species, such as pigment, cellulose and lignin content. They also represent differences in the structure of plant tissue and the plant canopy. For example, variation in leaf angle contributes to differentiation between graminoids and forbs (Kattenborn et al., 2018) and could therefore contribute to differentiate vegetation communities, which have different proportions of forbs.

A reason for the frequent selection of bands in the near infrared (NIR) and shortwave infrared (SWIR) compared to the visible range could also be that they have a higher power to discriminate between vegetation categories. They cover a larger range of reflectance values (larger amplitude) compared to the visible range (see Fig. 1) and may therefore have more contrasting power to differentiate between categories.

## 5 Conclusions

The results of this study suggest that for high thematic resolutions, categorizing vegetation by the NVC can achieve higher accuracies than categorizing vegetation by the dominating plant species. Hyperspectral data achieved highest accuracies, but may not always be worth the cost as simulated 13-band data, with bands that were particularly suitable for vegetation studies, achieved accuracies that were only slightly lower. The study area posed several challenges, such as grass-dominated habitats, which are difficult to assess by remote sensing (Sutherland et al., 2014), many narrow objects and a relative rarity of the objects of interest within a matrix of arable fields making ground-data collection time consuming. Despite these challenges, high accuracies were achieved, and at a high thematic and spatial resolution that is rarely attempted. Vegetation classifications with high accuracies and with high thematic resolution can benefit a range of conservation applications, for example monitoring and reporting obligations (Stenzel et al., 2017), predictive mapping of animal species (Fletcher et al., 2016) and conservation planning (Elith and Leathwick, 2009).
