# Supplementary material for: Classifying grass-dominated habitats from remotely sensed data: The influence of spectral resolution, acquisition time and the vegetation classification system on accuracy and thematic resolution
Source: Sci Total Environ. 2020 Apr 1;711:134584. doi: 10.1016/j.scitotenv.2019.134584 (PMC7014585; doi:10.1016/j.scitotenv.2019.134584)
Supplement: Supplementary Data 1 [file mmc1.docx]

Appendix A for

Ute Bradter, Jerome O'Connell, William E. Kunin, Caroline W.H. Boffey, Richard J. Ellis, Tim G. Benton, Classifying grass-dominated habitats from remotely sensed data: the influence of spectral resolution, acquisition time and the vegetation classification system on accuracy and thematic resolution.

## Appendix A: Bands centres and bandwidths of the simulated data

8-band data

Band 1 (Coastal/Aerosol): 443 nm, bandwidth = 16 nm

Band 2 (Blue): 516 nm, bandwidth = 8 nm

Band 3 (Green): 561.5 nm, bandwidth = 57 nm

Band 4 (Red): 654.5 nm, bandwidth = 37 nm

Band 5 (Near infrared): 865 nm, bandwidth = 28 nm

Band 6 (Shortwave infrared 1): 1608.5 nm, bandwidth = 85 nm

Band 7 (Shortwave infrared 2): 2200.5 nm, bandwidth = 187 nm

Band 9 (Cirrus): 1373.5 nm, bandwidth = 21 nm

13-band data

Band 1 (Coastal aerosol): 442.3 nm, bandwidth = 45

Band 2 (Blue): 492.1 nm, bandwidth = 98

Band 3 (Green): 559 nm, bandwidth = 46

Band 4 (Red): 665 nm, bandwidth = 39

Band 5 (Vegetation red edge): 703.8 nm, bandwidth = 20

Band 6 (Vegetation red edge): 739.1 nm, bandwidth = 18

Band 7 (Vegetation red edge): 779.7 nm, bandwidth = 28

Band 8 (NIR): 833, bandwidth = 133

Band 8a (NIR): 864 nm, bandwidth = 32

Band 9 (Narrow NIR): 943.2 nm, bandwidth = 27

Band 10 (Water vapour): 1376.9 nm, bandwidth = 76

Band 11 (SWIR - Cirrus): 1610.4 nm, bandwidth = 141

Band 12 (SWIR): 2185.7 nm, bandwidth = 238
