# Supplementary material for: Classifying grass-dominated habitats from remotely sensed data: The influence of spectral resolution, acquisition time and the vegetation classification system on accuracy and thematic resolution
Source: Sci Total Environ. 2020 Apr 1;711:134584. doi: 10.1016/j.scitotenv.2019.134584 (PMC7014585; doi:10.1016/j.scitotenv.2019.134584)
Supplement: Supplementary Data 2 [file mmc2.docx]

Appendices D-M for:

Ute Bradter, Jerome O'Connell, William E. Kunin, Caroline W.H. Boffey, Richard J. Ellis, Tim G. Benton, Classifying grass-dominated habitats from remotely sensed data: the influence of spectral resolution, acquisition time and the vegetation classification system on accuracy and thematic resolution.

## Appendix D: Calculation and smoothing of first derivative

The first derivative of each spectrum outside of water absorption features (1350-1460 nm, 1790-1960 nm Robinson and MacArthur, 2011) was calculated and the derivative was smoothed using Savitzky-Golay filtering.

### Field spectroscopy data

The spectroradiometers recorded a single spectrum as an average of repeat scans, which reduces random noise. Noise is amplified in the calculation of derivatives (Ruffin et al., 2008; Tsai and Philpot, 1998), therefore we assessed if further smoothing improved results.

For data collected with the SVC HR-1024i the effect of different smoothing levels on classification accuracy was assessed: For each month random forest models were build using different smoothing levels. Models were repeated 50 times per smoothing level (as classification results can vary slightly between repeats due to the randomness in the algorithm) and the standard deviation of the overall error (OOB error) was added to the model producing the lowest averaged OOB error. The smoothing level resulting in an OOB error less than this was selected following a selection principle outlined by Genuer et al. (2010).

First, the effect of the smoothing level of the first derivative on classification accuracy was assessed. Only the values for the first derivative were used as predictors and polynomial degrees of 2-5 and window sizes up to 15 were compared. For each month, a polynomial degree of two produced the highest classification accuracies, with a window size (apart from June) of <=7.

Then, the effect of the smoothing level on other predictor groups (red-edge peaks and their ratios, minimum position and reflectance, the maximum and minimum slope positions and their first derivative values) was assessed. Comparisons were restricted to polynomial degrees of two and window sizes up to seven. For the minimum wavelength position and reflectance before the red-edge, additionally no smoothing was considered, as this predictor is calculated from the reflectance values, not the first derivative.

**Table D.1: window sizes resulting in the highest classification accuracies per predictor group and month for field spectroscopy data**

May June July August

First derivative 7 13 7 3

Red edge peaks & ratios 5 5 7 3

Min position and reflectance 0 7 7 0

Min and max slope positions and derivative 5 5 5 5

The differences in classification accuracy were comparatively small between the most commonly selected window sizes (five and seven, Table B.1). Therefore, a window size of five was chosen, the median value (Table B.1). The same window size and polynomial degree was also applied to the smaller dataset from the ASD Field Spec Pro.

### Airborne data

The airborne data were smoothed using a window size of 11 and a polynomial degree of 2. This smoothing level was selected following a similar evaluation as outlined for the spectroscopy data with a subset of high quality data (not including vegetation edges, etc.). Window sizes up to 15 with a polynomial degree of 2 were considered. Additionally, we visually inspected the effect of window size on the first derivative in the red-edge region and in the region around the minimum position before the red-edge, which confirmed that a window size of 11 produced good results.

### References

Genuer R, Poggi J-M, Tuleau-Malot C. Variable selection using random forests. Pattern Recognition Letters 2010; 31: 2225-2236.

Robinson I, MacArthur A. The Field Spectroscopy Facility Post Processing Toolbox User Guide: Post processing spectral data in MATLAB., Downloaded 06. Aug 2014 from www.fsf.nerc.ac.uk, 2011.

Ruffin C, King RL, Younan NH. A combined derivative spectroscopy and Savitzky-Golay filtering method for the analysis of hyperspectral data. GIScience & Remote Sensing 2008; 45: 1-15.

Tsai F, Philpot W. Derivative analysis of hyperspectral data. Remote Sensing of Environment 1998; 66: 41-51.

## Appendix E: Hyperspectral vegetation indices

The following vegetation indices suggested by Roberts et al. (2012) were used. If these authors supplied broad bands, bands suggested by the source in brackets were used. ENVI refers to the following source: <http://www.exelisvis.com/Learn/WhitepapersDetail/TabId/802/ArtMID/2627/ArticleID/13742/Vegetation-Analysis-Using-Vegetation-Indices-in-ENVI.aspx>, accessed 23^rd^ December 2014.

Vegetation indices related to vegetation structure:

NDVI: (750nm - 705nm) / (750nm + 705nm) (ENVI)

mSR: (750nm - 445nm) / (705nm - 445nm) (ENVI)

EVI: 2.5 * (829nm – 682nm) / (829nm + 6 * 682nm - 7.5 * 445nm + 1) (Adam et al., 2014)

ARVI: (865nm - (660nm - 1*(470nm – 660nm))) / (865nm + (660nm - 1 * (470nm – 660nm))) (Kaufman and Tanré, 1992)

SAVI: (1 + L) (800nm – 670nm) / (800nm + 670nm + L) (Haboudane et al., 2004) using L = 0.5 (Huete, 1988)

IDL_DGVI: $\sum_{\lambda626 nm}^{\lambda795 nm} |R^{'}\left( \lambda i \right)- R^{'}\left( \lambda626nm \right)|\Delta\lambda i$

VARI: (550nm – 670nm) (550nm + 670nm – 480nm) (Gitelson et al., 2002)

VIgreen; (550nm – 670nm) (550nm + 670nm) (Gitelson et al., 2002)

Vegetation indices related to vegetation structure and water:

NDWI: (857nm - 1241nm) / (857nm + 1241nm)

WBI: 900nm / 970 nm

Vegetation indices related to pigments:

SIPI: (800nm - 445nm) / (800nm - 680nm)

PSSR: (800nm / 675nm); (800nm / 650nm)

PSND: (800nm - 675nm) / (800nm + 675nm)

PSRI: (680nm - 500nm) / 750nm

Vegetation indices related to pigments and light use efficiency

SIPI: (800nm - 445nm) / (800nm - 680nm)

Vegetation indices related to chlorophyll:

CARI: (700nm - 670nm) - 0.2 * (700nm - 550nm)

MCARI: [(700nm - 670nm) - 0.2 * (700nm - 550nm)] * (700nm / 670nm)

CIRedEdge: (780nm / 700nm) -1 (Gitelson et al., 2006)

Vegetation indices related to anthocyanins:

ARI: (1 / 550nm) - (1 / 700nm) (ENVI)

mARI: 800 [(1 / 550nm) - (1 / 700nm)] (ENVI)

RGRI: mean of 600-699nm / mean of 500-599nm (ENVI)

ACI: 530nm / 940nm (van den Berg and Perkins, 2005)

Vegetation indices related to carotenoids:

CRI1: (1 / 510nm) - (1 / 550nm)

CRI2: (1 / 510nm) - (1 / 700nm)

Vegetation indices related to water:

NDII: (819nm – 1649nm) / (819nm + 1649nm) (ENVI)

Vegetation indices related to water and stress:

MSI: 1599nm / 819nm (ENVI)

Vegetation indices related to lignin, cellulose, residues

CAI: 100 * [0.5 * (2031nm + 2211nm) - 2101nm]

NDLI: [log (1 / 1754nm) - log(1 / 1680nm)] / [log(1 / 1754nm) + log(1 / 1680nm)

Vegetation indices related to nitrogen:

NDNI: [log (1 / 1510nm) - log(1 / 1680nm)] / [log(1 / 1510nm) + log(1 / 1680nm)

Vegetation indices related to light use efficiency:

PRI: (531nm - 570nm) / (531nm + 570nm)

Vegetation indices related to stress:

RVSI: ((714nm + 752nm) / 2) - 733nm

### References

Adam E, Mutanga O, Abdel-Rahman EM, Ismail R. Estimating standing biomass in papyrus (*Cyperus papyrus* L.) swamp: exploratory of *in situ* hyperspectral indices and random forest regression. International Journal of Remote Sensing 2014; 35: 693-714.

Gitelson AA, Kaufman YJ, Stark R, Rundquist D. Novel algorithms for remote estimation of vegetation fraction. Remote Sensing of Environment 2002; 80: 76-87.

Gitelson AA, Keydan GP, Merzlyak MN. Three-band model for noninvasive estimation of chlorophyll, carotenoids, and anthocyanin contents in higher plant leaves. Geophysical research letters 2006; 33.

Haboudane D, Miller JR, Pattey E, Zarco-Tejada PJ, Strachan IB. Hyperspectral vegetation indices and novel algorithms for predicting green LAI of crop canopies: modeling and validation in the context of precision agriculture. Remote Sensing of Environment 2004; 90: 337-352.

Huete A. A soil-adjusted vegetation index (SAVI). Remote Sensing of Environment 1988; 25: 295-309.

Kaufman YJ, Tanré D. Atmospherically resistant vegetation index (ARVI) for EOS-MODIS. Ieee Transactions On Geoscience And Remote Sensing 1992; 30: 261-270.

Roberts DA, Roth KL, Perroy RL. Hyperspectral vegetation indices. In: Thenkabail PS, Lyon JG, Huete A, editors. Hyperspectral remote sensing of vegetation. CRC Press, Boca Raton, 2012, pp. 309-327.

van den Berg AK, Perkins TD. Nondestructive estimation of anthocyanin content in autumn sugar maple leaves. Horticultural Science 2005; 40: 685-686.

## Appendix F: Vegetation indices used for the simulated data

8-band data:

We used the vegetation indices described in https://landsat.usgs.gov/sites/default/files/documents/si_product_guide.pdf (accessed 7th October 2018)/

NDVI: (Band 5 – Band 4) / (Band 5 + Band 4)

EVI: 2.5 * ((Band 5 – Band 4) / (Band 5 + 6 * Band 4 – 7.5 * Band 2 + 1))

SAVI: ((Band 5 – Band 4) / (Band 5 + Band 4 + 0.5)) * (1.5)

MSAVI: (2 * Band 5 + 1 – sqrt ((2 * Band 5 + 1)^2^ – 8 * (Band 5 – Band 4))) / 2

NDMI: (Band 5 – Band 6) / (Band 5 + Band 6)

NBR: (Band 5 – Band 7) / (Band 5 + Band 7)

NBR2: (Band 6 – Band 7) / (Band 6 + Band 7)

13-band data:

We used the vegetation indices described in: https://www.sentinel-hub.com/develop/documentation/eo_products/Sentinel2EOproducts (accessed 16 September 2018).

SIPI1: (Band8 - Band1) / (Band8 - Band4)

PSSR: Band8 / Band4

NDVI: (Band8 - Band4) / (Band8 + Band4)

NBRRaw: (Band8 - Band12) / (Band8 + Band12)

MCARI: ((Band5/100 - Band4/100) - 0.2 * (Band5/100 - Band3/100)) * (Band5/100 / Band4/100)

EVI: 2.5 * (Band8/100 - Band4/100) / ((Band8/100 + 6 * Band4/100 - 7.5 * Band2/100) + 1)

CHLRedEdge: (Band7 / Band5) ^ -1

ARI1: 1 / Band3 - 1 / Band5

SAVI: (1 + 0.428) * ((Band8/100 - Band4/100) / (Band8/100 + Band4/100 + 0.428))

NDWI: (Band3 - Band8) / (Band3 + Band8)

NDII: (Band8 - Band11) / (Band8 + Band11)

MSI: Band11 / Band8

GNDVI: (Band8 - Band3) / (Band8 + Band3)

EVI2: 2.4 * (Band8/100 - Band4/100) / (Band8/100 + Band4/100 + 1)

ARVI: (Band9/100 - Band4/100 - 0.106 * (Band4/100 - Band2/100)) / (Band9/100 + Band4/100 - 0.106 * (Band4/100 - Band2/100))

## Appendix G: NVC communities in the airborne ground-truth data.

Mires

M13 *Schoenus nigricans – Juncus subnodulosus* mire

Mesotrophic grasslands

MG1 *Arrhenatherum elatius* grassland

MG5 *Cynosurus cristatus – Centaurea nigra* grassland

MG6 *Lolium perenne – Cynosurus cristatus* grassland

MG7 *Lolium perenne* leys and related grasslands

MG10 *Holcus lanatus – Juncus effusus* rush-pasture

Vegetation of open habitats

OV23 *Lolium perenne – Dactylis glomerata* community

OV24 *Urtica dioica – Galium aparine* community

OV25 *Urtica dioica – Cirsium arvense* community

OV26  *Epilobium hirsutum* community

OV28 *Agrostis stolonifera – Ranunculus repens* community

Swamps and tall-herb fens

S2 *Cladium mariscus* swamp

S12 *Typha latifolia* swamp

S14 *Sparganium erectum* swamp

S25 *Phragmites australis – Eupatorium cannabinum* tall-herb fen

S26 *Phragmites australis – Urtica dioica* tall-herb fen

Woodlands and scrub

W24 *Rubus fruticosus – Holcus lanatus* underscrub

W25 *Rubus fruticosus – Pteridium aquilinum* underscrub

## Appendix H: Climate in the study area


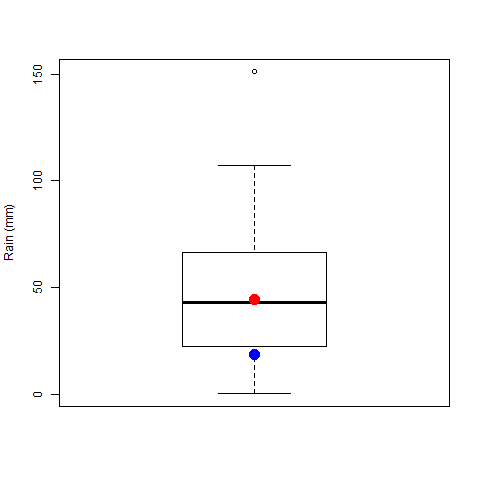


## Fig H 1a

Precipitation in June in Cambridge, near the study area in the years prior to the study (1961 – 2013). The box shows the interquartile range and median; whiskers show the maximum of 1.5 * interquartile range. The red dot represents rainfall in June 2014 when the aerial hyperspectral data were recorded. The blue dot represents rainfall in June 2015 when the vegetation was recorded. Weather data downloaded from [https://www.metoffice.gov.uk/public/weather/climate-historic/#?tab=climateHistoric](https://www.metoffice.gov.uk/public/weather/climate-historic/#?tab=climateHistoric) on 1^st^ February 2019.


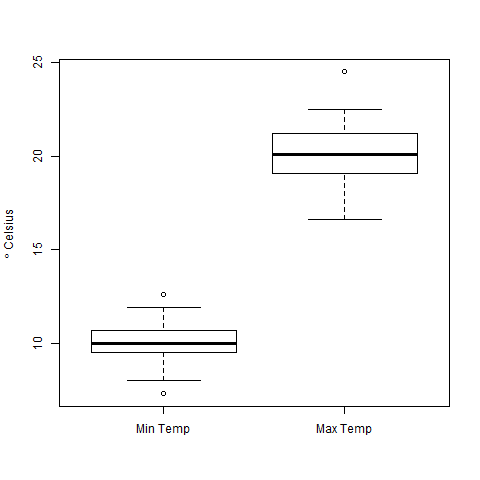


## Fig H 1b

Minimum and maximum temperature in June in Cambridge near the study area in the years prior to the study (1961 – 2013). The box shows the interquartile range and median; whiskers show the maximum of 1.5 * interquartile range. Weather data downloaded from [https://www.metoffice.gov.uk/public/weather/climate-historic/#?tab=climateHistoric](https://www.metoffice.gov.uk/public/weather/climate-historic/#?tab=climateHistoric) on 1^st^ February 2019.

## Appendix I: Vegetation mapping

Larger patches represented on the Ordnance Survey MasterMap (downloaded from the EDINA Digimap OS service; <http://edina.ac.uk/digimap>. © Crown Copyright/database right 2012. An Ordnance Survey/EDINA supplied service) were mapped from the MasterMap. Smaller patches or those not represented on the topographic map were mapped with a Navcom SF-3040 GPS (Navcom, Torrance, USA) with horizontal accuracy usually < 10-15 cm. We also removed areas with trees by calculating the difference between a DTM (digital terrain model) and a DSM (digital surface model) and removing all sections where this difference was greater than 1.8 m. The 1m resolution DTM and DSM were produced from LiDAR data recorded by ARSF simultaneously with the airborne hyperspectral data, with average point density of 5.1 per m^2^.

## Appendix J: Clustering of vegetation for Dom-Species

Following Shi and Horvarth (2006) we calculated a dissimilarity measure between all vegetation samples with which we categorized the samples using partitioning around medoids (PAM) clustering. This requires a tuning parameter, the number of clusters. We carried out PAM using every integer value between 3 and 40. We then assessed how often sample plots from the same type were assigned to more than one category. We interpreted a low number as categories resembling more closely the distinctions made by surveyors in the field. Therefore, we created four thematic resolutions by choosing the number of clusters resulting in the fewest types being split.

### Reference

Shi T, Horvarth S. Unsupervised learning with random forest predictors. Journal of Computational and Graphical Statistics 2006; 15: 118-138.

## Appendix K: Precipitation in the months field spectroscopy measurements were collected


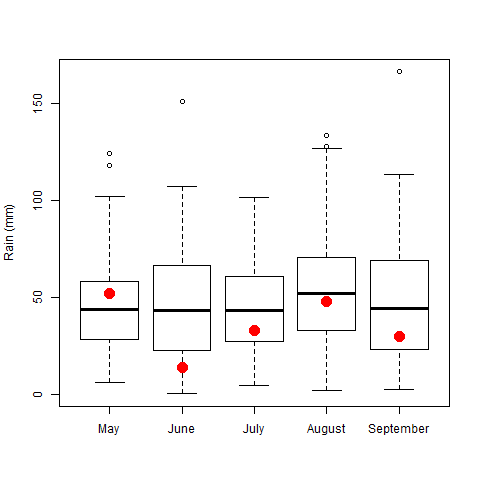


## Fig K 1

Precipitation in Cambridge, near the study area in the years prior to the study (1961 – 2013). The box shows the interquartile range and median; whiskers show the maximum of 1.5 * interquartile range. The red dots represents rainfall in the months field spectroscopy measurements were recorded. Weather data downloaded from [https://www.metoffice.gov.uk/public/weather/climate-historic/#?tab=climateHistoric](https://www.metoffice.gov.uk/public/weather/climate-historic/#?tab=climateHistoric) on 1^st^ February 2019.

## Appendix L: Number of training pixels in classifications with airborne imagery after downsampling

**Classification Thematic resolution No of training pixels**

NVC and Wide Finest 11577

NVC and Wide Sub-communities 9816

NVC and Wide Communities 6815

NVC and Wide Coarsest 6815

Dom-Species and Wide Finest 18628

Dom-Species and Wide 2^nd^ Finest 11109

Dom-Species and Wide 2^nd^ Coarsest 8270

Dom-Species and Wide Coarsest 4544

NVC and Narrow Finest 3660

NVC and Narrow Sub-communities 2979

NVC and Narrow Communities 1930

NVC and Narrow Coarsest 1930

Dom-Species and Narrow Finest 3289

Dom-Species and Narrow 2^nd^ Finest 2386

Dom-Species and Narrow 2^nd^ Coarsest 1724

Dom-Species and Narrow Coarsest 1002

## Appendix M: Variable selection with random forest

The variable selection proposed by Genuer et al. (2010) consists of the following steps:

1) The permutation importance for 50 repetitions of a model with all variables is calculated using the tuning parameters values ntree = 2000 and mtry = p / 2 (p: number of covariates).

2) The predictors are ranked by their mean permutation importance.

3) The predictors with very low importance (noise variables) are omitted to save computing time in the next step, by fitting a CART model to standard deviations of the ranked permutation importance measures. Only variables with a mean importance greater than the minimum prediction value from the CART model are kept.

4) Average OOB error of 50 repetitions in a forward selection is calculated (for the model with the highest ranked predictor only, then for the model with the two highest ranked predictors, etc) using default ntree and mtry.

5) The model with the lowest mean OOB error of the 50 repetitions is selected and the OOB error is augmented by its standard deviation.

6) The model with the lowest number of predictors with an OOB error less than this is selected as the final set of variables.

### Reference

Genuer R, Poggi J-M, Tuleau-Malot C. Variable selection using random forests. Pattern Recognition Letters 2010; 31: 2225-2236.
