# Supplementary material for: Classifying grass-dominated habitats from remotely sensed data: The influence of spectral resolution, acquisition time and the vegetation classification system on accuracy and thematic resolution
Source: Sci Total Environ. 2020 Apr 1;711:134584. doi: 10.1016/j.scitotenv.2019.134584 (PMC7014585; doi:10.1016/j.scitotenv.2019.134584)
Supplement: Supplementary Data 4 [file mmc4.docx]

## Appendix O: Classification plots for NVC communities

Hyperspectral Simulated 13-band Simulated 8-band


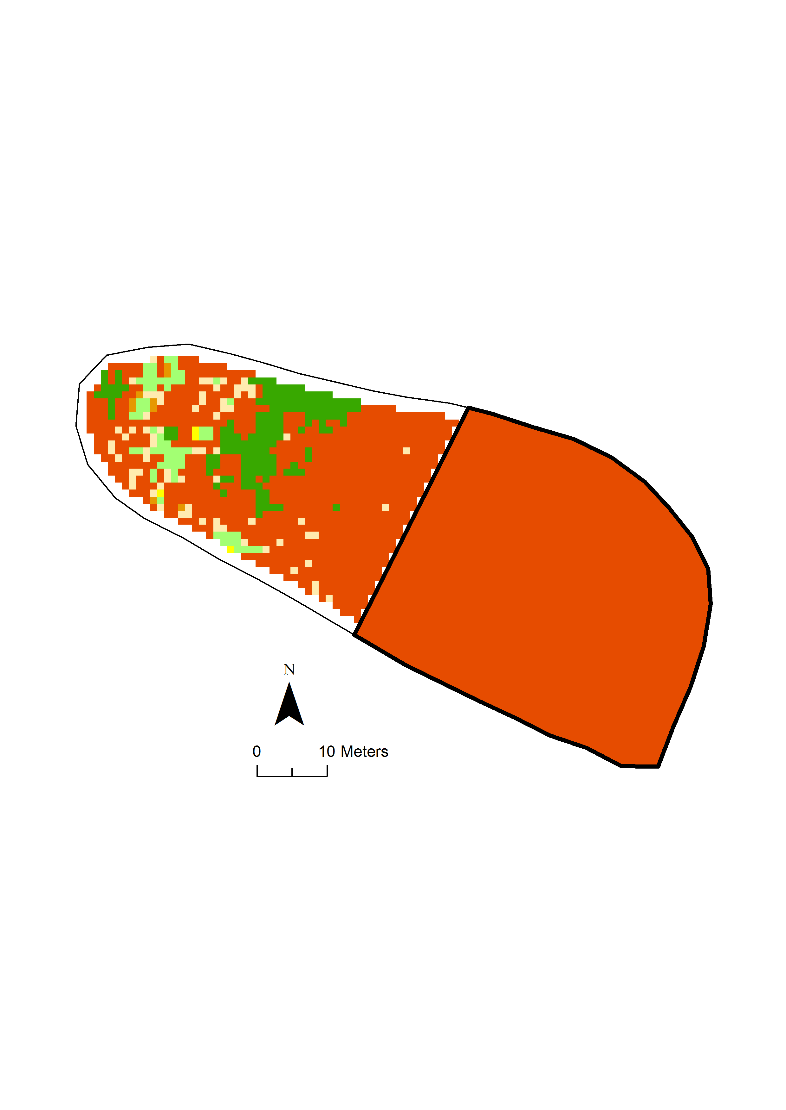

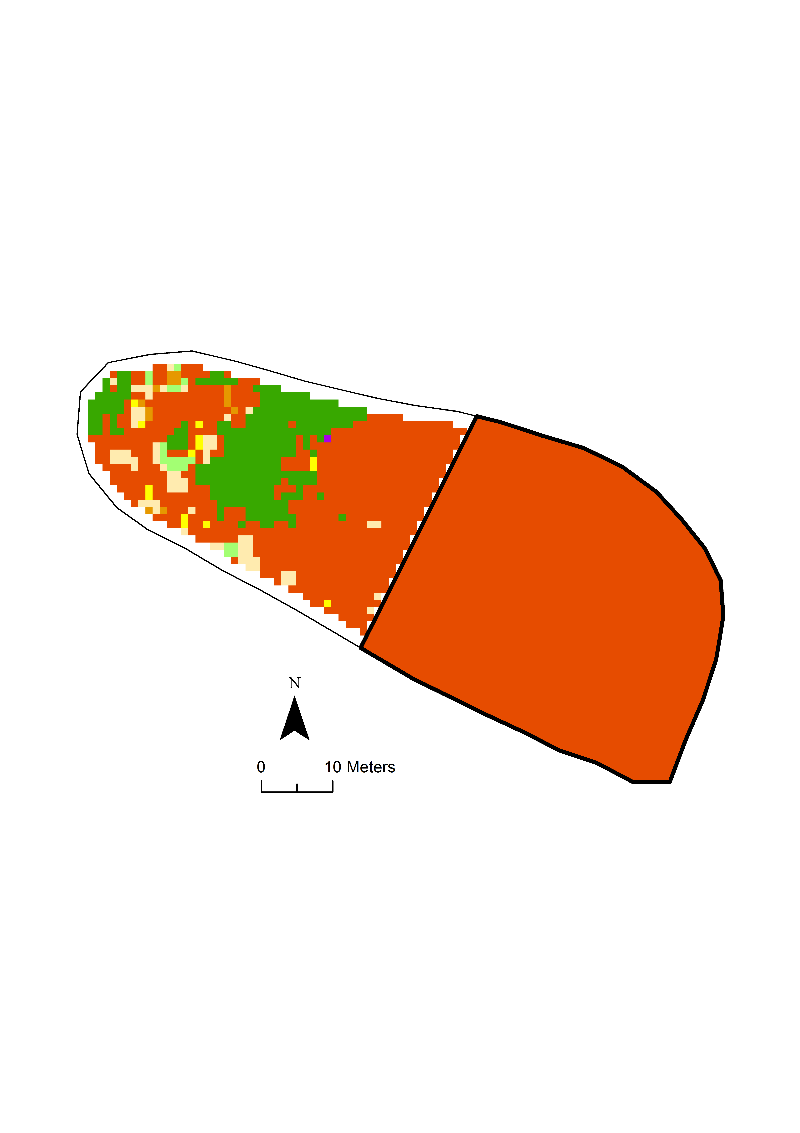

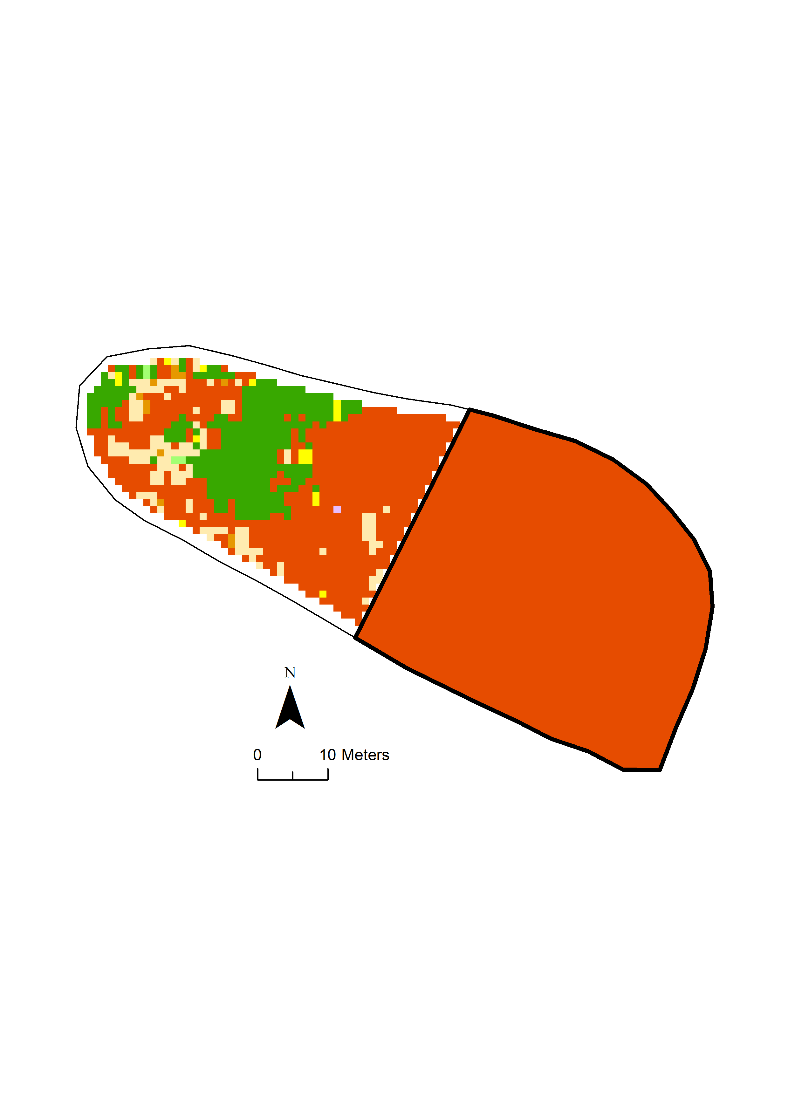

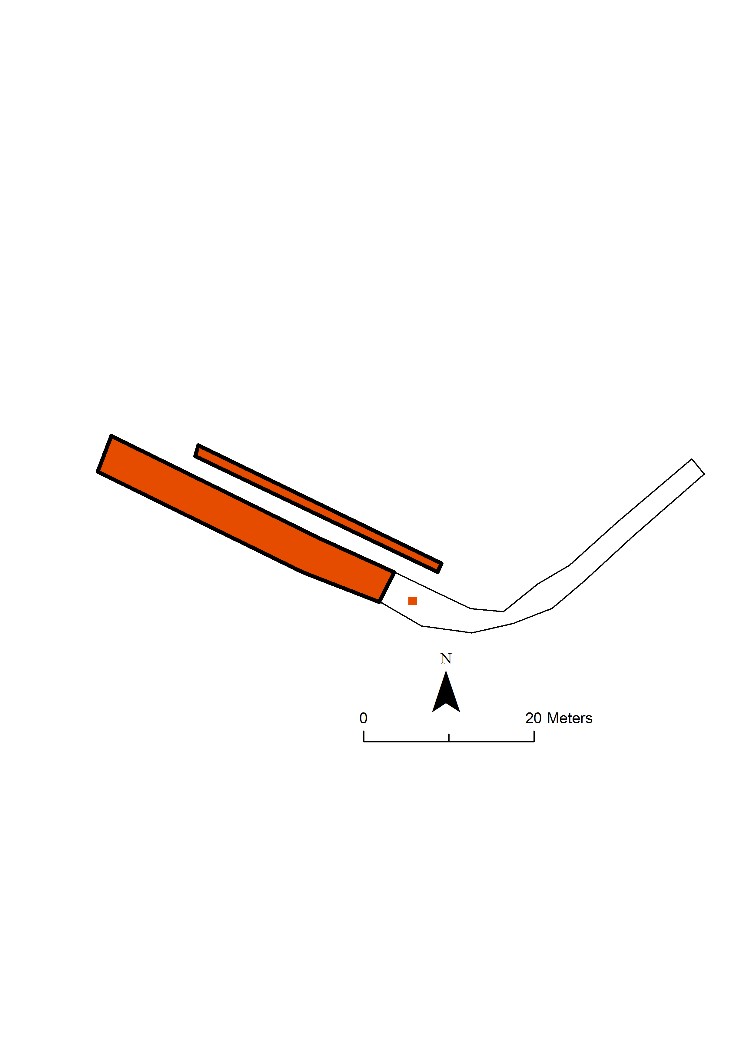

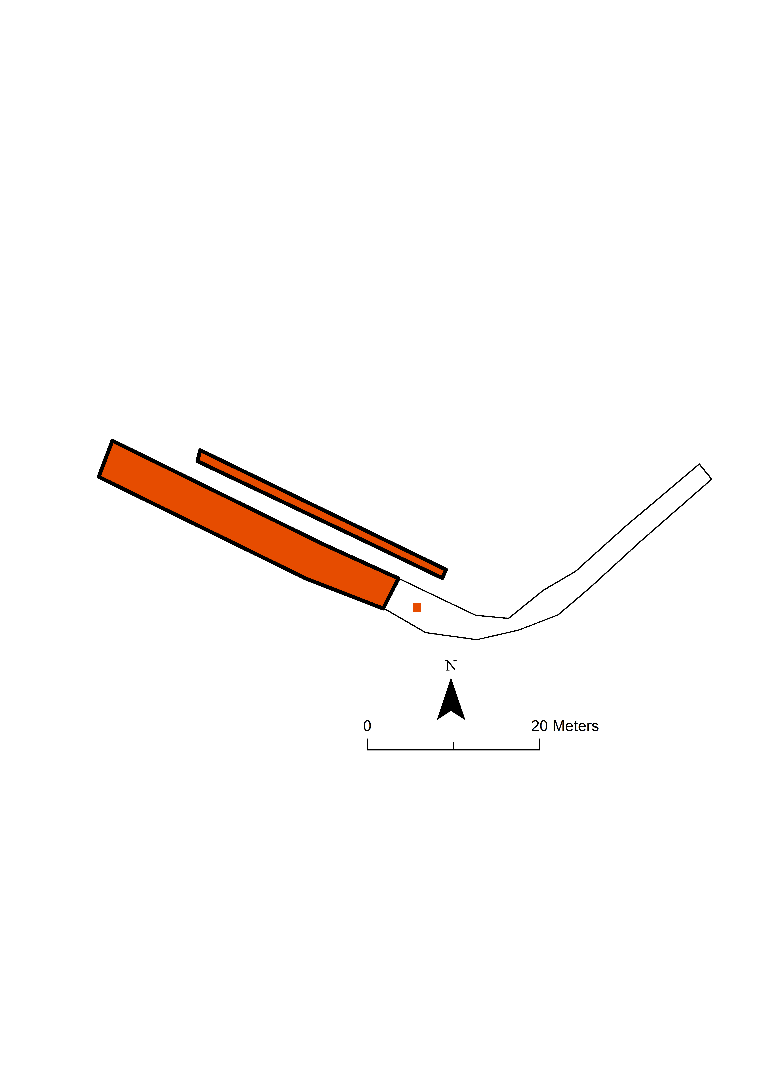

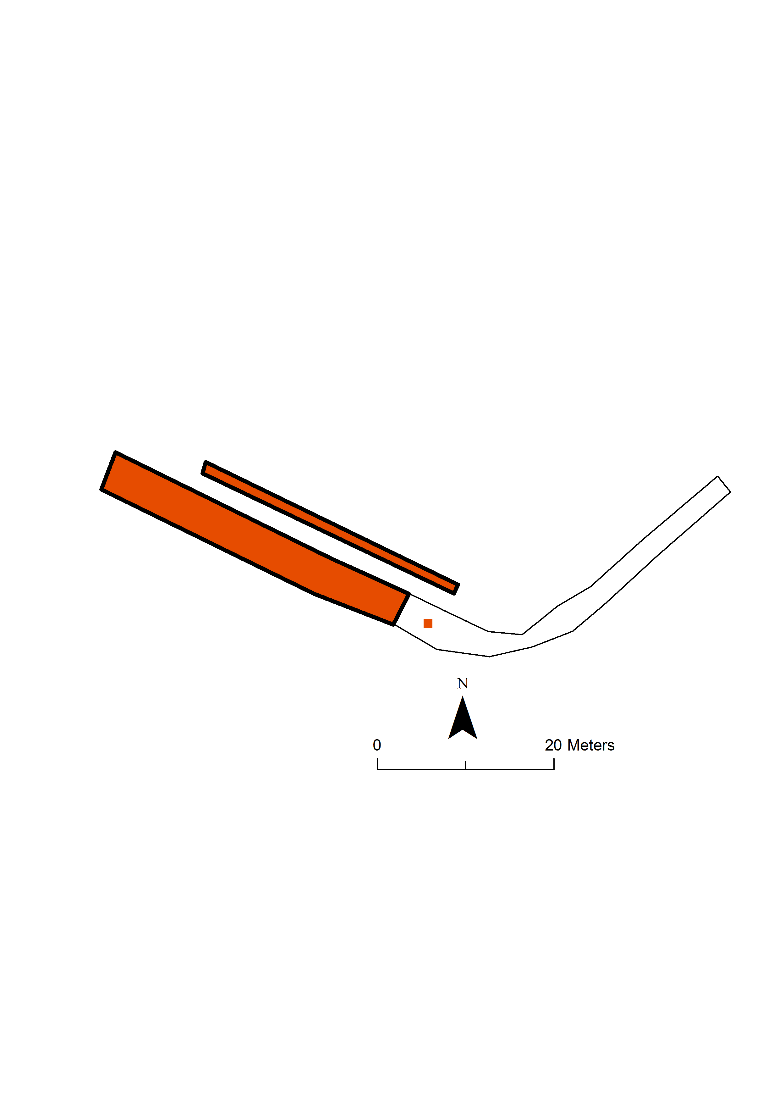

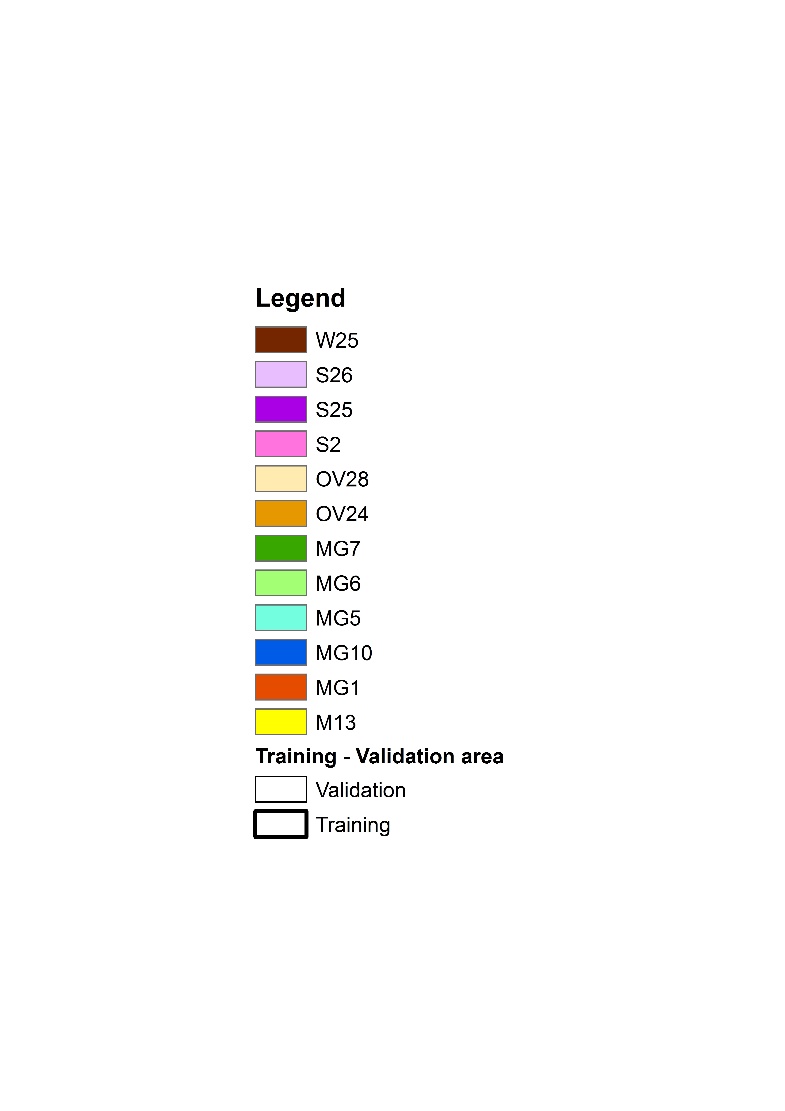


Hyperspectral Simulated 13-band Simulated 8-band


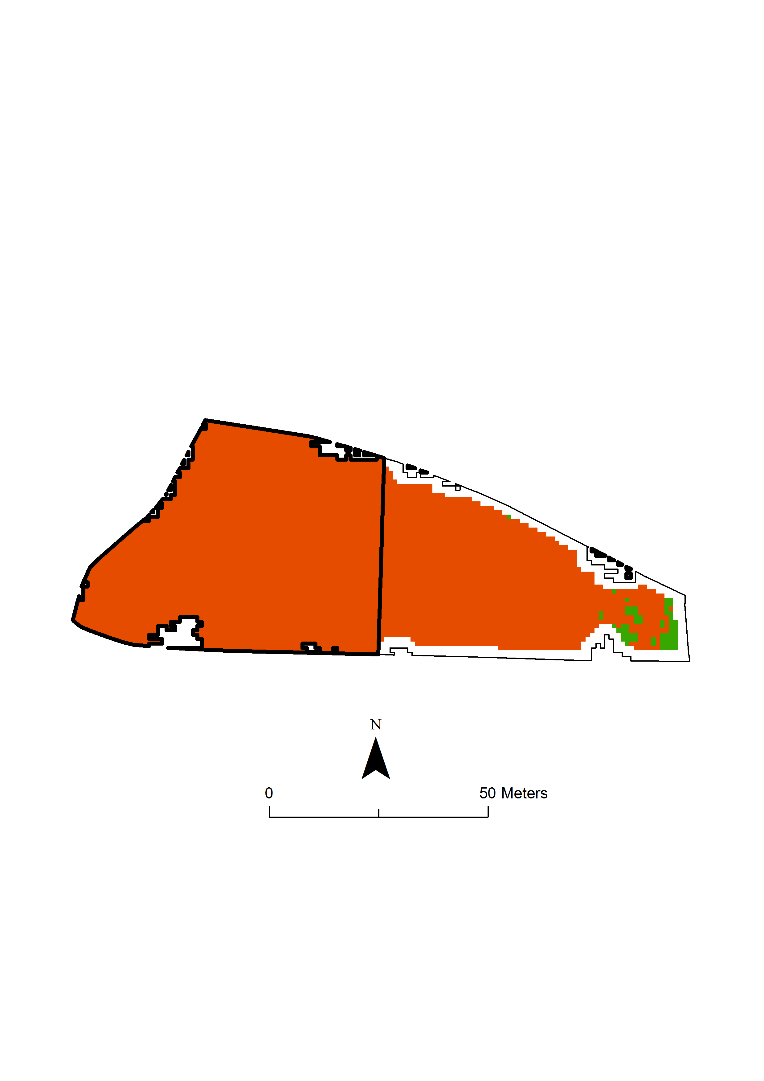

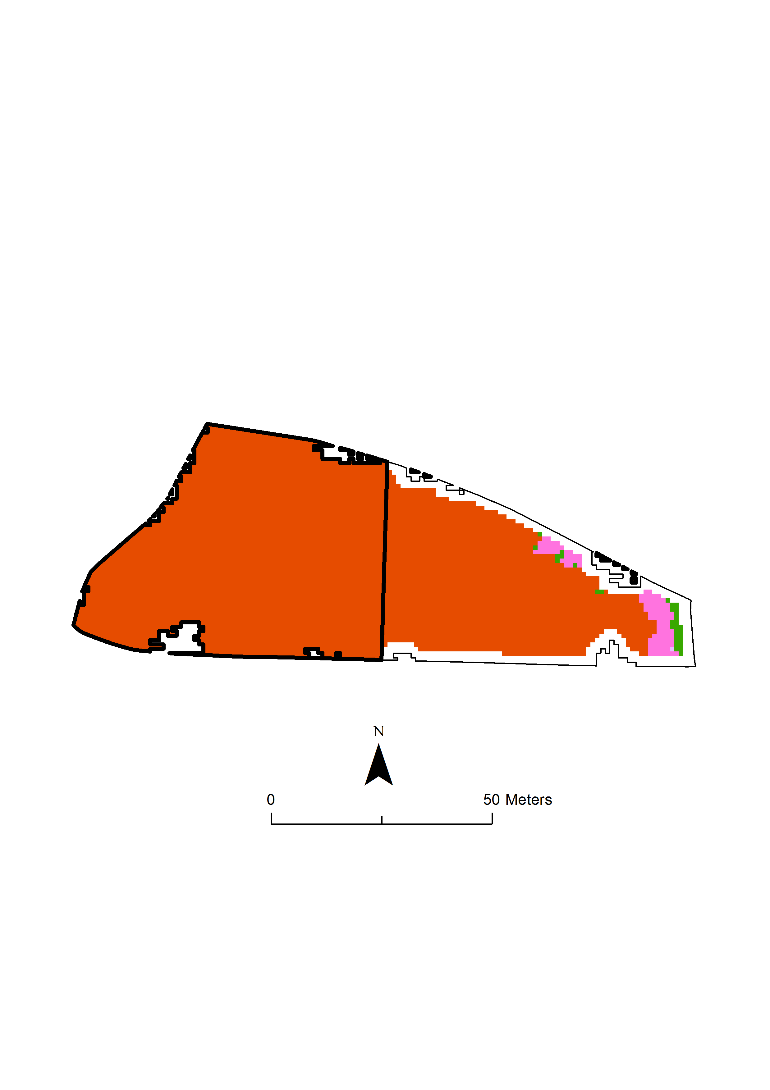

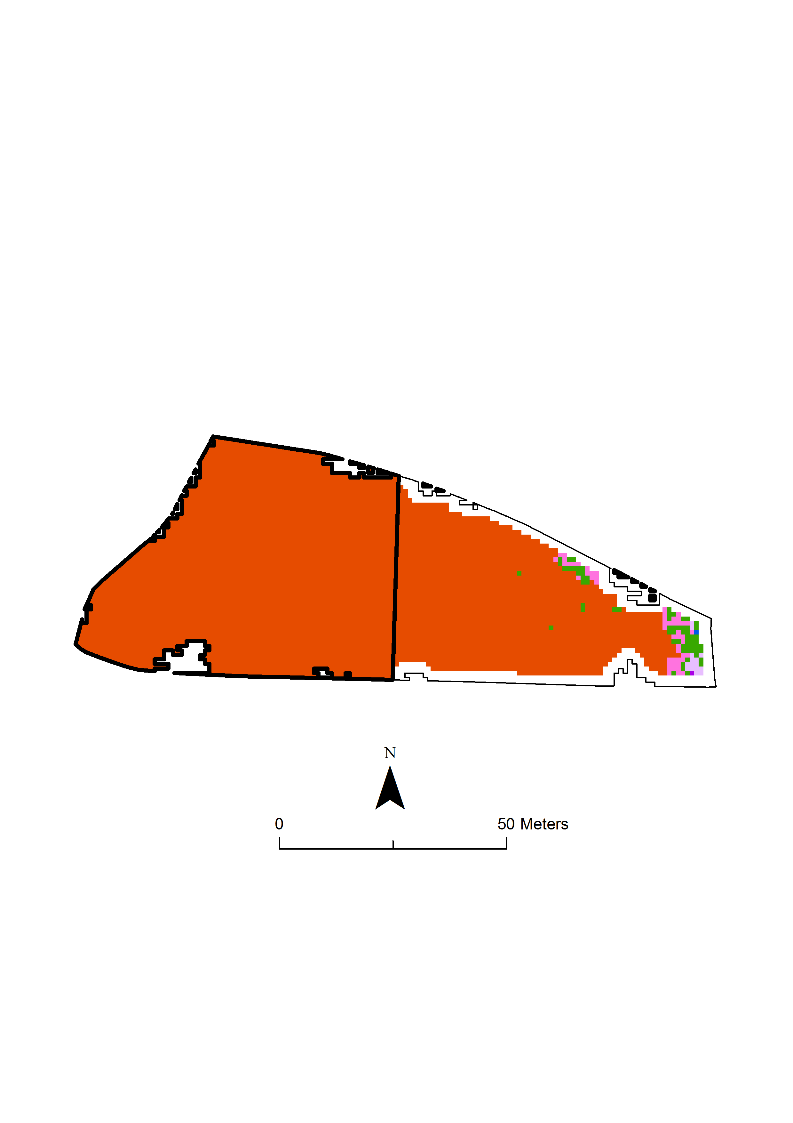

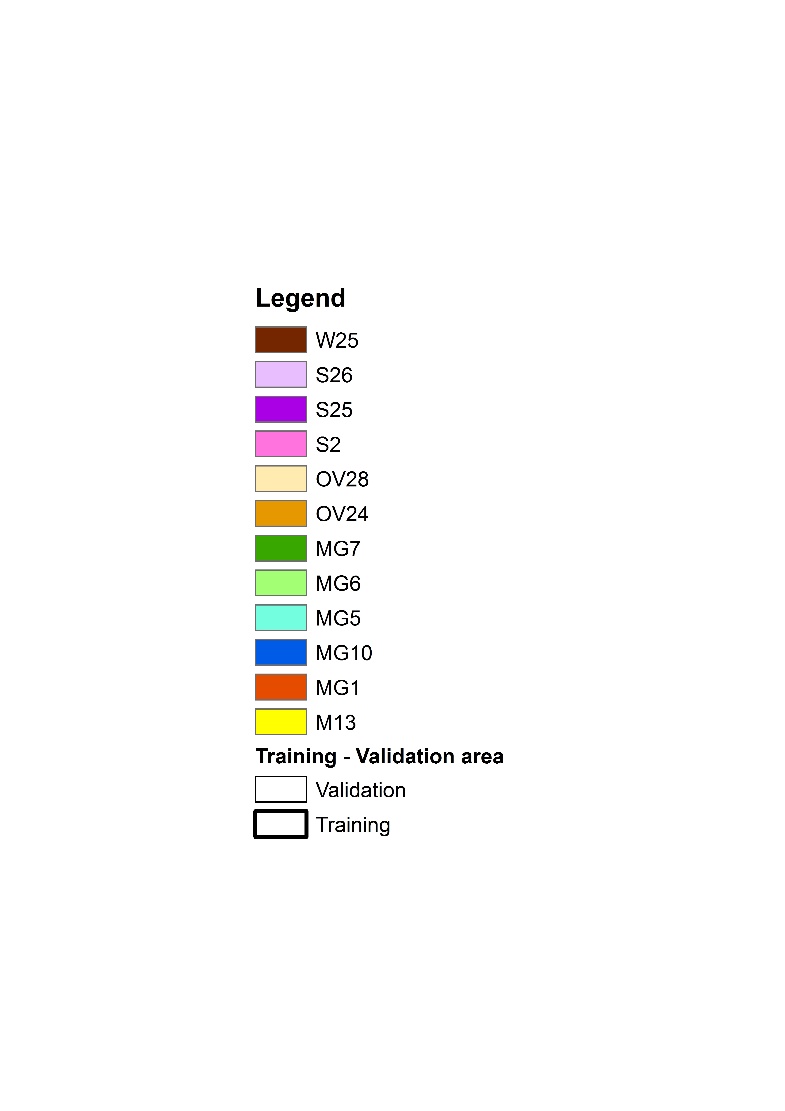


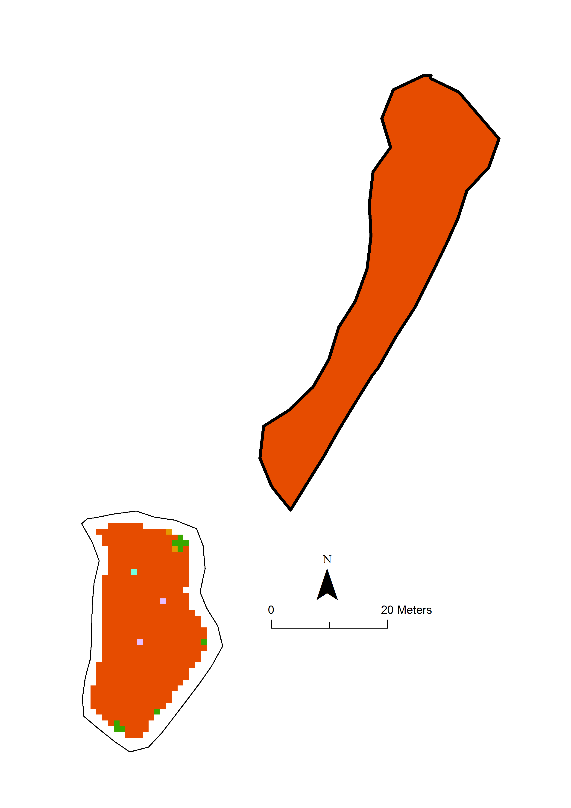

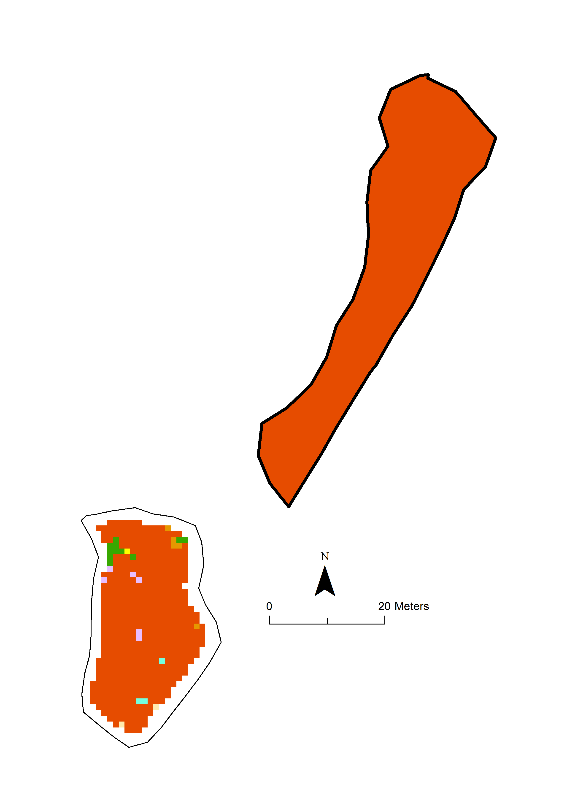

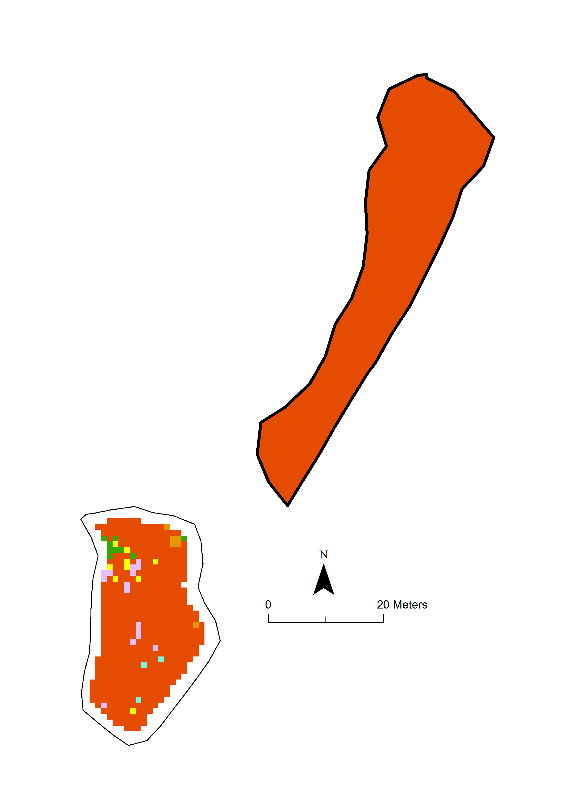


Hyperspectral Simulated 13-band Simulated 8-band


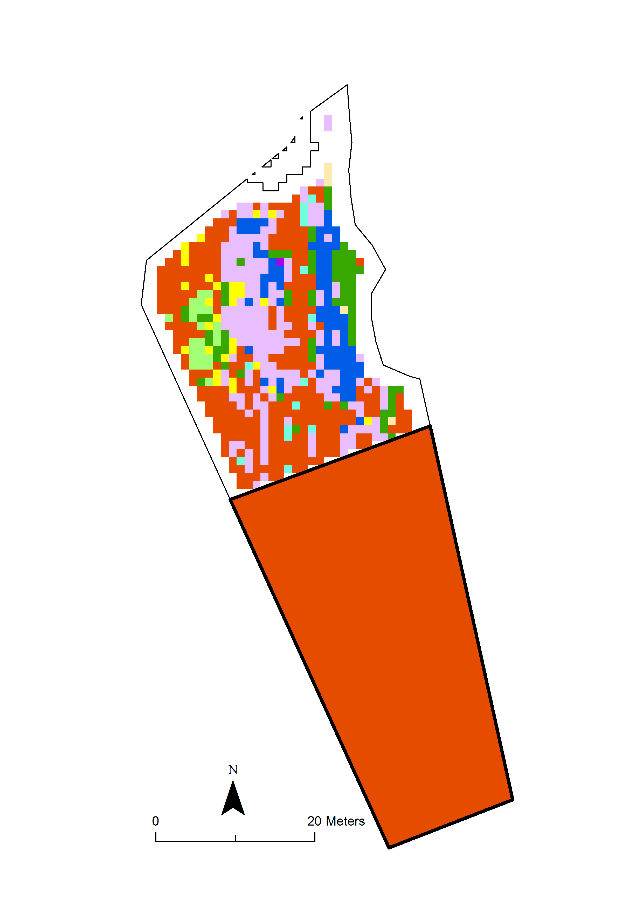

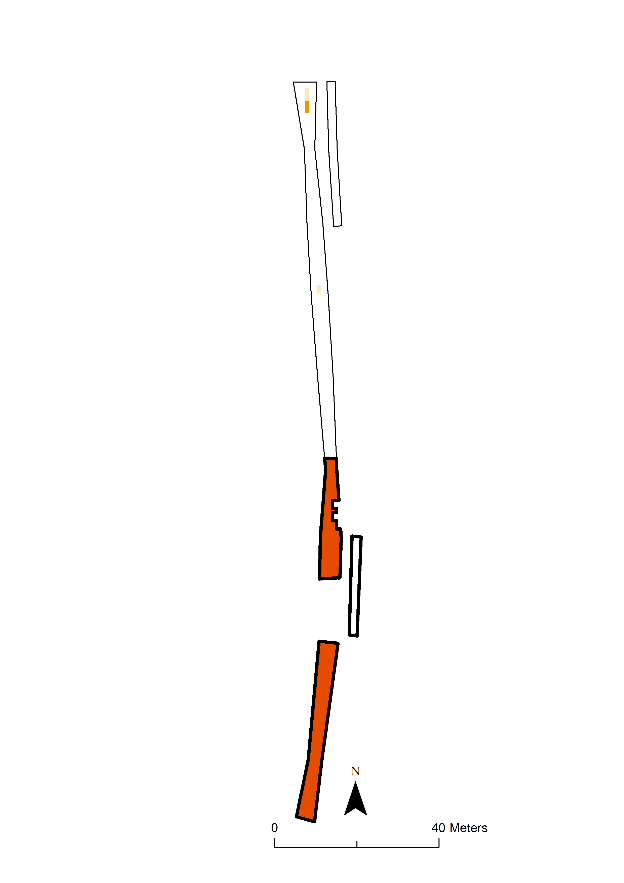

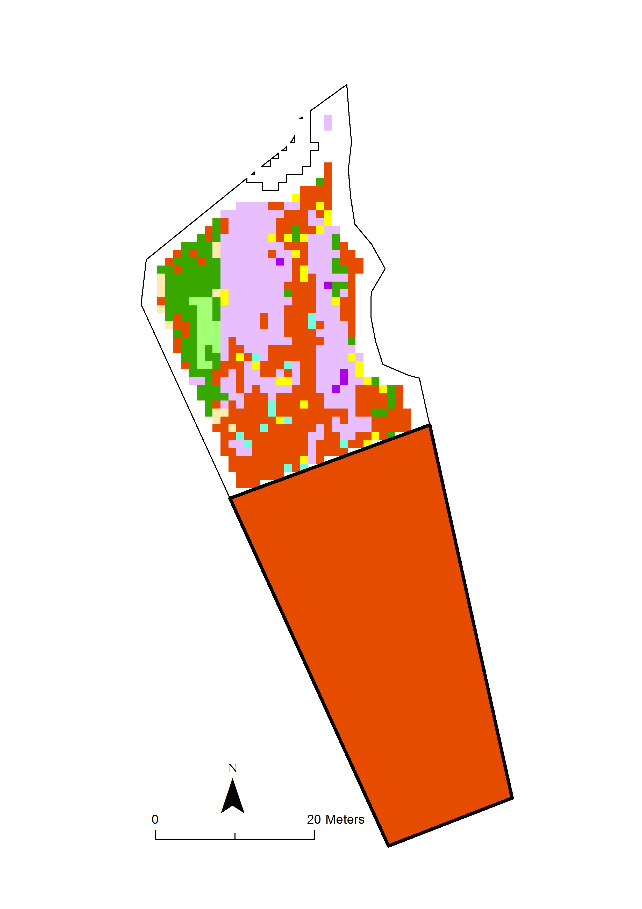

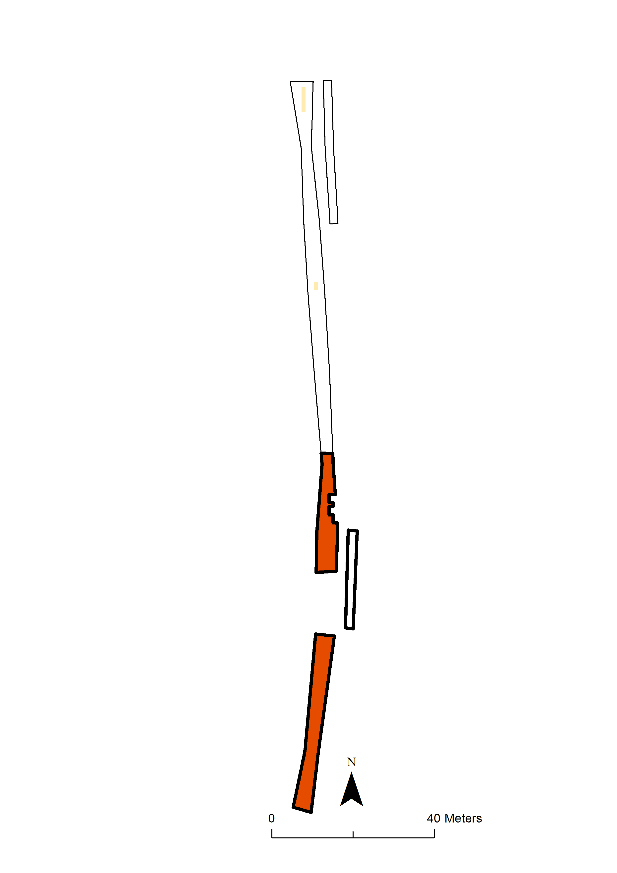

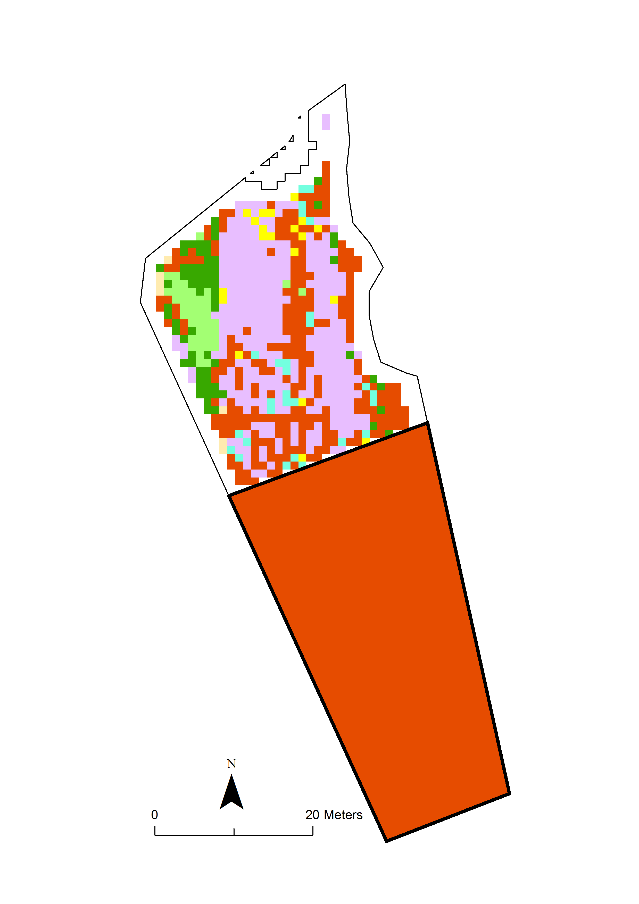

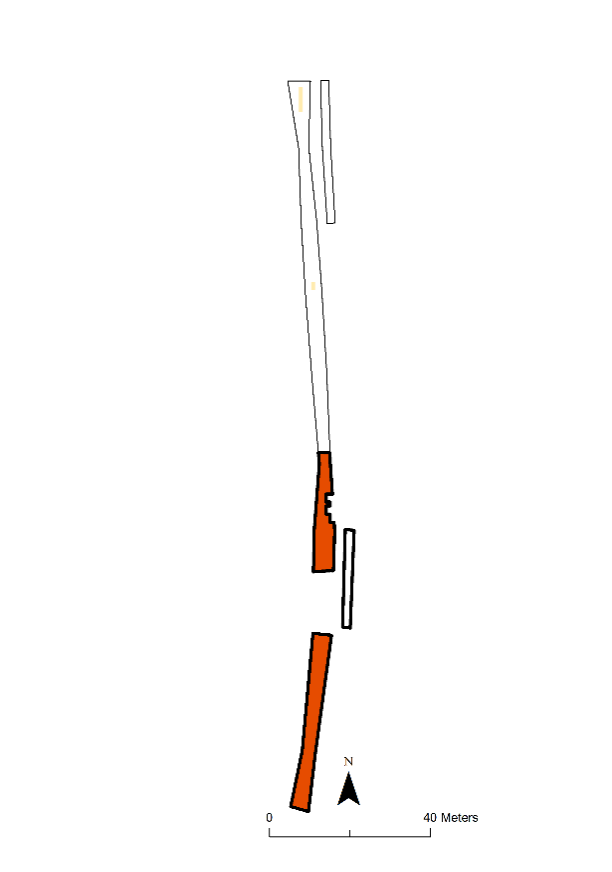

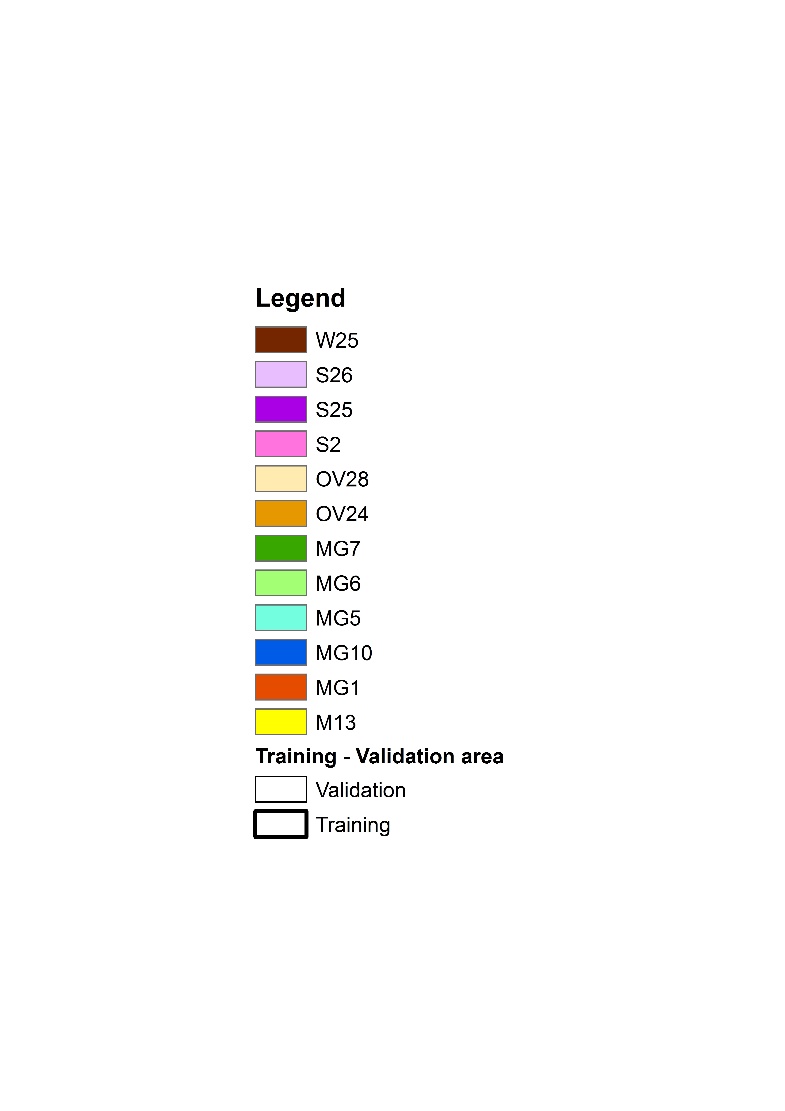


Hyperspectral Simulated 13-band Simulated 8-band


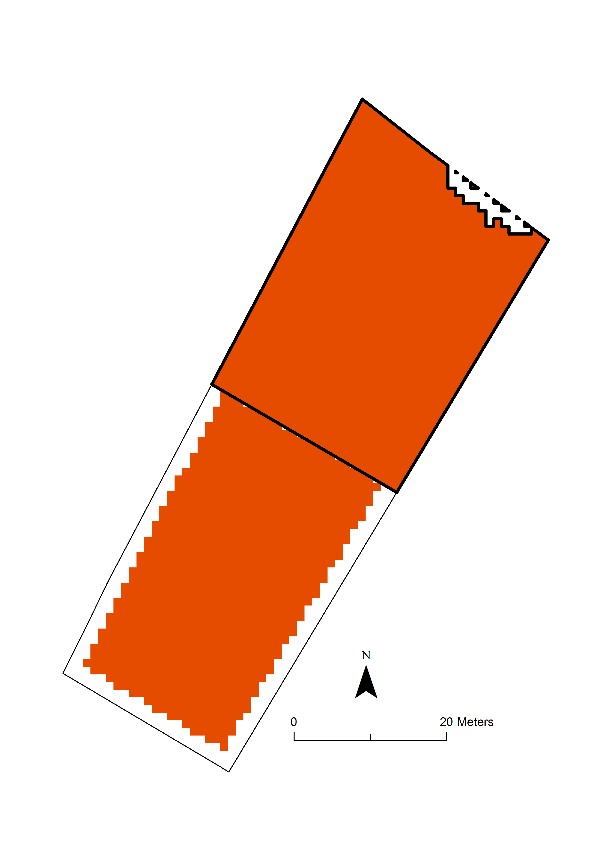

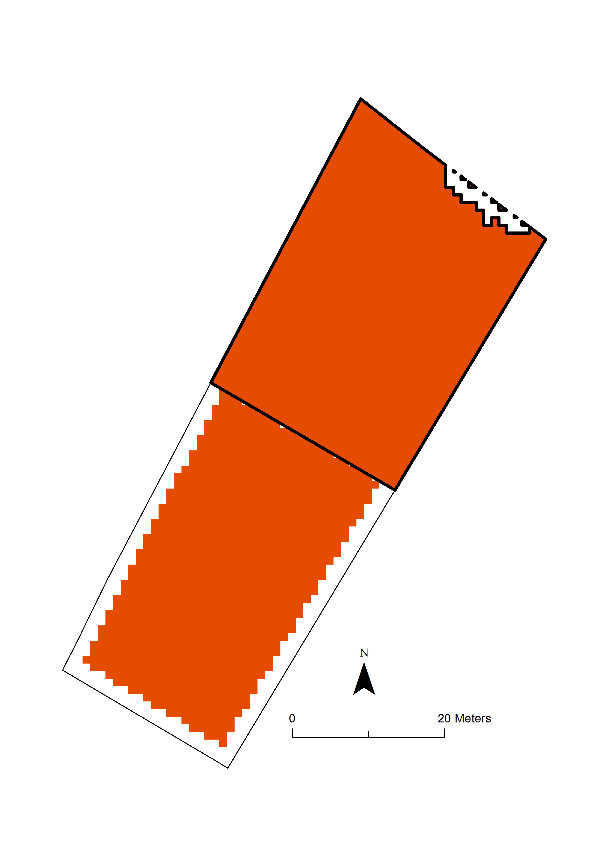

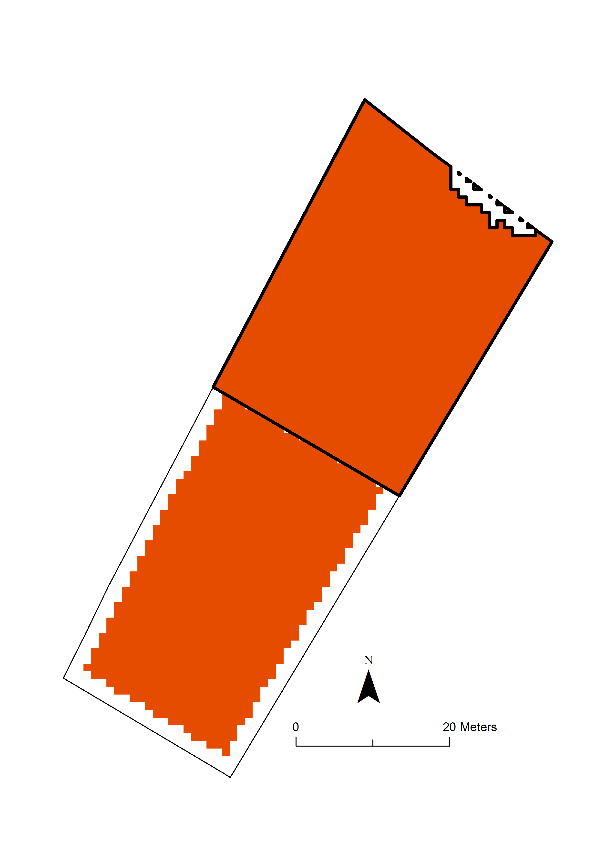

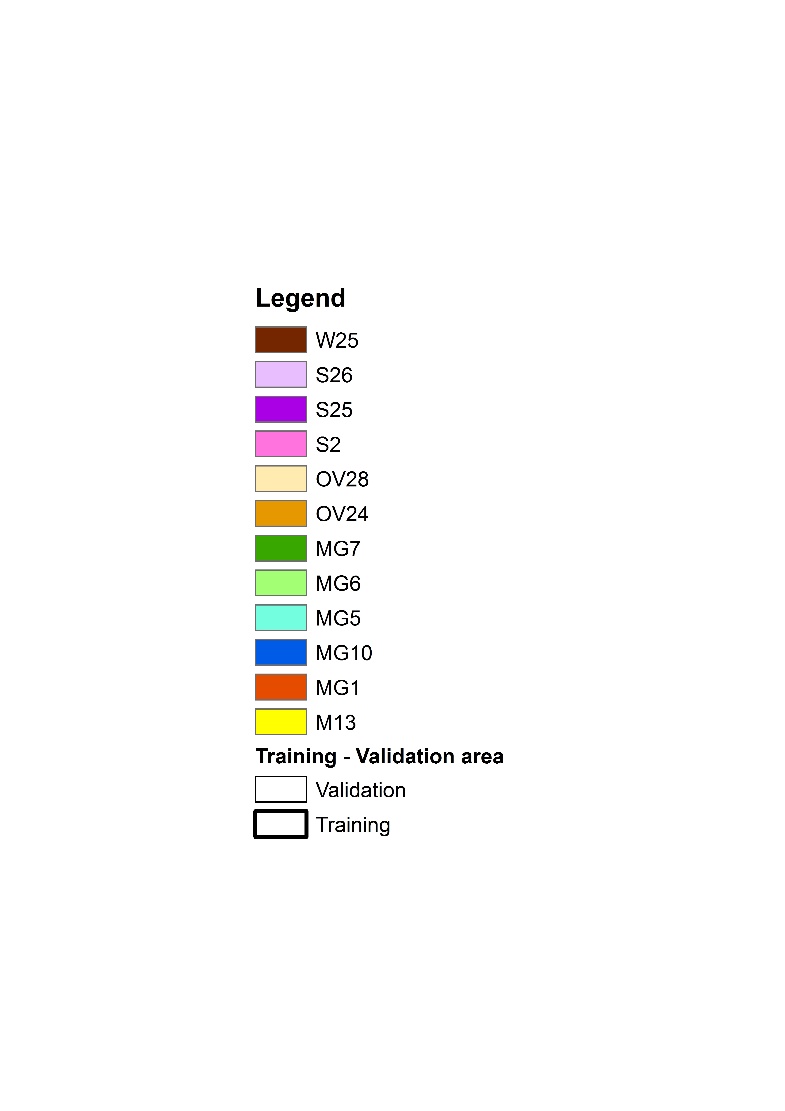


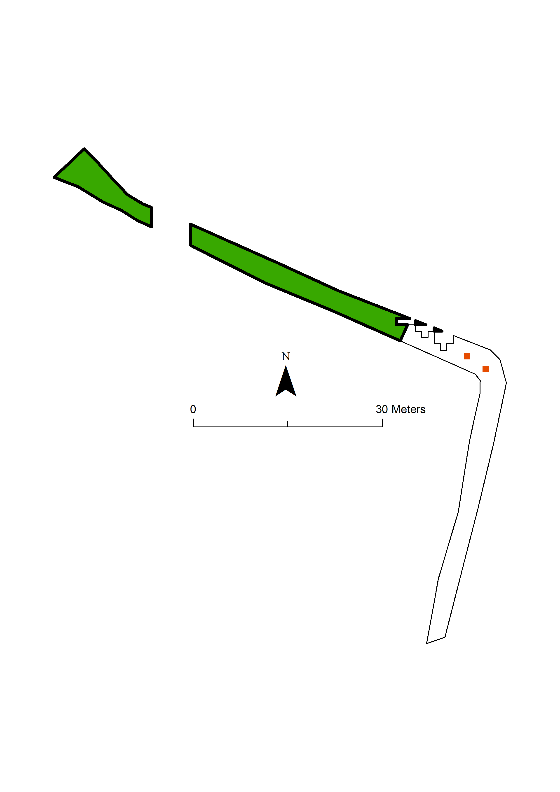

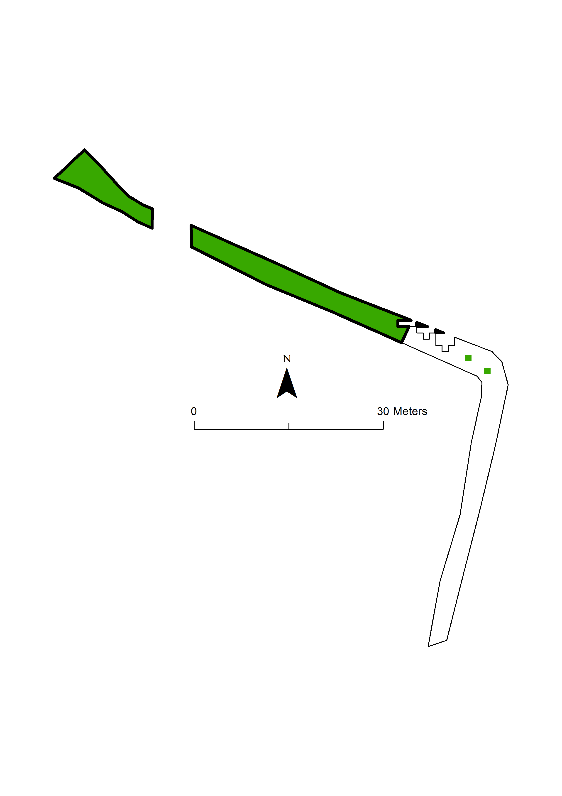

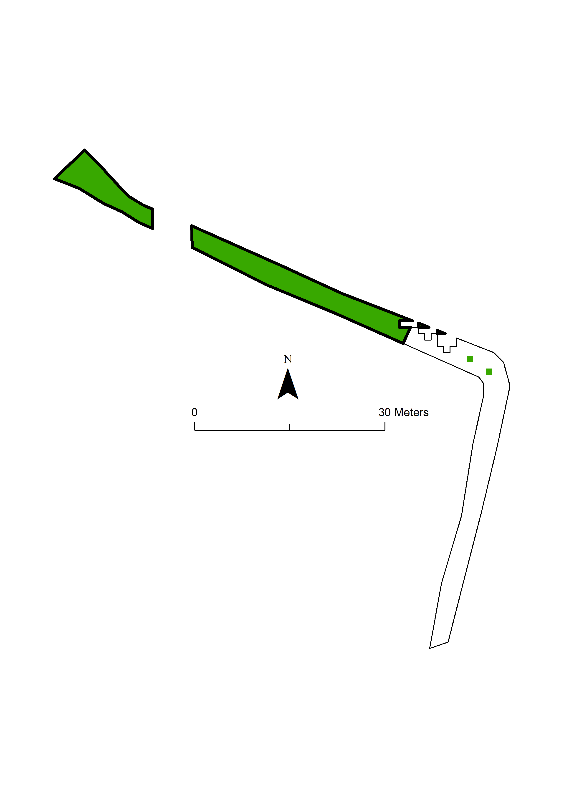


Hyperspectral Simulated 13-band Simulated 8-band


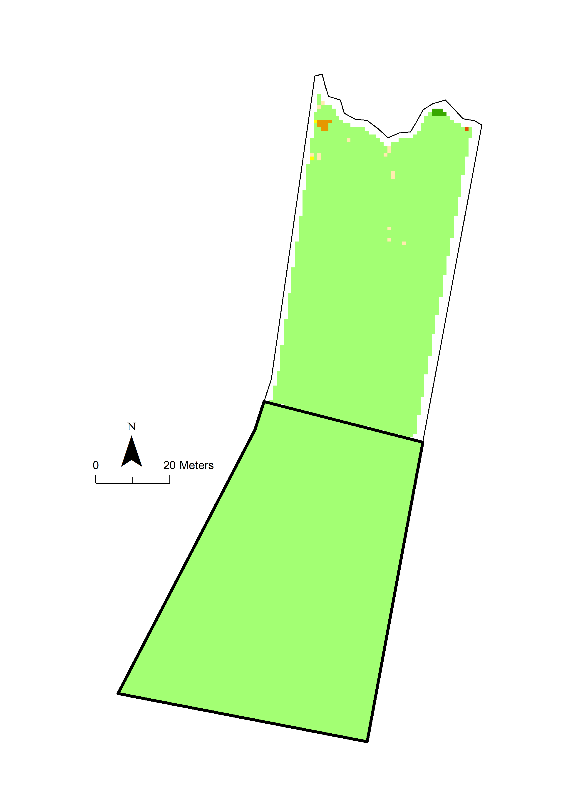

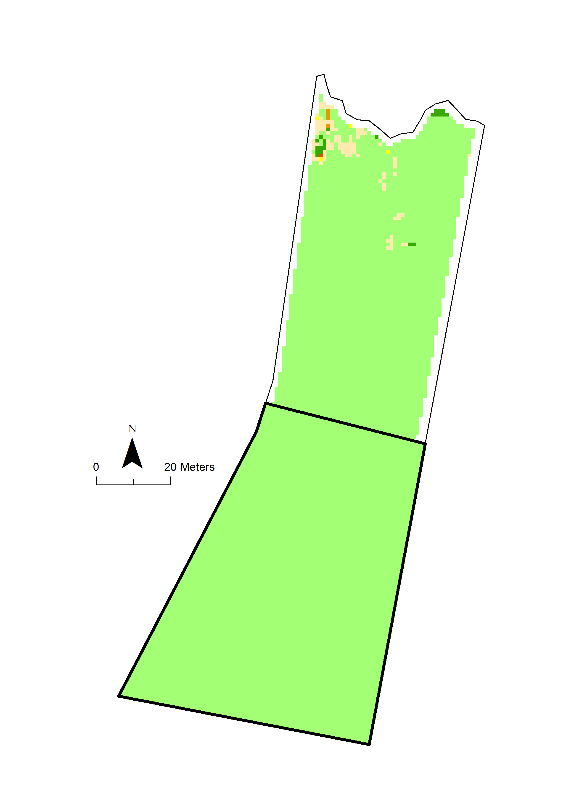

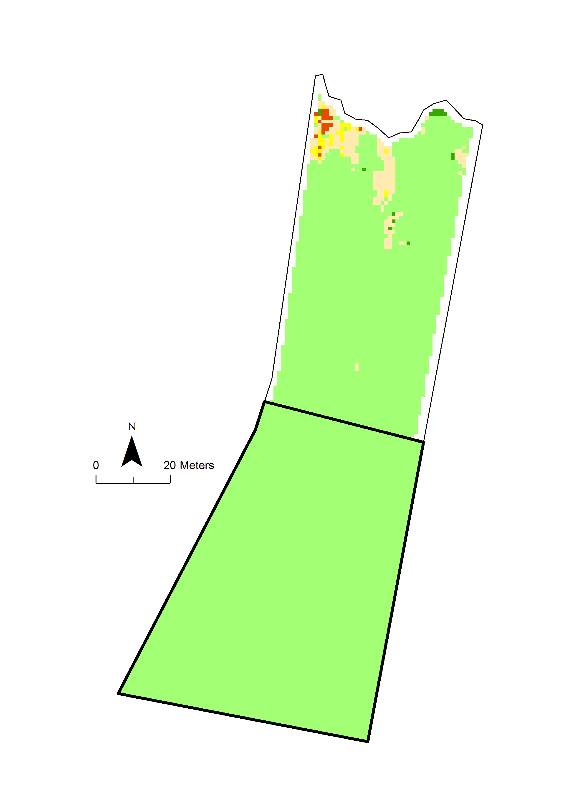

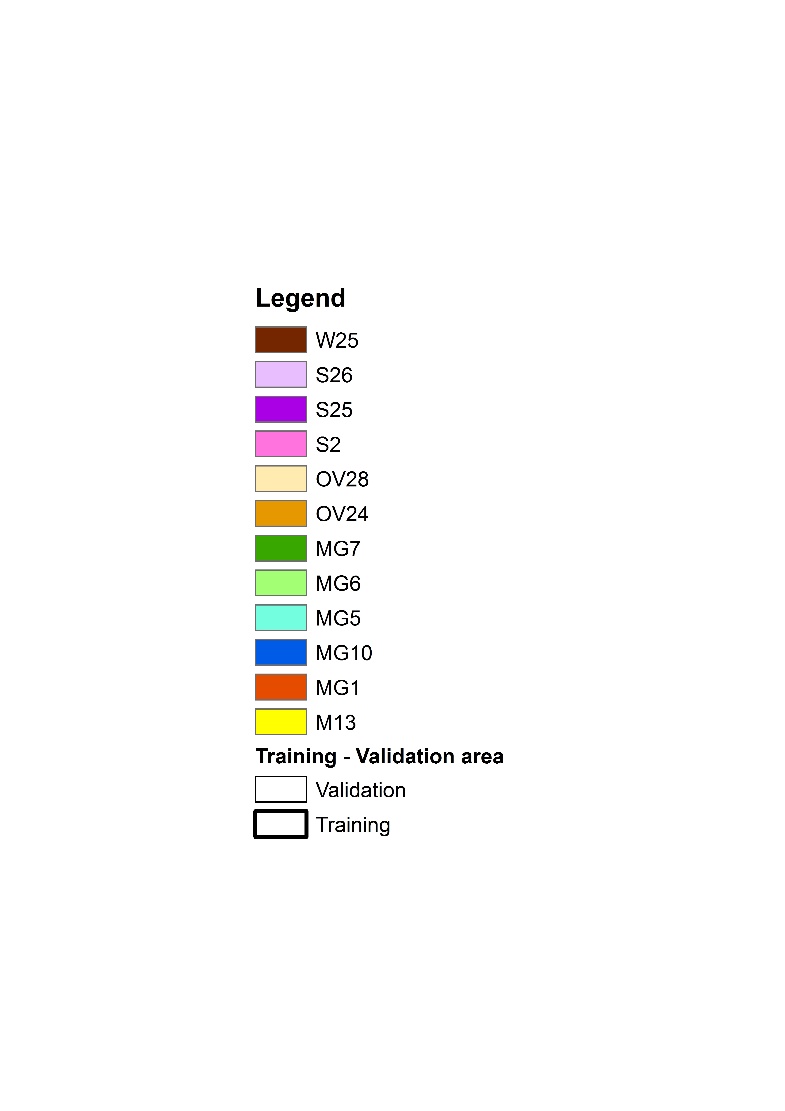


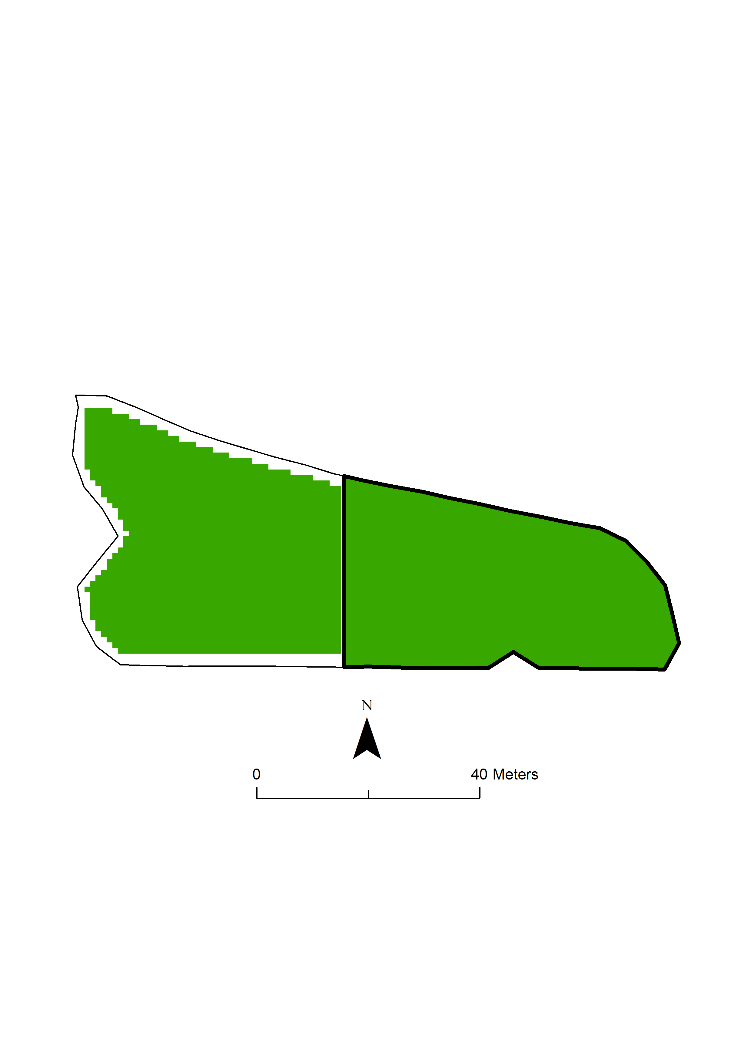

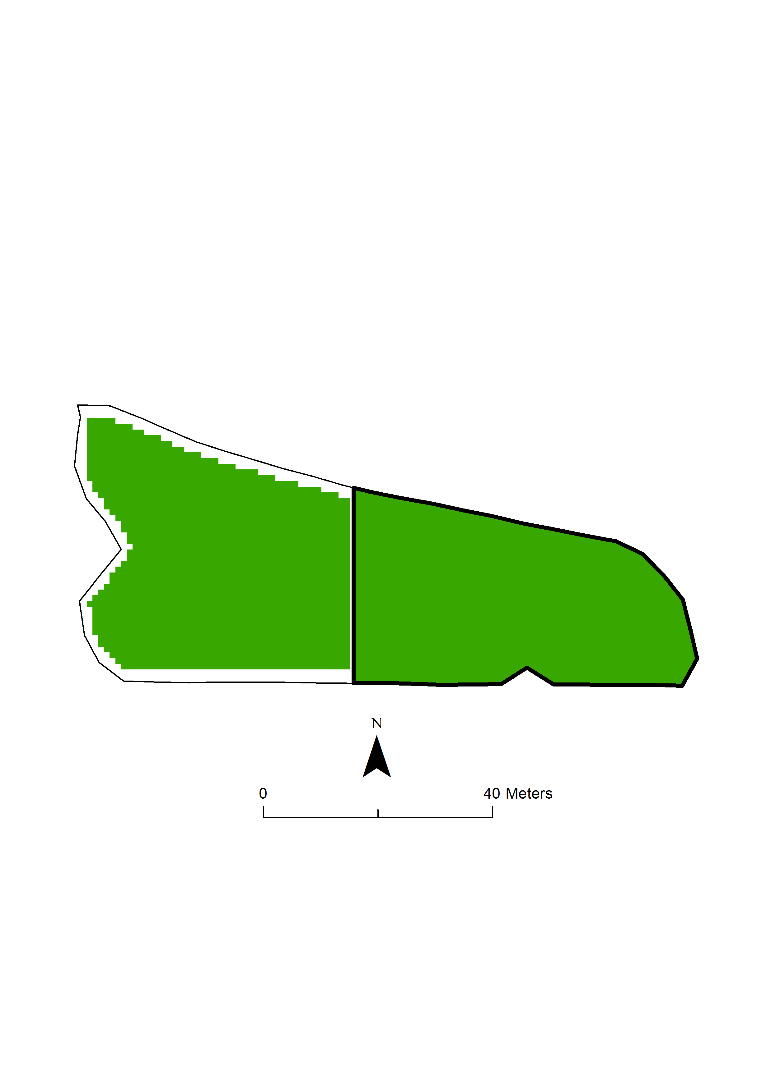

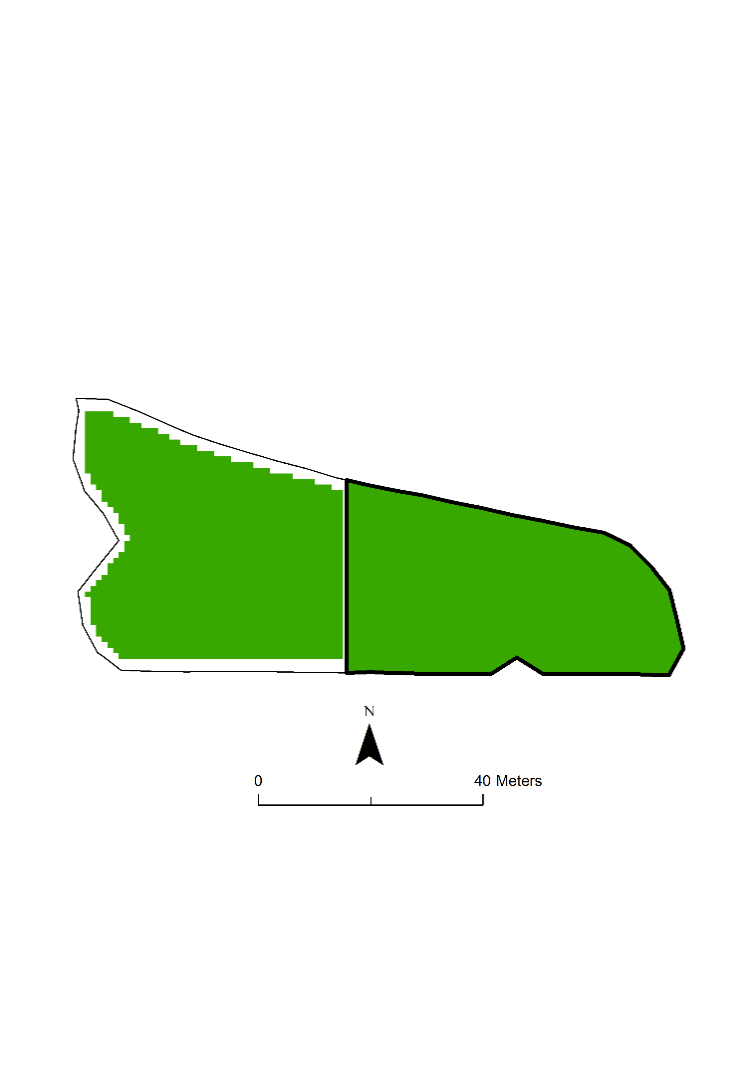


Hyperspectral Simulated 13-band Simulated 8-band


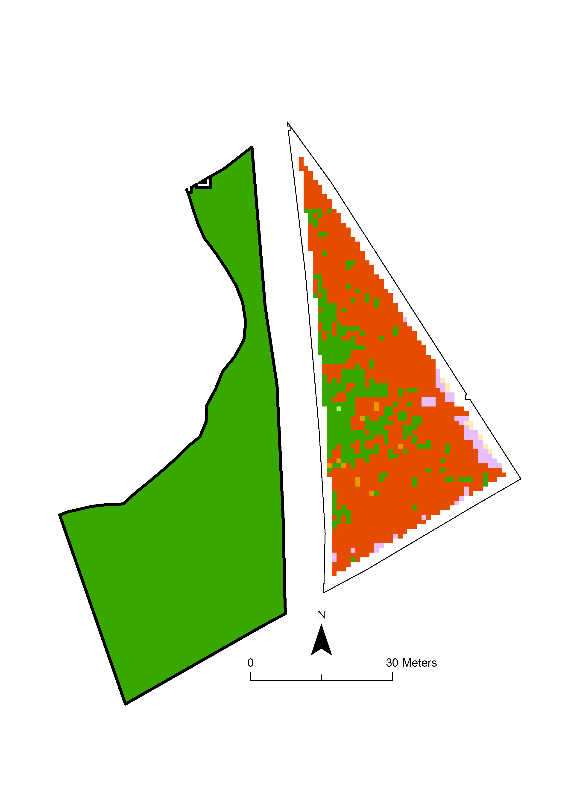

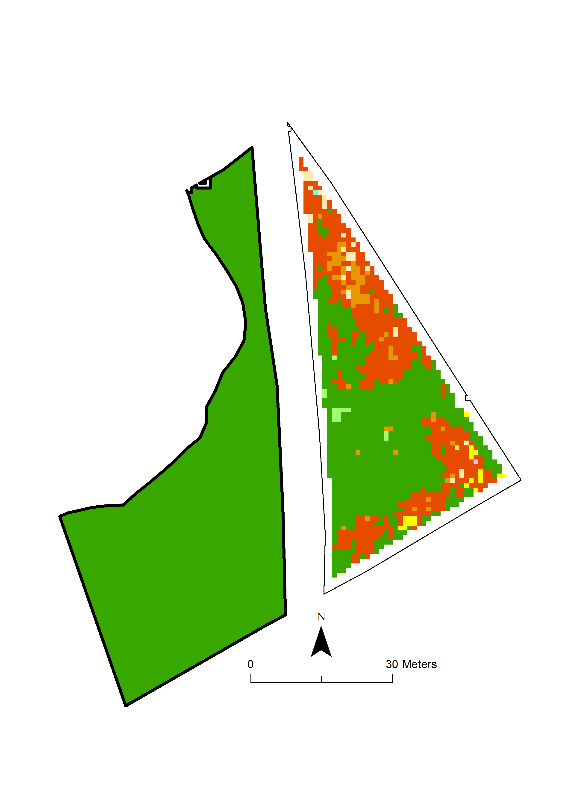

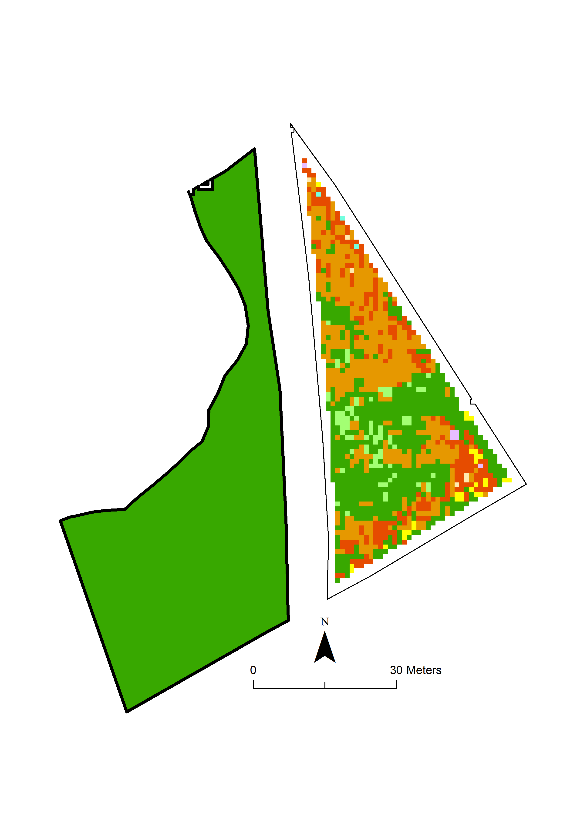

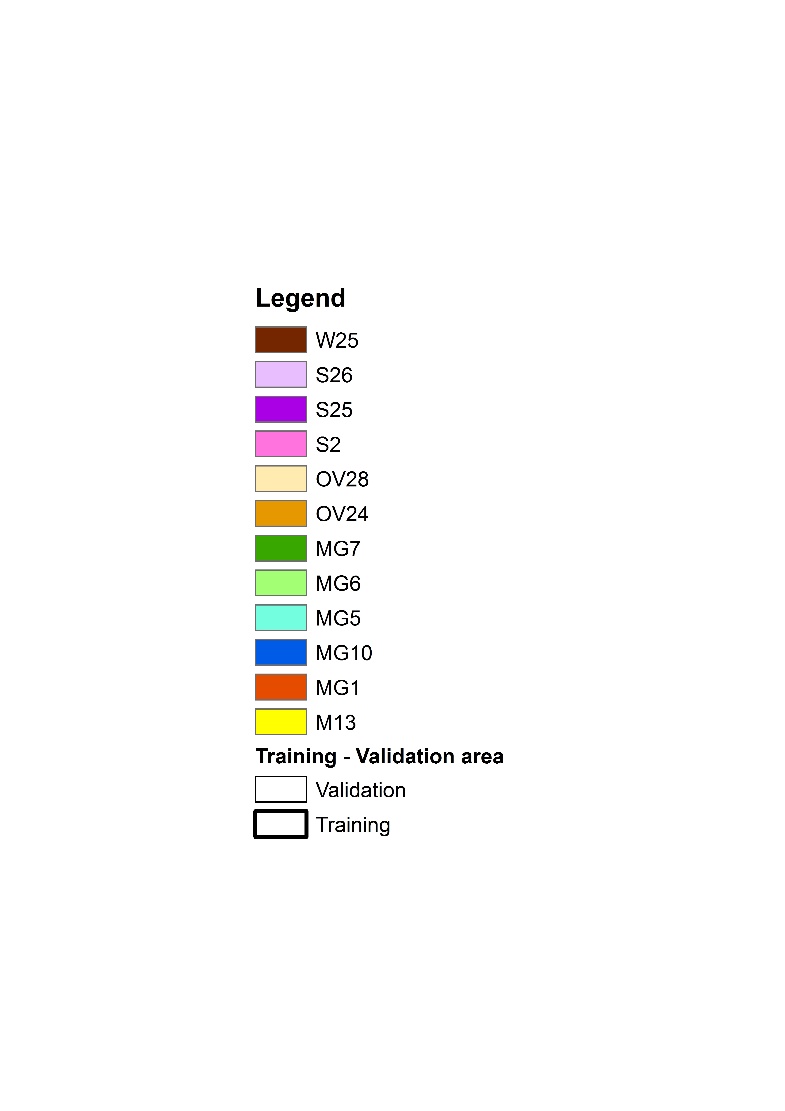


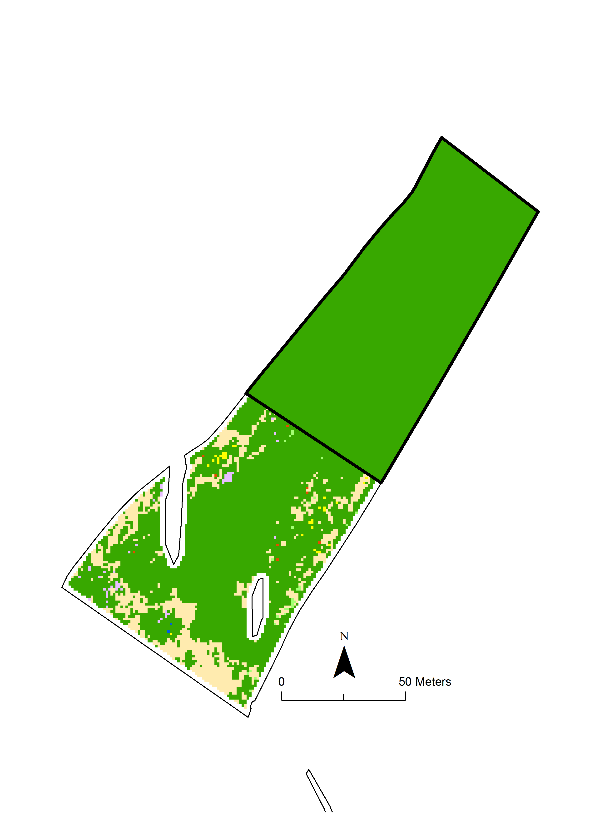

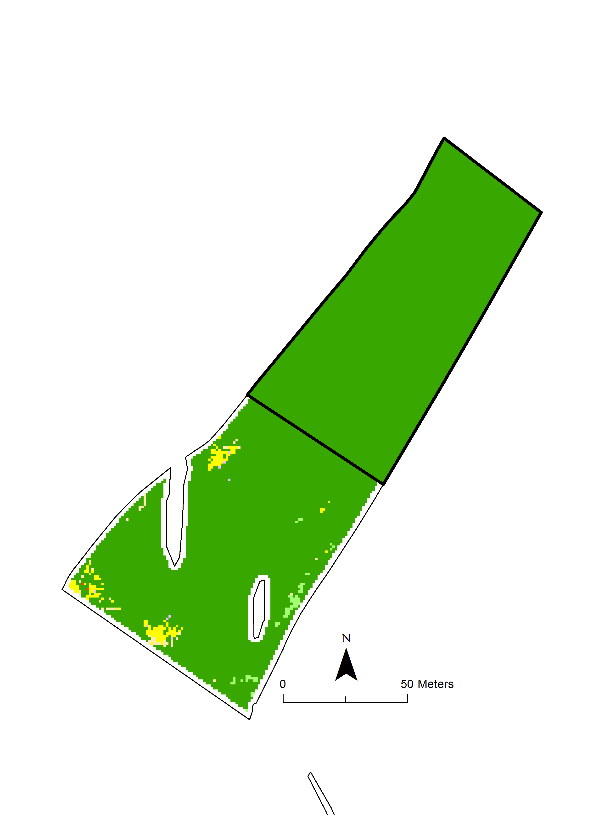

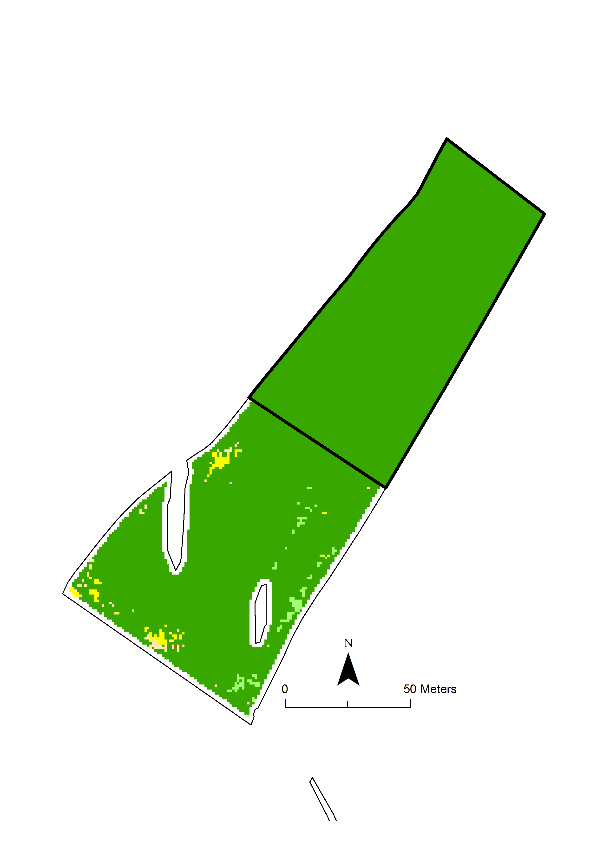


Hyperspectral Simulated 13-band Simulated 8-band


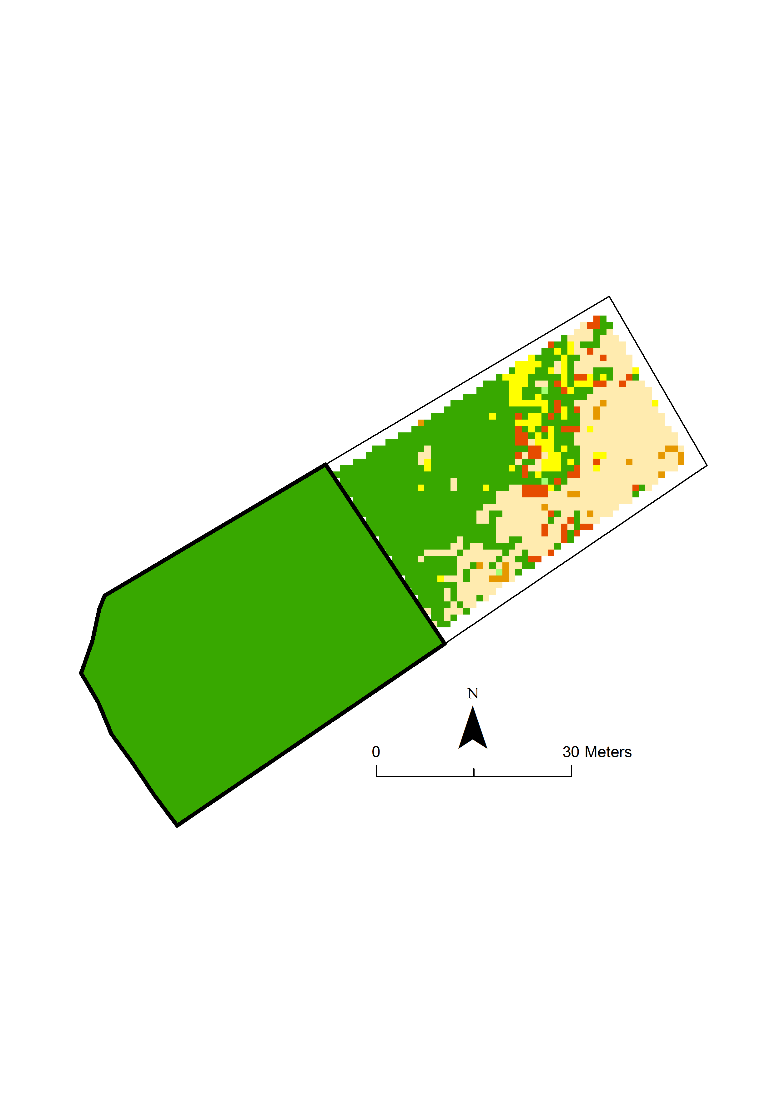

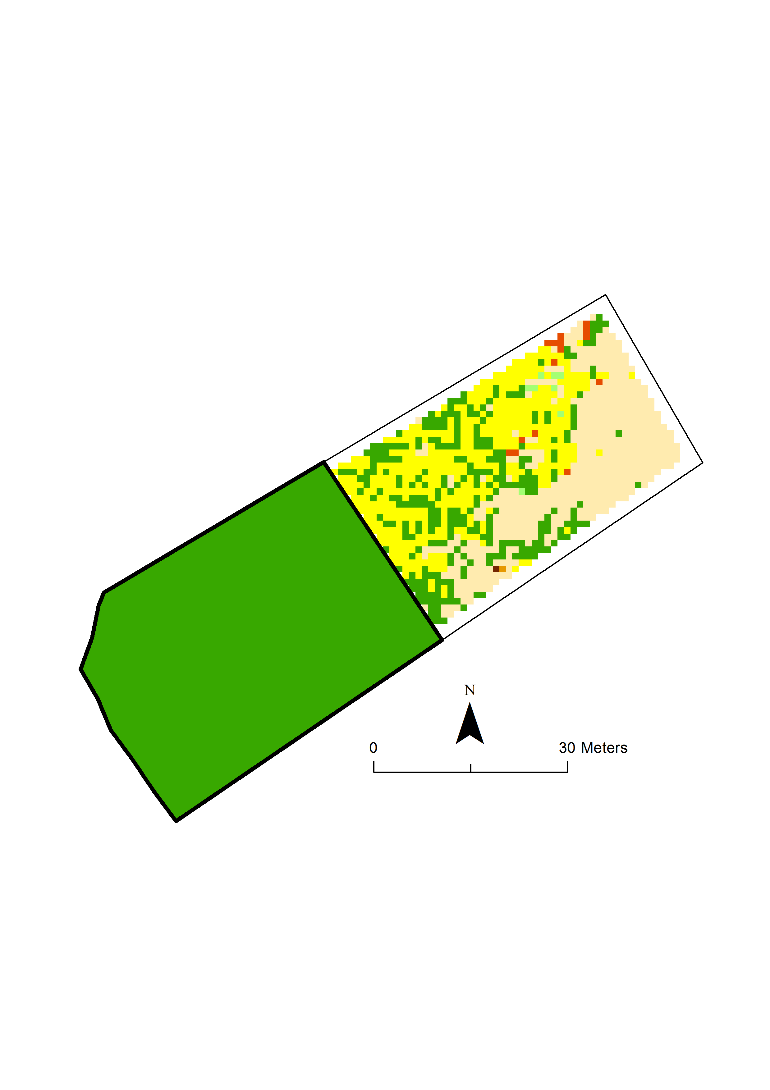

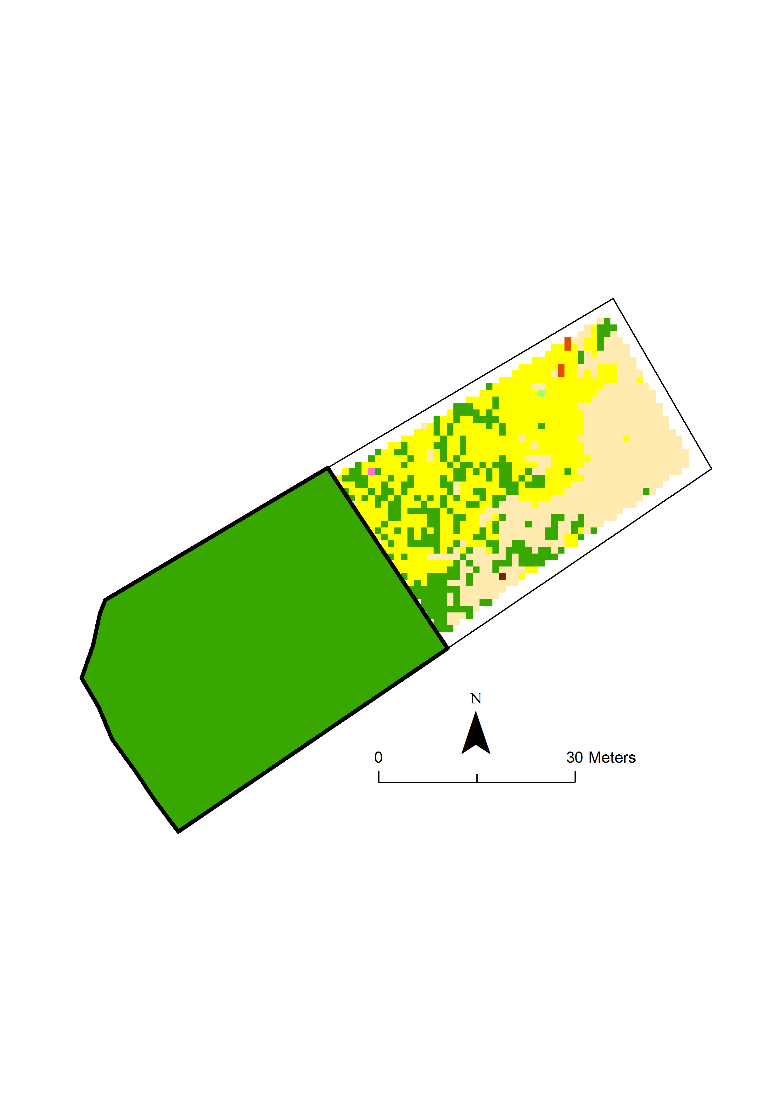

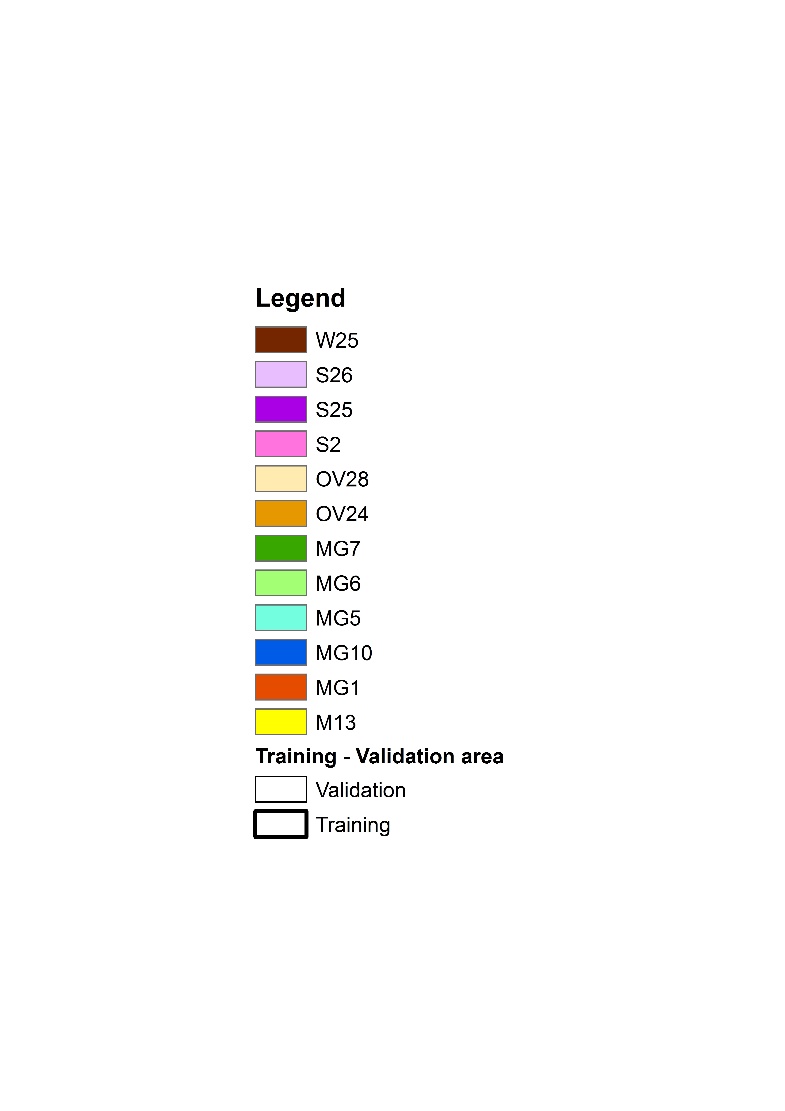


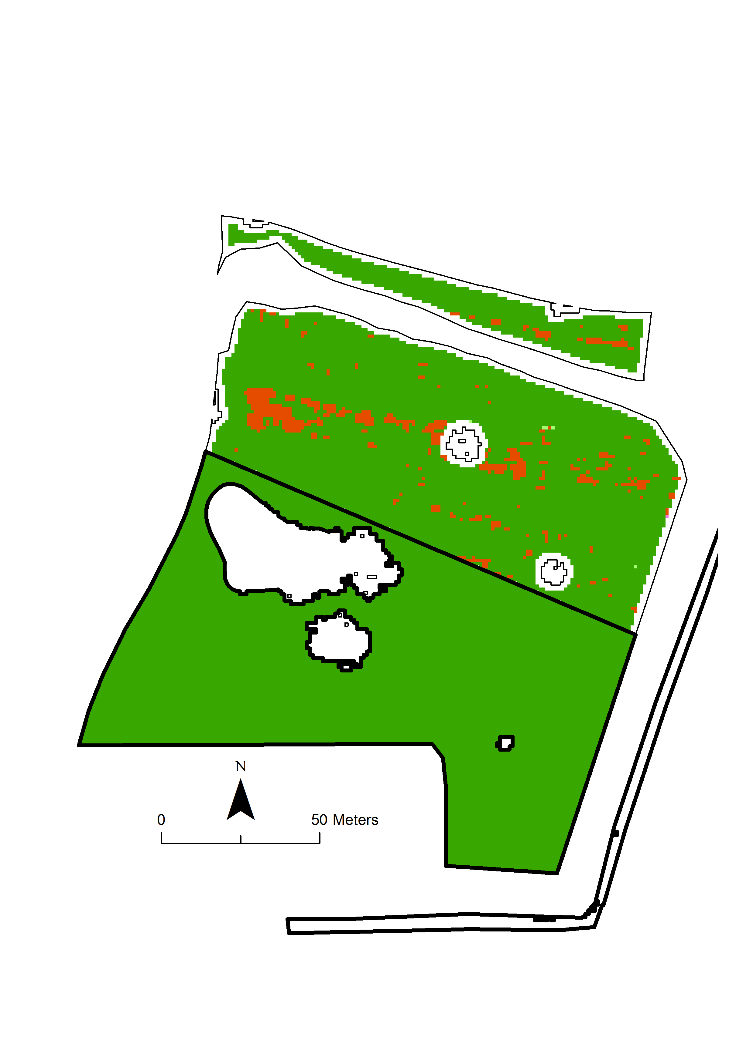

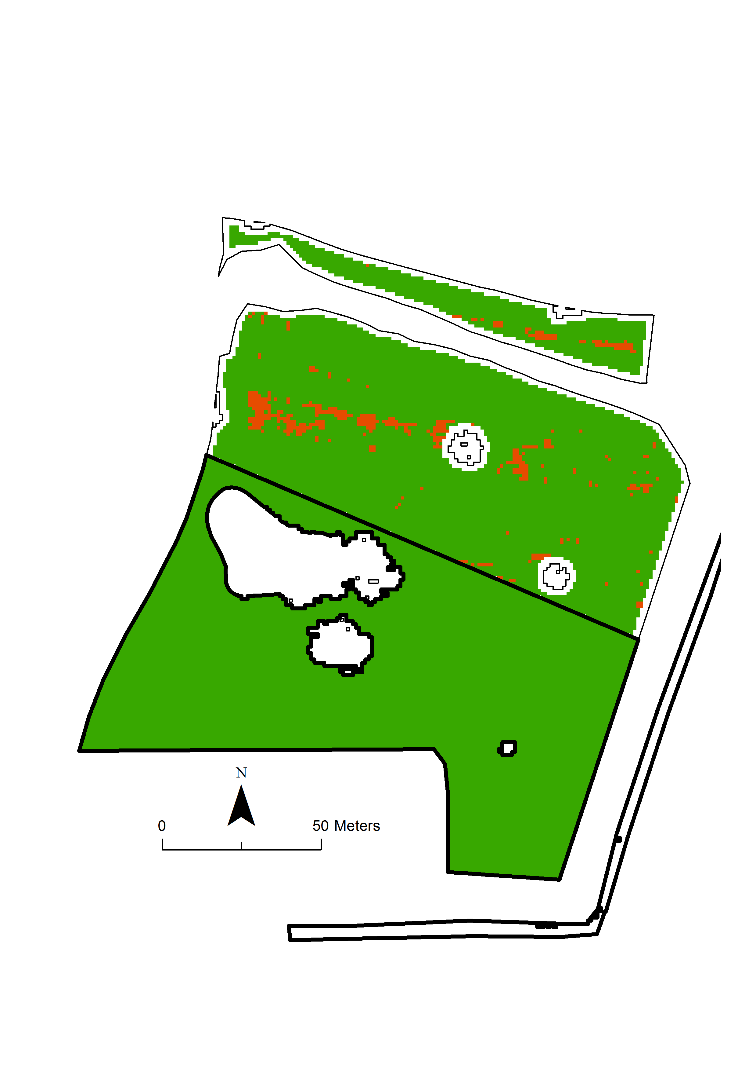

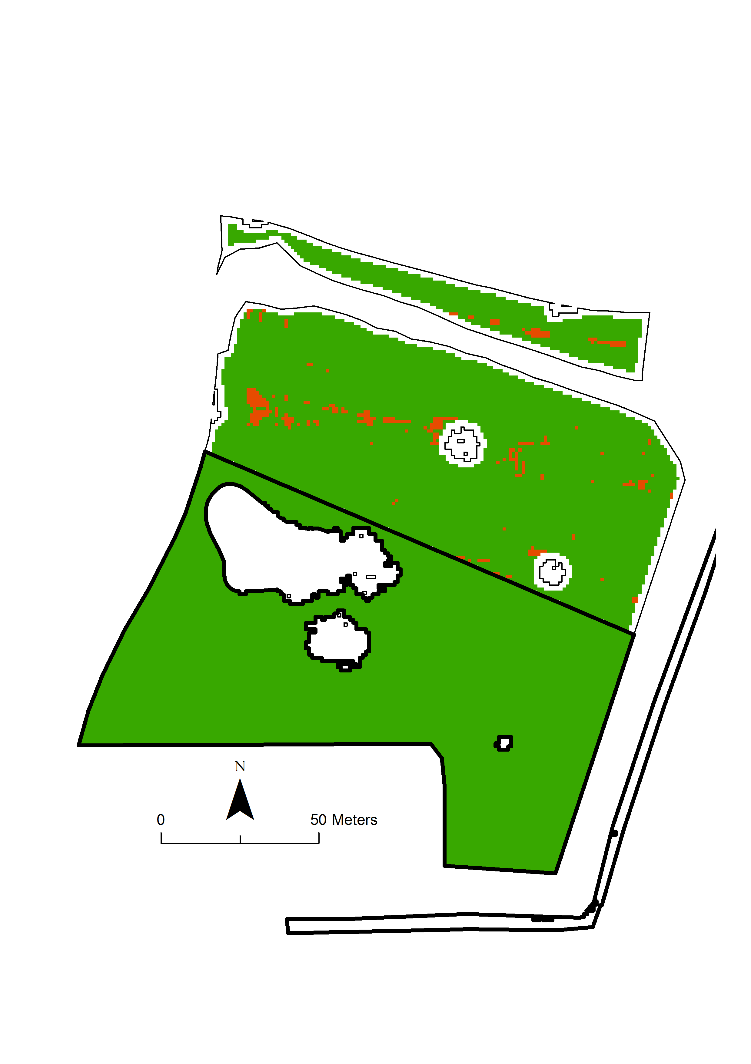


Hyperspectral Simulated 13-band Simulated 8-band


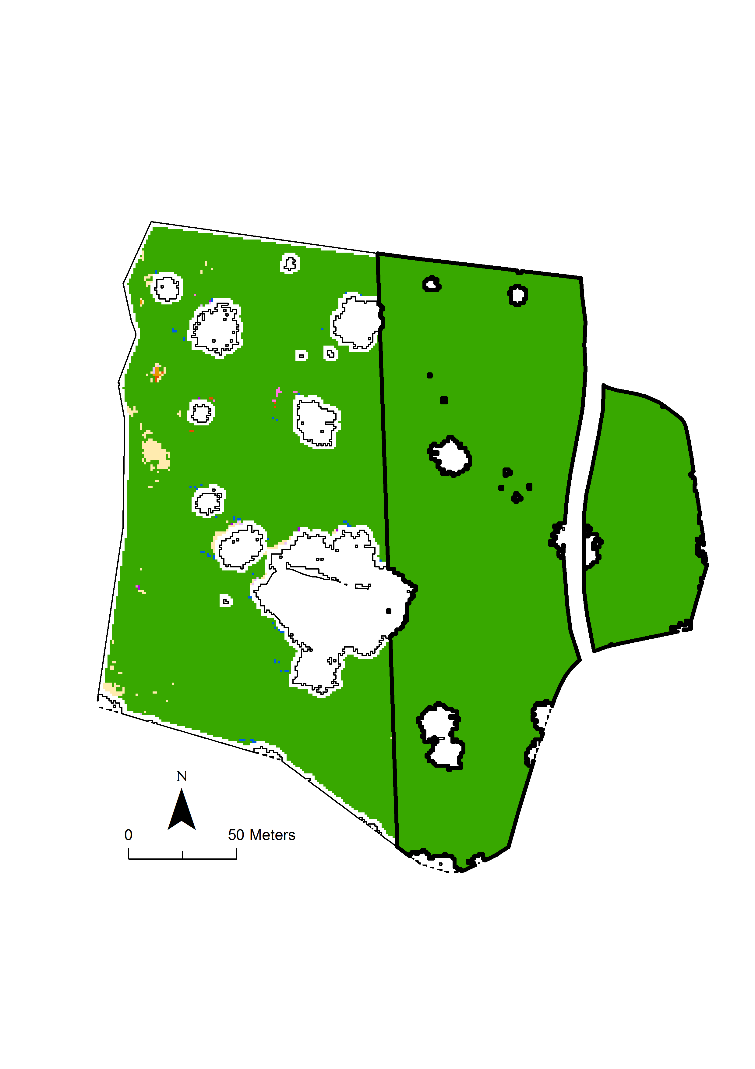

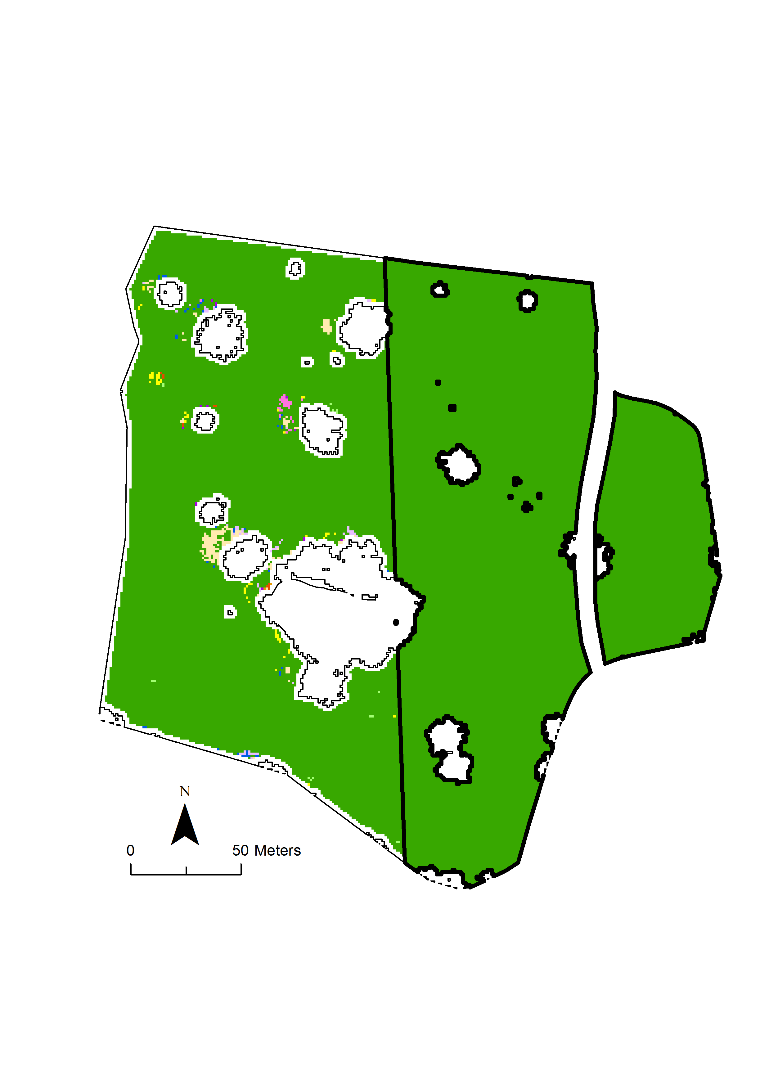

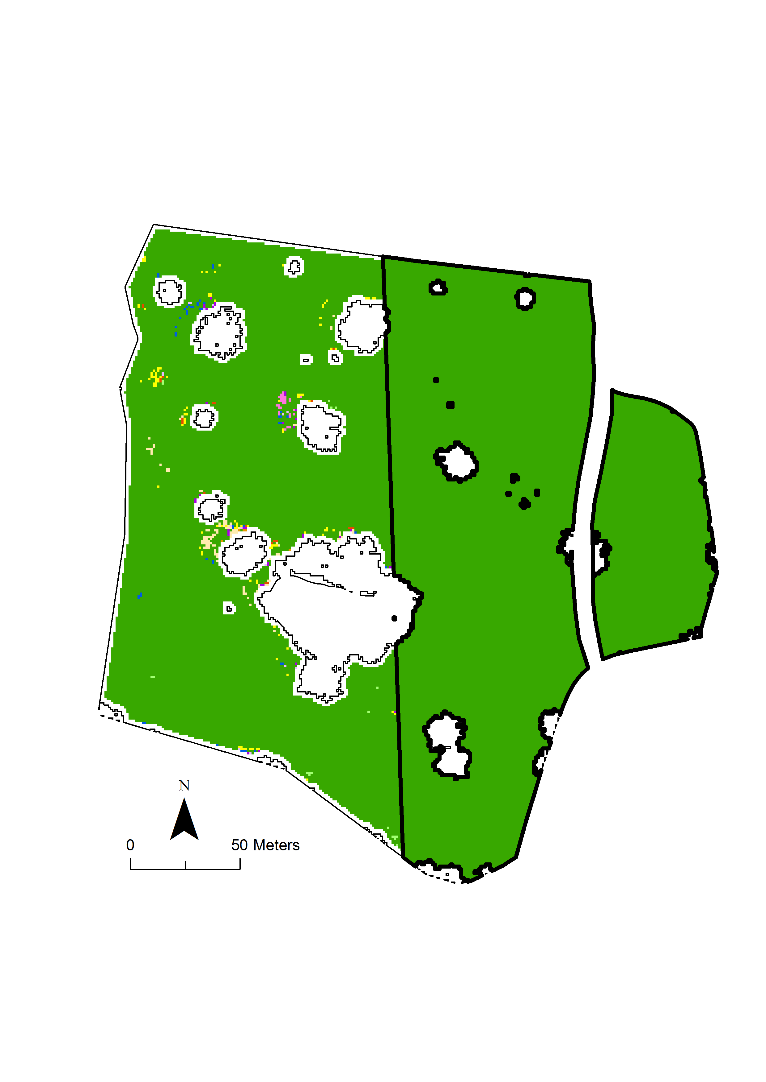

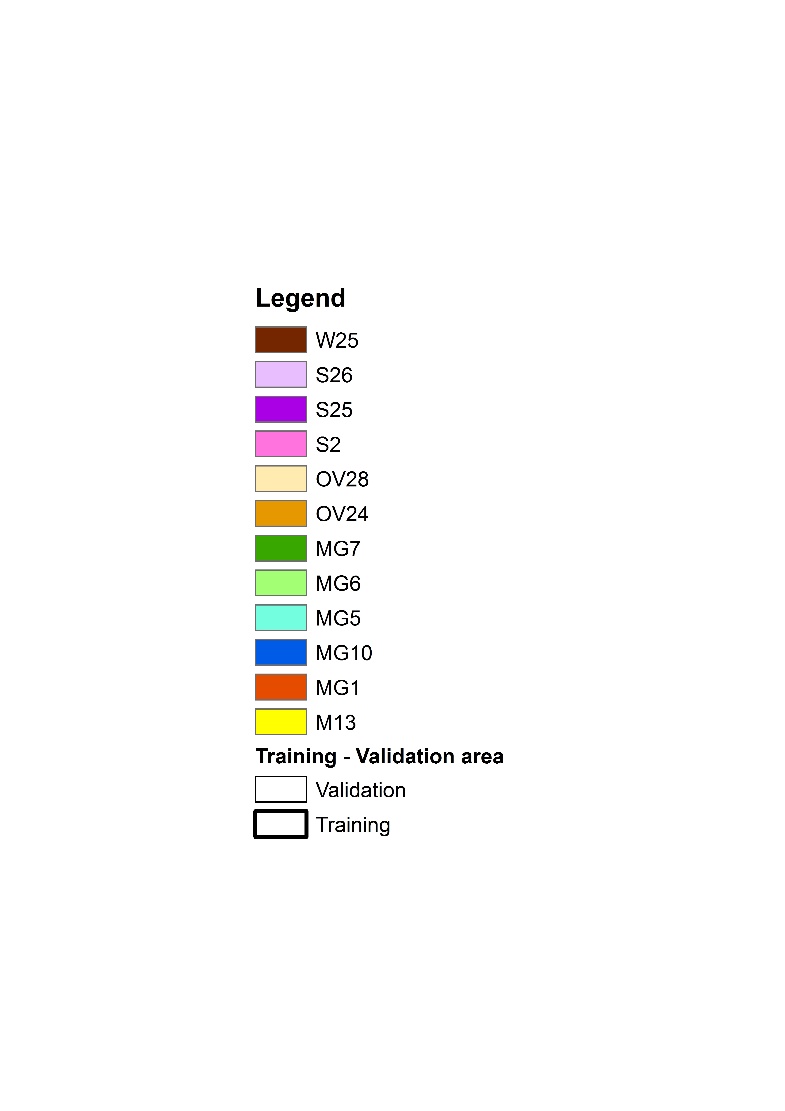


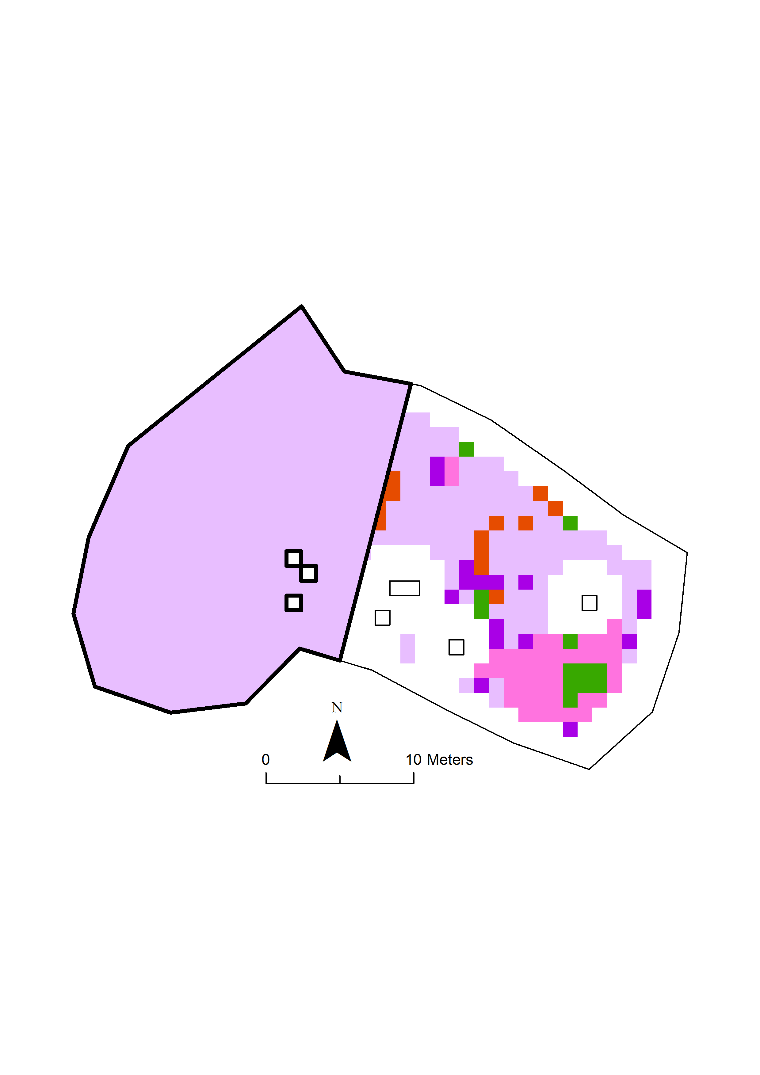

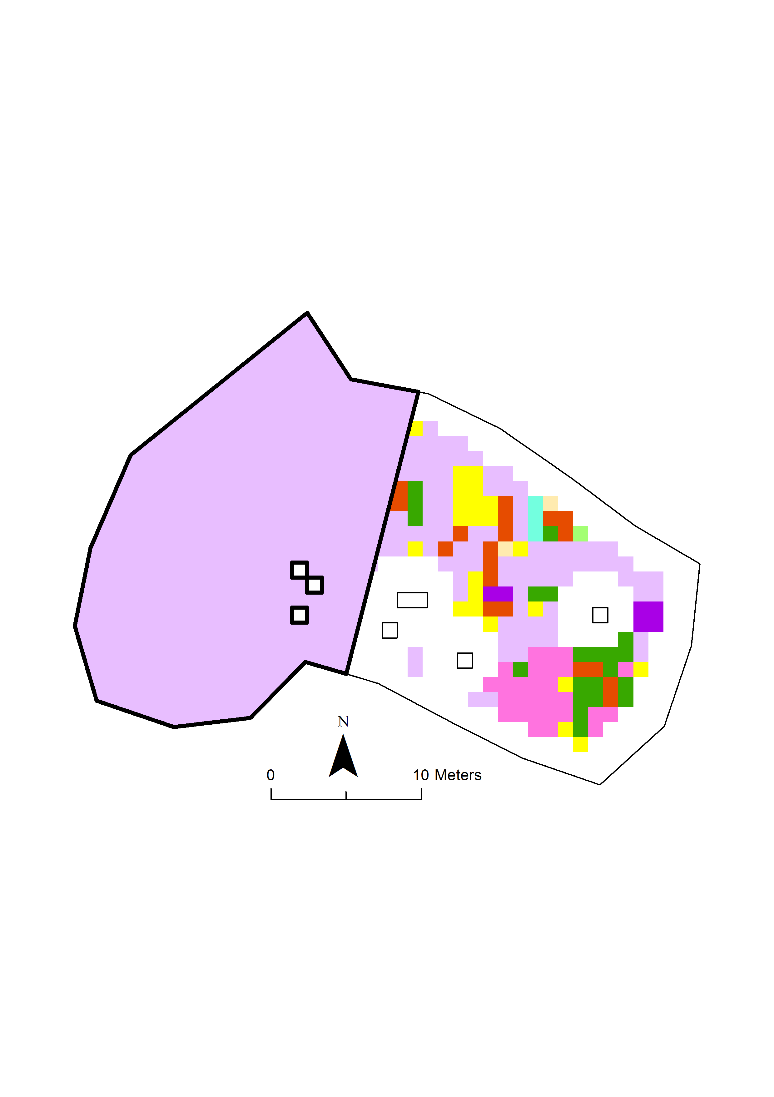

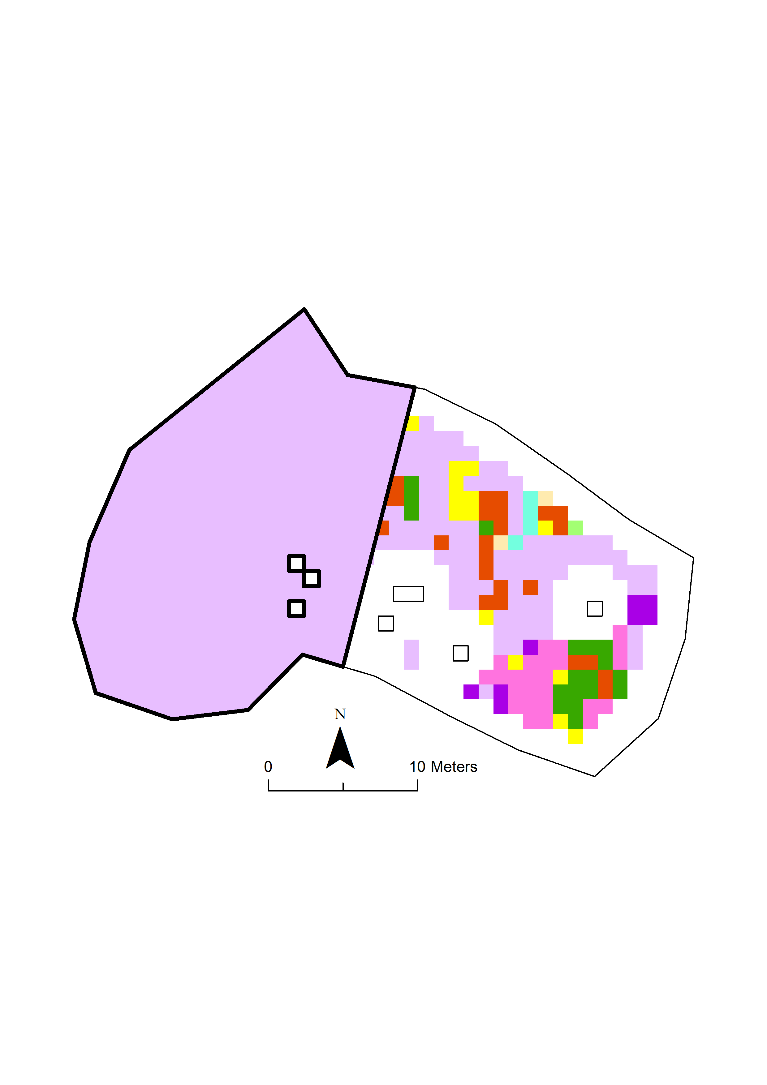


Hyperspectral Simulated 13-band Simulated 8-band


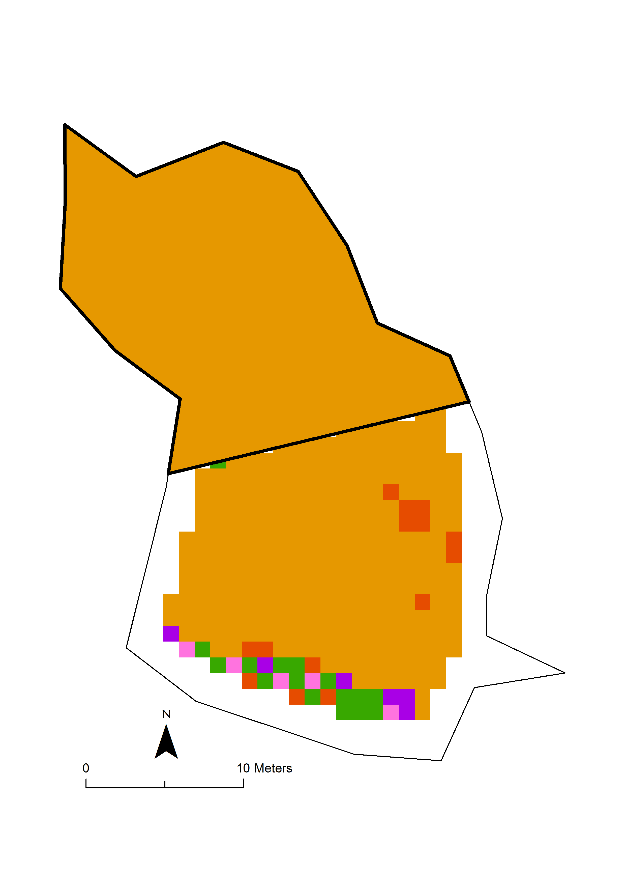

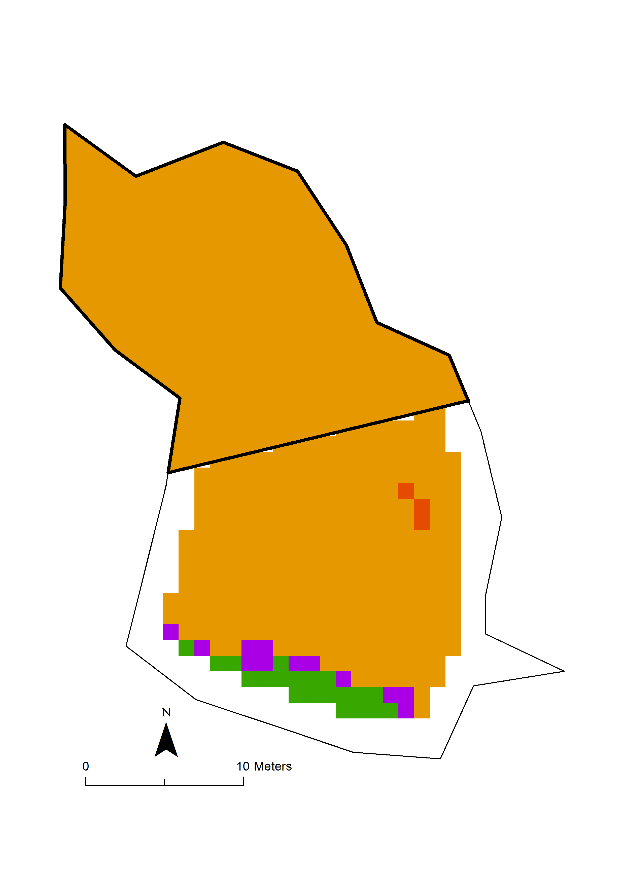

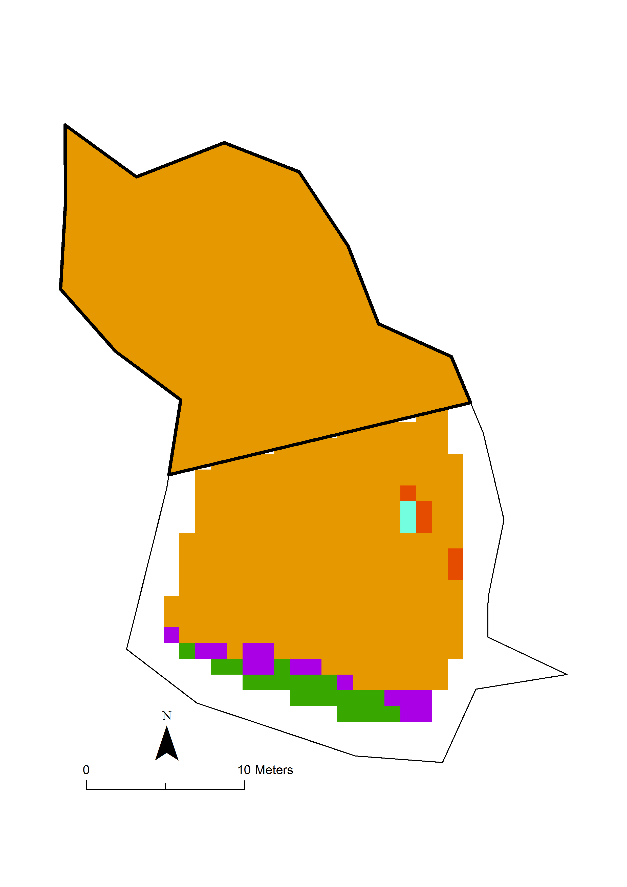

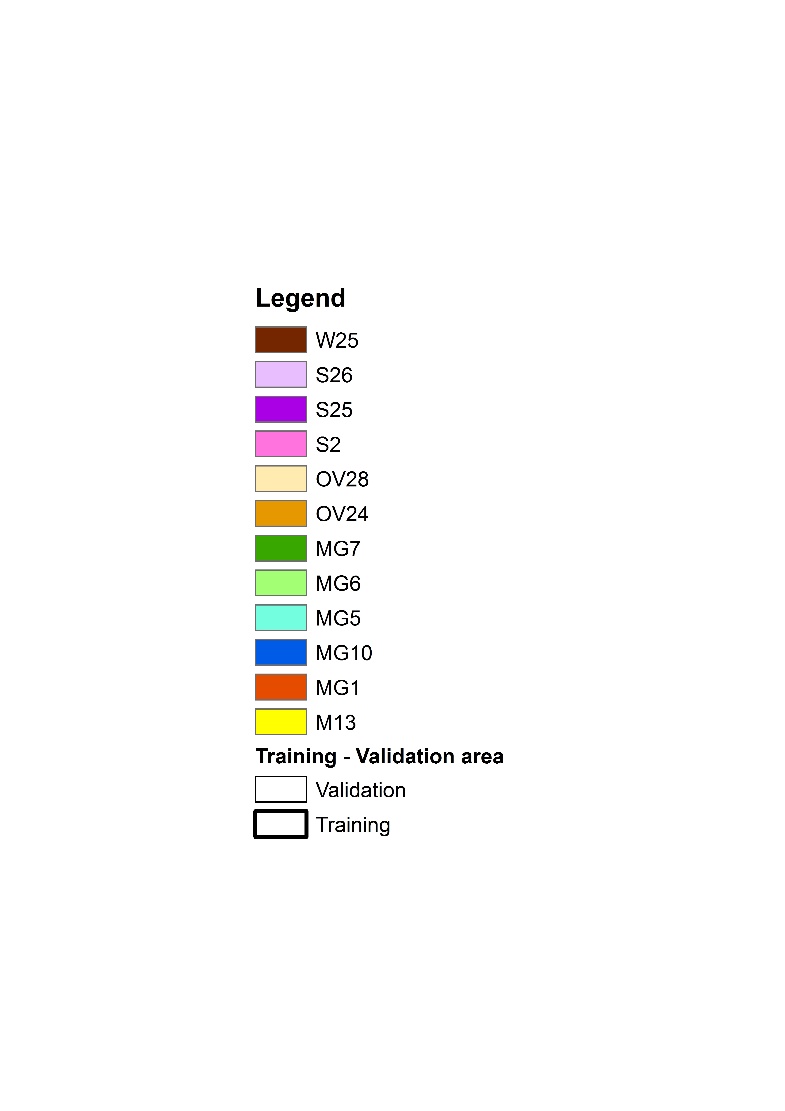


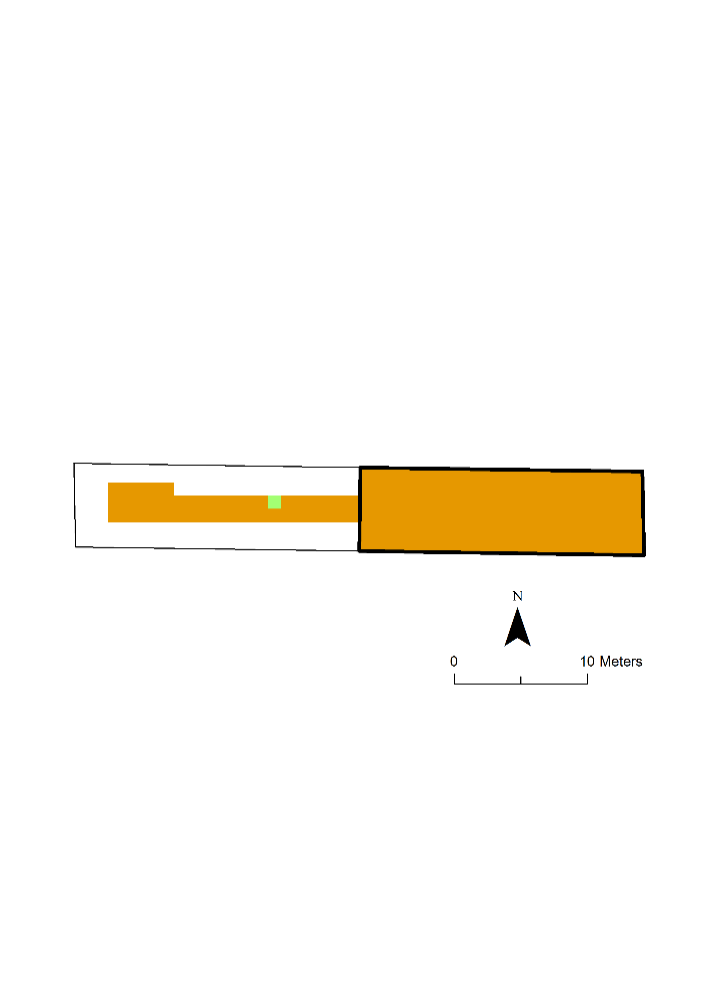

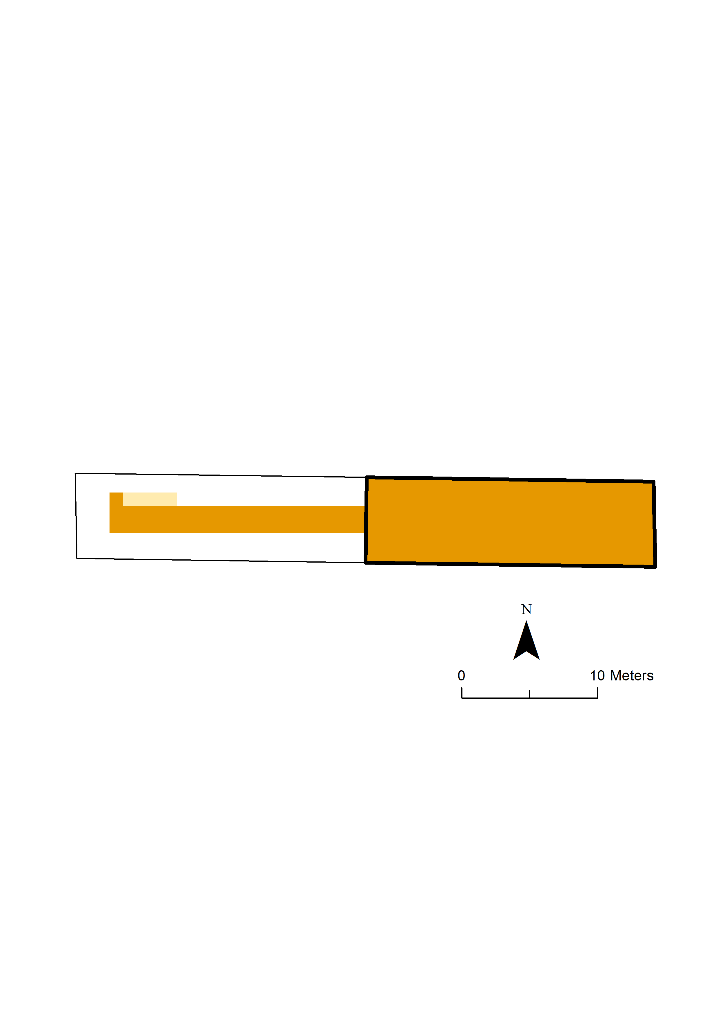

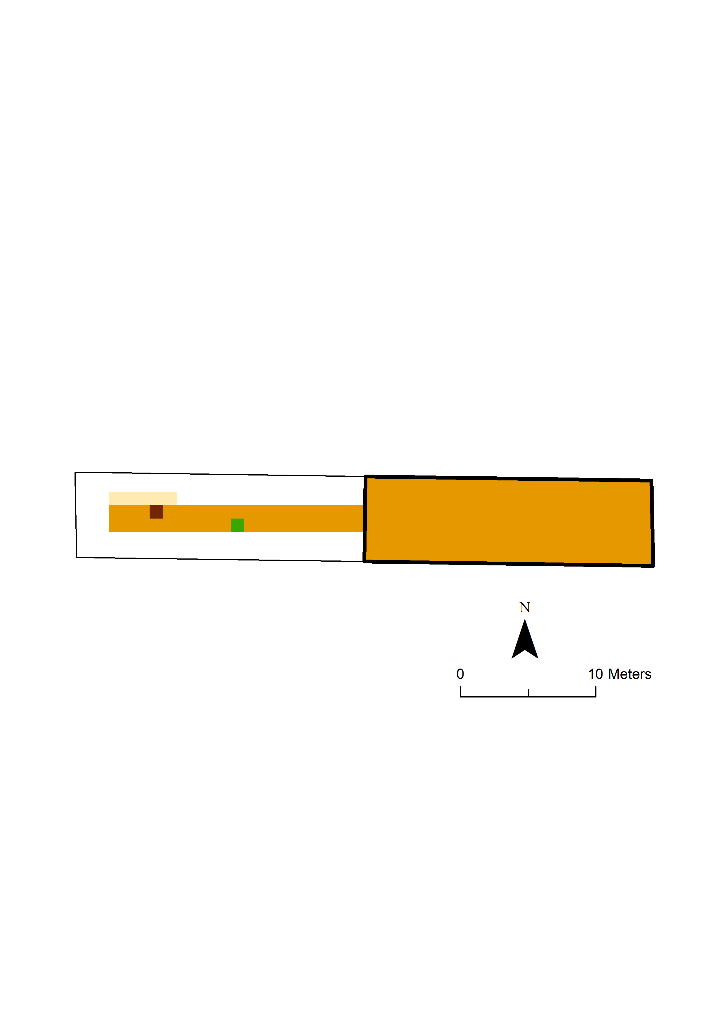


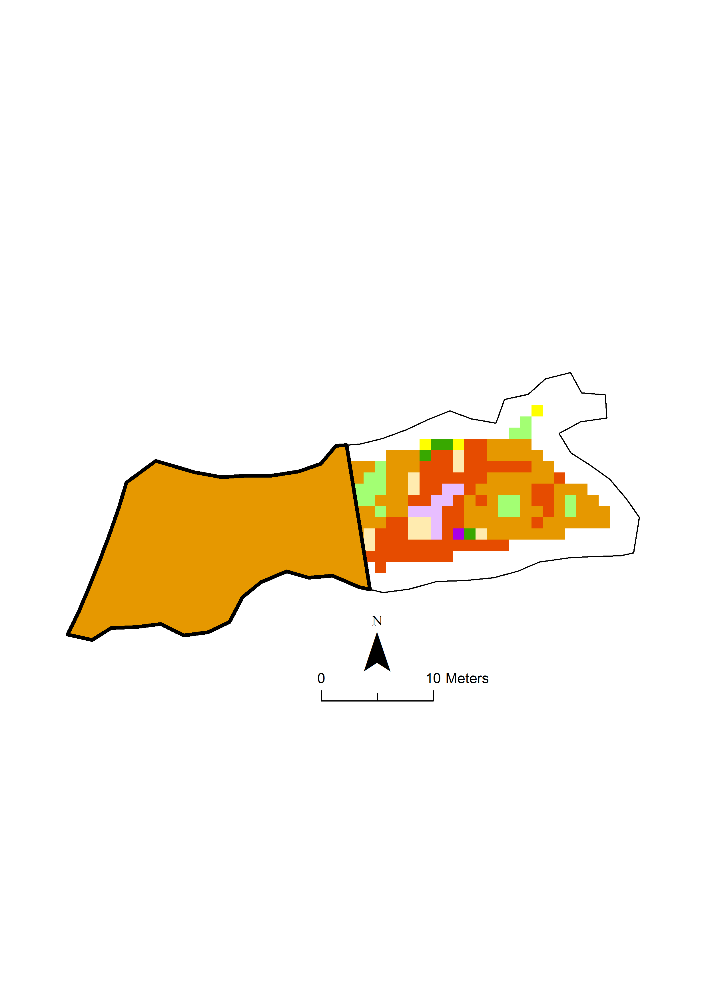

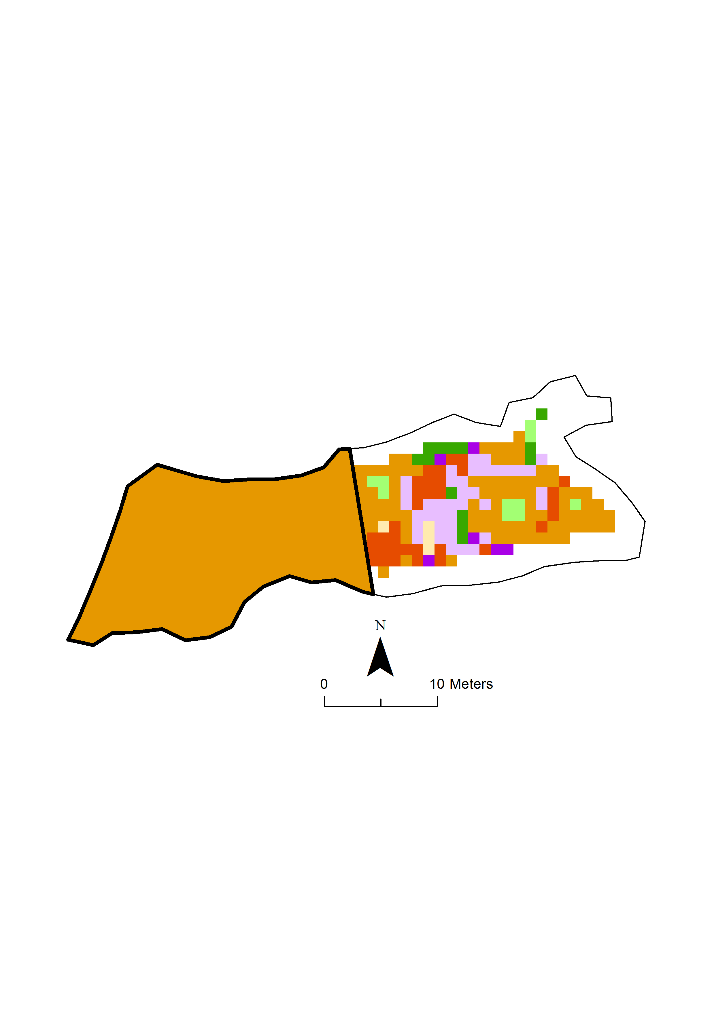

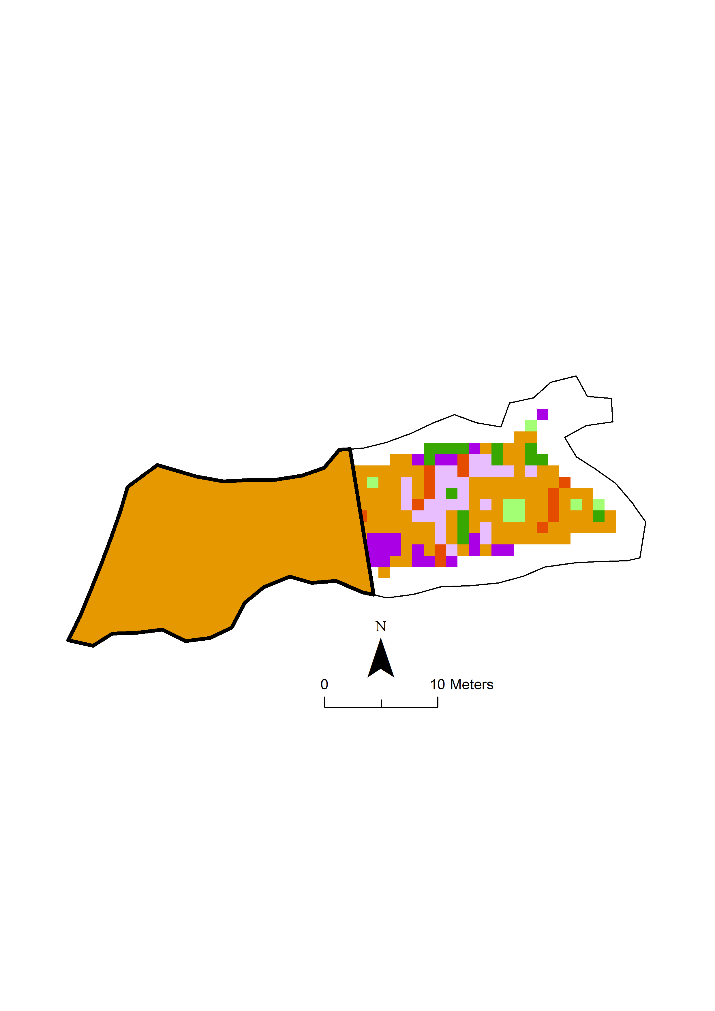


Hyperspectral Simulated 13-band Simulated 8-band


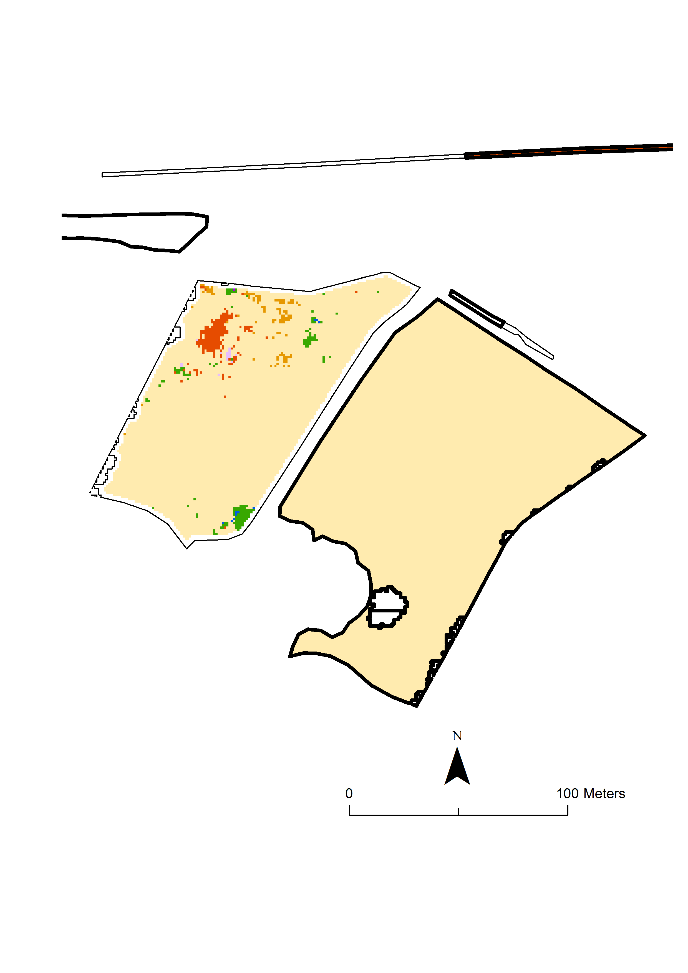

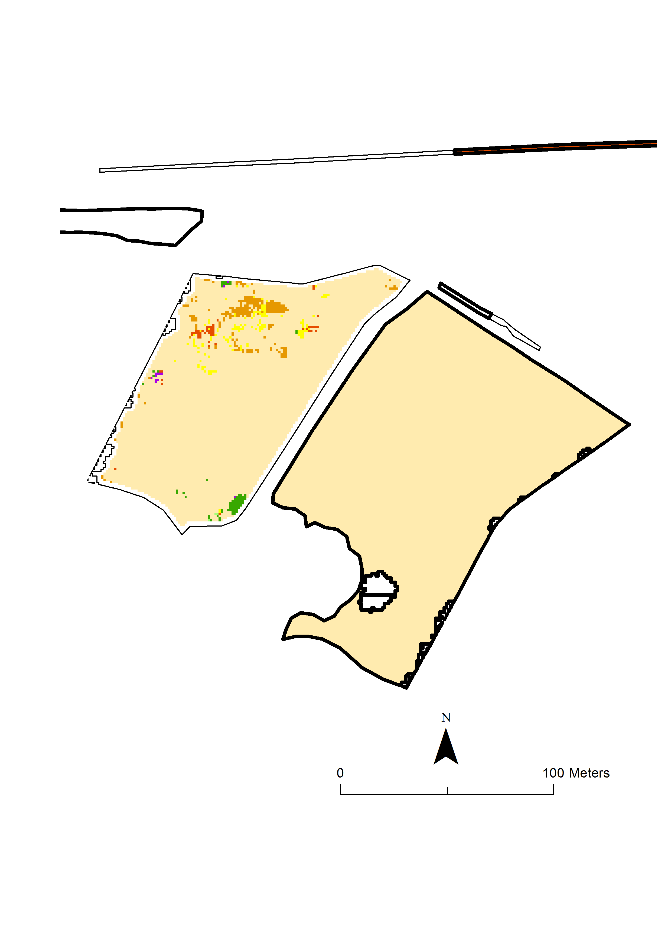

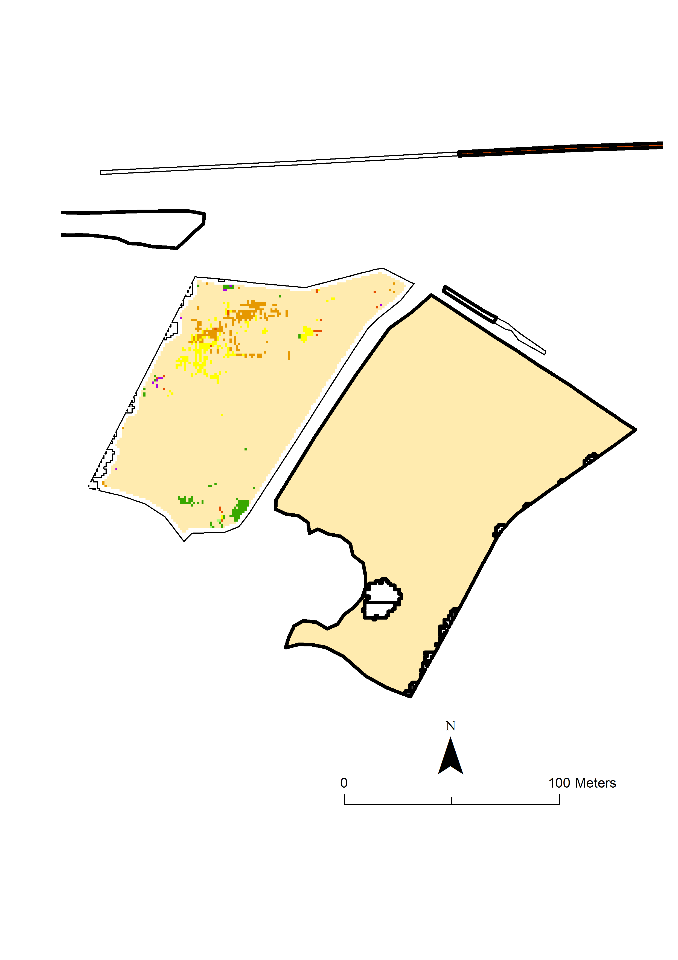

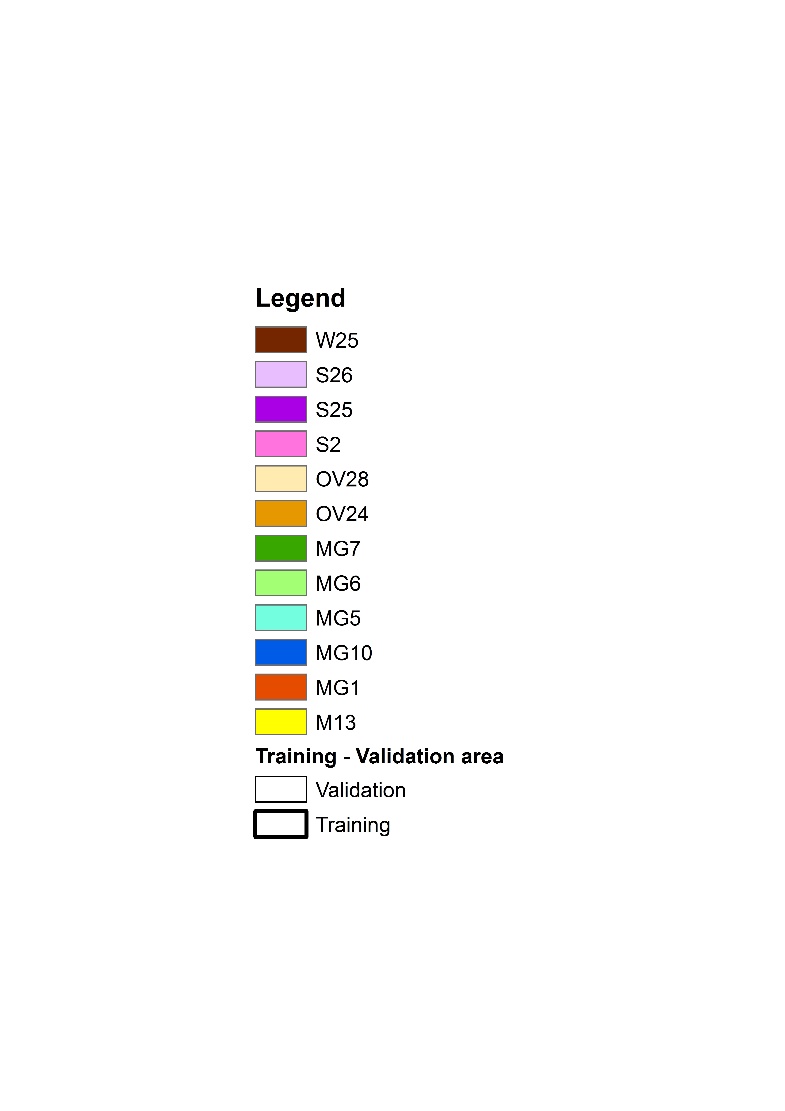


## Fig O 1

Further patches (in addition to those in Fig. 3 in the main manuscript) showing NVC communities used to train the random forest classifier (thick black line) and predicted NVC communities (thin black line) using hyperspectral (left), simulated 13-band (middle) and simulated 8-band data (right).
